# Methods for Evaluating Medical Tests and Biomarkers: Birmingham, UK. 19–20 July 2016

**DOI:** 10.1186/s41512-016-0001-y

**Published:** 2017-02-16

**Authors:** Gowri Gopalakrishna, Miranda Langendam, Rob Scholten, Patrick Bossuyt, Mariska Leeflang, Anna Noel-Storr, James Thomas, Iain Marshall, Byron Wallace, Penny Whiting, Clare Davenport, Mariska Leeflang, Gowri GopalaKrishna, Isabel de Salis, Sue Mallett, Robert Wolff, Penny Whiting, Richard Riley, Marie Westwood, Jos Kleinen, Gary Collins, Hans Reitsma, Karel Moons, Antonia Zapf, Annika Hoyer, Katharina Kramer, Oliver Kuss, J. Ensor, J. J. Deeks, E. C. Martin, R. D. Riley, Gerta Rücker, Susanne Steinhauser, Martin Schumacher, Richard Riley, Joie Ensor, Kym Snell, Brian Willis, Thomas Debray, Karel Moons, Jon Deeks, Gary Collins, Lavinia Ferrante di Ruffano, Brian Willis, Clare Davenport, Sue Mallett, Sian Taylor-Phillips, Chris Hyde, Jon Deeks, Sue Mallett, Stuart A. Taylor, Gauraang Batnagar, STREAMLINE COLON Investigators, STREAMLINE LUNG Investigators, METRIC Investigators, Sian Taylor-Phillips, Lavinia Ferrante Di Ruffano, Farah Seedat, Aileen Clarke, Jon Deeks, Sarah Byron, Frances Nixon, Rebecca Albrow, Thomas Walker, Carla Deakin, Chris Hyde, Zhivko Zhelev, Harriet Hunt, Lavinia Ferrante di Ruffano, Yaling Yang, Lucy Abel, James Buchanan, Thomas Fanshawe, Bethany Shinkins, Laure Wynants, Jan Verbakel, Sabine Van Huffel, Dirk Timmerman, Ben Van Calster, Mariska Leeflang, Aeliko Zwinderman, Patrick Bossuyt, Jason Oke, Jack O’Sullivan, Rafael Perera, Brian Nicholson, Hannah L. Bromley, Tracy E. Roberts, Adele Francis, Denniis Petrie, G. Bruce Mann, Kinga Malottki, Holly Smith, Jon Deeks, Lucinda Billingham, Alice Sitch, Sue Mallett, Jon Deeks, Oke Gerke, Mie Holm-Vilstrup, Eivind Antonsen Segtnan, Ulrich Halekoh, Poul Flemming Høilund-Carlsen, Bernard G. Francq, Jon Deeks, Alice Sitch, Jac Dinnes, Julie Parkes, Walter Gregory, Jenny Hewison, Doug Altman, William Rosenberg, Peter Selby, Julien Asselineau, Paul Perez, Aïssatou Paye, Emilie Bessede, Cécile Proust-Lima, Christiana Naaktgeboren, Joris de Groot, Anne Rutjes, Patrick Bossuyt, Johannes Reitsma, Karel Moons, Gary Collins, Emmanuel Ogundimu, Jonathan Cook, Yannick Le Manach, Doug Altman, Laure Wynants, Yvonne Vergouwe, Sabine Van Huffel, Dirk Timmerman, Ben Van Calster, Romin Pajouheshnia, Rolf Groenwold, Karen Moons, Johannes Reitsma, Linda Peelen, Ben Van Calster, Daan Nieboer, Yvonne Vergouwe, Bavo De Cock, Micael J. Pencina, Ewout W. Steyerberg, Jennifer Cooper, Sian Taylor-Phillips, Nick Parsons, Chris Stinton, Steve Smith, Andy Dickens, Rachel Jordan, Alexandra Enocson, David Fitzmaurice, Alice Sitch, Peymane Adab, Bernard G. Francq, Charles Boachie, Gaj Vidmar, Karoline Freeman, Martin Connock, Sian Taylor-Phillips, Rachel Court, Aileen Clarke, Joris de Groot, Christiana Naaktgeboren, Hans Reitsma, Carl Moons, Jessica Harris, Andrew Mumford, Zoe Plummer, Kurtis Lee, Barnaby Reeves, Chris Rogers, Veerle Verheyden, Gianni D. Angelini, Gavin J. Murphy, Jeremy Huddy, Melody Ni, Katherine Good, Graham Cooke, Patrick Bossuyt, George Hanna, Jie Ma, Doug Altman, Gary Collins, K. G. M. (Carl) Moons, Joris A. H. de Groot, Sue Mallett, Doug G. Altman, Johannes B. Reitsma, Gary S. Collins, Karel G. M. Moons, Douglas G. Altman, Johannes B. Reitsma, Gary S. Collins, Adina Najwa Kamarudin, Ruwanthi Kolamunnage-Dona, Trevor Cox, Melody Ni, Jeremy Huddy, Simone Borsci, George Hanna, Teresa Pérez, M.Carmen Pardo, Angel Candela-Toha, Alfonso Muriel, Javier Zamora, Sabina Sanghera, Syed Mohiuddin, Richard Martin, Jenny Donovan, Joanna Coast, Mikyung Kelly Seo, John Cairns, Elizabeth Mitchell, Alison Smith, Judy Wright, Peter Hall, Michael Messenger, Nicola Calder, Nyantara Wickramasekera, Karen Vinall-Collier, Andrew Lewington, Romin Pajouheshnia, Johanna Damen, Rolf Groenwold, Karel Moons, Linda Peelen, Michael Messenger, David Cairns, Alison Smith, Michelle Hutchinson, Judy Wright, Peter Hall, Nicola Calder, Cathie Sturgeon, Liz Mitchel, Rebecca Kift, Sofia Christakoudi, Manohursingh Rungall, Paula Mobillo, Rosa Montero, Tjir-Li Tsui, Sui Phin Kon, Beatriz Tucker, Steven Sacks, Chris Farmer, Terry Strom, Paramit Chowdhury, Irene Rebollo-Mesa, Maria Hernandez-Fuentes, Johanna A. A. G. Damen, Thomas P. A. Debray, Pauline Heus, Lotty Hooft, Karel G. M. Moons, Romin Pajouheshnia, Johannes B. Reitsma, Rob J. P. M. Scholten, Johanna A. A. G. Damen, Lotty Hooft, Ewoud Schuit, Thomas P. A. Debray, Gary S. Collins, Ioanna Tzoulaki, Camille M. Lassale, George C. M. Siontis, Virginia Chiocchia, Corran Roberts, Michael Maia Schlüssel, Stephen Gerry, James A. Black, Pauline Heus, Yvonne T. van der Schouw, Linda M. Peelen, Karel G. M. Moons, Johanna A. A. G. Damen, Thomas P. A. Debray, Pauline Heus, Lotty Hooft, Karel G. M. Moons, Romin Pajouheshnia, Johannes B. Reitsma, Rob J. P. M. Scholten, Jie Ma, Doug Altman, Gary Collins, Graeme Spence, David McCartney, Ann van den Bruel, Daniel Lasserson, Gail Hayward, Werner Vach, Antoinette de Jong, Coreline Burggraaff, Otto Hoekstra, Josée Zijlstra, Henrica de Vet, Harriet Hunt, Chris Hyde, Sara Graziadio, Joy Allen, Louise Johnston, Rachel O’Leary, Michael Power, Joy Allen, Sara Graziadio, Louise Johnson, Rachel O’Leary, Michael Power, Ray Waters, John Simpson, Louise Johnston, Joy Allen, Sara Graziadio, Rachel O’Leary, Ray Waters, Michael Power, Sue Mallett, Thomas R. Fanshawe, Peter Phillips, Andrew Plumb, Emma Helbren, Steve Halligan, Stuart A. Taylor, Alastair Gale, Sue Mallett, Peggy Sekula, Douglas G. Altman, Willi Sauerbrei, Sue Mallett, Thomas R. Fanshawe, Julia R. Forman, Susan J. Dutton, Yemisi Takwoingi, Elizabeth M. Hensor, Thomas E. Nichols, Bethany Shinkins, Yaling Yang, Lucy Abel, Lavinia Ferrante Di Ruffano, Thomas Fanshawe, Emmanuelle Kempf, Raphael Porcher, Jennifer de Beyer, Karel Moons, Douglas Altman, Hans Reitsma, Sally Hopewell, Willi Sauerbrei, Gary Collins, John Dennis, Beverley Shields, Angus Jones, William Henley, Ewan Pearson, Andrew Hattersley, on behalf of the MASTERMIND consortium, Pauline Heus, Johanna A. A. G. Damen, Romin Pajouheshnia, Rob J. P. M. Scholten, Johannes B. Reitsma, Gary S. Collins, Douglas G. Altman, Karel G. M. Moons, Lotty Hooft, Beverley Shields, John Dennis, Angus Jones, William Henley, Ewan Pearson, Andrew Hattersley, on behalf of the MASTERMIND consortium, Fueloep Scheibler, Anne Rummer, Sibylle Sturtz, Robert Großelfinger, Katie Banister, Craig Ramsay, Augusto Azuara-Blanco, Jonathan Cook, Charles Boachie, Jennifer Burr, Manjula Kumarasamy, Rupert Bourne, Ijeoma Uchegbu, Simone Borsci, Jennifer Murphy, George Hanna, Ijeoma Uchegbu, Alex Carter, Jen Murphy, Melody Ni, Joachim Marti, Julie Eatock, Ijeoma Uchegbu, Julie Robotham, Maria Dudareva, Mark Gilchrist, Alison Holmes, Ijeoma Uchegbu, Simone Borsci, Phillip Monaghan, Sarah Lord, Andrew StJohn, Sverre Sandberg, Christa Cobbaert, Lieselotte Lennartz, Wilma Verhagen-Kamerbeek, Christoph Ebert, Patrick Bossuyt, Andrea Horvath, for the Test Evaluation Working Group of the European Federation of Clinical Chemistry and Laboratory Medicine, Kevin Jenniskens, Christiana Naaktgeboren, Johannes Reitsma, Karel Moons, Joris de Groot, Chris Hyde, Jaime Peters, Bogdan Grigore, Jaime Peters, Chris Hyde, Chris Hyde, Obi Ukoumunne, Jaime Peters, Zhivko Zhelev, Brooke Levis, Andrea Benedetti, Alexander W. Levis, John P. A. Ioannidis, Ian Shrier, Pim Cuijpers, Simon Gilbody, Lorie A. Kloda, Dean McMillan, Scott B Patten, Russell J. Steele, Roy C Ziegelstein, Charles H. Bombardier, Flavia de Lima Osório, Jesse R. Fann, Dwenda Gjerdingen, Femke Lamers, Manote Lotrakul, Sonia R Loureiro, Bernd Löwe, Juwita Shaaban, Lesley Stafford, Henk C. P. M. van Weert, Mary A. Whooley, Linda S. Williams, Karin A. Wittkampf, Albert S. Yeung, Brett D. Thombs, Jaime Peters, Chris Cooper, James Buchanan, Tom Nieto, Claire Smith, Olga Tucker, Janine Dretzke, Andrew Beggs, Nirmala Rai, Clare Davenport, Sue Bayliss, Simon Stevens, Kym Snell, Sue Mallet, Jon Deeks, Sudha Sundar, Emma Hall, Nuria Porta, David Lorente Estelles, Johann de Bono, on behalf of the CTC-STOP protocol development group

**Affiliations:** 1 Department of Clinical Epidemiology, Biostatistics & Bioinformatics, Academic Medical Center, University of Amsterdam, Amsterdam, The Netherlands; 2 Cochrane Netherlands, Julius Center for Health Sciences and Primary Care, University Medical Center Utrecht, Utrecht, Netherlands; 3 Cochrane Dementia and Cognitive Improvement Group, University of Oxford, Oxford, UK; 4 EPPI-Centre, Department of Social Science, University College London, London, UK; 5 Division of Health and Social Care Research, King’s College London, London, UK; 6 College of Computer and Information Science, Northeastern University, Boston, USA; 7 University Hospitals Bristol NHS Foundation Trust, School of Social and Community Medicine, Bristol, UK; 8 Institute of Applied Health Research, University of Birmingham, Birmingham, UK; 9 Department of Clinical Epidemiology, Biostatistics & Bioinformatics, Academic Medical Center, University of Amsterdam, Amsterdam, The Netherlands; 10 School of Social and Community Medicine, University of Bristol, Bristol, UK; 11 Institute of Applied Health Research, University of Birmingham, Birmingham, UK; 12 Kleijnen Systematic Reviews Ltd, York, UK; 13 University Hospitals Bristol NHS Foundation Trust, School of Social and Community Medicine, Bristol, UK; 14 Research Institute for Primary Care and Health Sciences, Keele University, Keele, UK; 15 Centre for Statistics in Medicine, Nuffield Department of Orthopaedics, Rheumatology and Musculoskeletal Sciences, University of Oxford, Oxford, UK; 16 Julius Center for Health Sciences and Primary Care, University Medical Center Utrecht, Utrecht, The Netherlands; 17 Cochrane Netherlands, University Medical Center Utrecht, Utrecht, The Netherlands; 18 Department of Medical Statistics, University Medical Center Göttingen, Göttingen, Germany; 19 Institute for Biometry and Epidemiology, German Diabetes Center, Leibniz Institute for Diabetes Research at Heinrich Heine University, Düsseldorf, Germany; 20 Research Institute for Primary Care and Health Sciences, Keele University, Keele, UK; 21 Institute of Applied Health Research, University of Birmingham, Birmingham, UK; 22 Manchester Pharmacy School, University of Manchester, Manchester, UK; 23 Institute for Medical Biometry and Statistics, Faculty of Medicine and Medical Center – University of Freiburg, Stefan-Meier-Str. 26, 79104 Freiburg, Germany; 24 Institute of Medical Statistics, Informatics and Epidemiology, University of Cologne, Kerpener Str. 62, 50937 Cologne, Germany; 25 Research Institute for Primary Care and Health Sciences, Keele University, Keele, UK; 26 Keele University, Keele, UK; 27 Institute of Applied Health Research, University of Birmingham, Birmingham, UK; 28 University of Utrecht, Utrecht, Netherlands; 29 Julius Center for Health Sciences and Primary Care, University Medical Center Utrecht, 3584 CG Utrecht, Netherlands; 30 Centre for Statistics in Medicine, Nuffield Department of Orthopaedics, Rheumatology and Musculoskeletal Sciences, University of Oxford, Oxford, UK; 31 Institute of Applied Health Research, University of Birmingham, Birmingham, UK; 32 Division of Health Sciences, Warwick Medical School, The University of Warwick, Coventry, UK; 33 Exeter Test Group, University of Exeter Medical School, Exeter, UK; 34 Institute of Applied Health Research, University of Birmingham, Birmingham, UK; 35 Centre for Medical Imaging, University College London, London, UK; 36 Division of Health Sciences, Warwick Medical School, The University of Warwick, Coventry, UK; 37 Institute of Applied Health Research, University of Birmingham, Birmingham, UK; 38 University of Warwick, Coventry, UK; 39 National Institute for Health and Care Excellence, Diagnostic Assessment Programme, Manchester, UK; 40 Exeter Test Group, University of Exeter Medical School, Exeter, UK; 41 Institute of Applied Health Research, University of Birmingham, Birmingham, UK; 42 Nuffield Department of Primary Health Care Sciences, University of Oxford, Oxford, UK; 43 Academic Unit of Health Economics, Leeds Institute of Health Sciences, University of Leeds, Leeds, UK; 44 KU Leuven Department of Electrical Engineering (ESAT), STADIUS Center for Dynamical Systems, Signal Processing and Data Analytics, Leuven, Belgium; 45 KU Leuven iMinds Department Medical Information Technologies, Leuven, Belgium; 46 University of Oxford, Nuffield Department of Primarcy Care Health Sciences, Oxford, UK; 47 KU Leuven Department of Development and Regeneration, Leuven, Belgium; 48 Department of Public Health, Erasmus MC, Rotterdam, Netherlands; 49 Department of Clinical Epidemiology, Biostatistics & Bioinformatics, Academic Medical Center, University of Amsterdam, Amsterdam, The Netherlands; 50 Nuffield Department of Primary Care Health Sciences, University of Oxford, Oxford, UK; 51 Department of Health Economics, University of Birmingham, Birmingham, UK; 52 Department of Breast Surgery, Nuffield House, University Hospital Birmingham, Birmingham, UK; 53 Melbourne School of Population and Global Health, University of Melbourne, Parkville, Australia; 54 Department of Breast Surgery, Royal Women’s Hospital, Melbourne, Australia; 55 Cancer Research UK Clinical Trials Unit (CRCTU), Institute of Cancer and Genomic Sciences, University of Birmingham, Birmingham, UK; 56 Institute of Applied Health Research, University of Birmingham, Birmingham, UK; 57 Institute of Applied Health Research, University of Birmingham, Birmingham, UK; 58 Department of Nuclear Medicine, Odense University Hospital, Odense, Denmark; 59 Epidemiology, Biostatistics and Biodemography, University of Southern Denmark, Odense, Denmark; 60 Department of Nuclear Medicine, Odense University Hospital & Department of Clinical Research, University of Southern Denmark, Odense, Denmark; 61 Robertson Centre for Biostatistics, University of Glasgow, Glasgow, UK; 62 Institute of Applied Health Research, University of Birmingham, Birmingham, UK; 63 University of Southampton, Southampton, UK; 64 University of Leeds, Leeds, UK; 65 Centre for Statistics in Medicine, Nuffield Department of Orthopaedics, Rheumatology and Musculoskeletal Sciences, University of Oxford, Oxford, UK; 66 University College London, London, UK; 67 Bordeaux University Hospital, Public Health Department, Clinical Epidemiology Unit and CIC 1401 EC, Bordeaux, France; 68 French National Reference Center for Campylobacter and Helicobacter, Bordeaux, France; 69 INSERM U1219, Bordeaux Population Health Research Center, Bordeaux, France; Univ. Bordeaux, ISPED, Bordeaux, France; 70 Julius Center for Health Sciences and Primary Care, University Medical Center Utrecht, 3584 CG Utrecht, Netherlands; 71 CTU Bern, Department of Clinical Research, University of Bern, Bern, Switzerland; 72 Institute of Social and Preventive Medicine, University of Bern, Bern, Switzerland; 73 Department of Clinical Epidemiology and Biostatistics, Academic Medical Center, University of Amsterdam, Amsterdam, Netherlands; 74 Centre for Statistics in Medicine, Nuffield Department of Orthopaedics, Rheumatology and Musculoskeletal Sciences, University of Oxford, Oxford, UK; 75 Departments of Anesthesia & Clinical Epidemiology and Biostatistics, Michael DeGroote School of Medicine, Faculty of Health Sciences, McMaster University and the Perioperative Research Group, Population Health Research Institute, Hamilton, Canada; 76 KU Leuven Department of Electrical Engineering (ESAT), STADIUS Center for Dynamical Systems, Signal Processing and Data Analytics, Leuven, Belgium; 77 KU Leuven iMinds Department Medical Information Technologies, Leuven, Belgium; 78 Center for Medical Decision Sciences, Department of Public Health, Erasmus MC, Rotterdam, The Netherlands; 79 KU Leuven Department of Development and Regeneration, Leuven, Belgium; 80 Department of Public Health, Erasmus MC, Rotterdam, Netherlands; 81 Julius Center for Health Sciences and Primary Care, University Medical Center Utrecht, Utrecht, The Netherlands; 82 Cochrane Netherlands, University Medical Center Utrecht, Utrecht, The Netherlands; 83 KU Leuven, Department of Development and Regeneration, Leuven, Belgium; 84 Department of Public Health, Erasmus MC, Rotterdam, Netherlands; 85 Duke Clinical Research Institute, Duke University, Durham (NC), USA; Department of Biostatistics and Bioinformatics, Duke University, Durham, NC USA; 86 Division of Health Sciences, Warwick Medical School, University of Warwick, Coventry, UK; 87 Midlands and North West Bowel Cancer Screening Hub, Hospital of St Cross, University Hospitals Coventry and Warwickshire NHS Trust, Rugby, UK; 88 Institute of Applied Health Research, University of Birmingham, Birmingham, UK; 89 Robertson Centre for Biostatistics, University of Glasgow, Glasgow, UK; 90 University Rehabilitation Institute, Ljubljana, Slovenia; 91 Faculty of Medicine, University of Ljubljana, Ljubljana, Slovenia; 92 University of Primorska, Koper, Slovenia; 93 Division of Health Sciences, Warwick Medical School, The University of Warwick, Coventry, UK; 94 Julius Center for Health Sciences and Primary Care, University Medical Center Utrecht, 3584 CG Utrecht, Netherlands; 95 Clinical Trials and Evaluation Unit, University of Bristol, Bristol, UK; 96 School of Cellular and Molecular Medicine, University of Bristol, Bristol, UK; 97 University Hospitals Bristol NHS Foundation Trust, Bristol, UK; 98 School of Clinical Sciences, University of Bristol, Bristol, UK; 99 Department of Clinical Sciences, University of Leicester, Leicester, UK; 100 NIHR-Diagnostic Evidence Cooperative, Imperial College London, London, UK; 101 Department of Anaesthetics, Imperial College Healthcare NHS Trust, London, UK; 102 Division of Infectious Diseases, Imperial College, London, UK; 103 Department of Clinical Epidemiology, Biostatistics & Bioinformatics, Academic Medical Center, University of Amsterdam, Amsterdam, The Netherlands; 104 Centre for Statistics in Medicine, Nuffield Department of Orthopaedics, Rheumatology and Musculoskeletal Sciences, University of Oxford, Oxford, UK; 105 Julius Center for Health Sciences and Primary Care, University Medical Center Utrecht, 3584 CG Utrecht, Netherlands; 106 Institute of Applied Health Research, University of Birmingham, Birmingham, UK; 107 Centre for Statistics in Medicine, Nuffield Department of Orthopaedics, Rheumatology and Musculoskeletal Sciences, University of Oxford, Oxford, UK; 108 Cochrane Netherlands, University Medical Center Utrecht, Utrecht, The Netherlands; 109 Julius Center for Health Sciences and Primary Care, University Medical Center Utrecht, 3584 CG Utrecht, Netherlands; 110 Centre for Statistics in Medicine, Nuffield Department of Orthopaedics, Rheumatology and Musculoskeletal Sciences, University of Oxford, Oxford, UK; 111 Cochrane Netherlands, University Medical Center Utrecht, Utrecht, The Netherlands; 112 Department of Biostatistics, University of Liverpool, Liverpool, UK; 113 NIHR-Diagnostic Evidence Cooperative, Imperial College London, London, UK; 114 Department of Statistics and OR III, Complutense University of Madrid, Madrid, Spain; 115 Department of Statistics and OR I, Complutense University of Madrid, Madrid, Spain; 116 Department of Anesthesia and Reanimation, Ramón y Cajal Hospital (IRYCIS), Madrid, Spain; 117 Department of Clinical Biostatistics, Ramón y Cajal Hospital (IRYCIS), Madrid, Spain; 118 Department of Clinical Biostatistics, Ramón y Cajal Hospital (IRYCIS), Madrid, Spain; 119 Barts and The London School of Medicine and Dentistry, Queen Mary University, London, UK; 120 Health Economics at Bristol (HEB), School of Social and Community Medicine, University of Bristol, Bristol, UK; 121 School of Social and Community Medicine, University of Bristol, Bristol, UK; 122 London School of Hygiene and Tropical Medicine, London, UK; 123 University of Bergen, Bergen, Norway; 124 Leeds Institute of Health Sciences, University of Leeds, Leeds, UK; 125 NIHR Diagnostic Evidence Co-Operative Leeds , Leeds Institute of Health Sciences, University of Leeds, Leeds, UK; 126 The Institute of Genetics and Molecular Medicine, Edinburgh Cancer Research Centre, University of Edinburgh, Edinburgh, UK; 127 Leeds Teaching Hospitals NHS Trust, St James’s University Hospital, Leeds, UK; 128 School of Dentistry, University of Leeds, Leeds, UK; 129 Julius Center for Health Sciences and Primary Care, University Medical Center Utrecht, Utrecht, The Netherlands; 130 Cochrane Netherlands, University Medical Center Utrecht, Utrecht, The Netherlands; 131 NIHR Diagnostic Evidence Co-Operative Leeds, Leeds Teaching Hospitals NHS Trust, St James’s University Hospital, Leeds, UK; 132 Leeds Institute of Clinical Trials Research, University of Leeds, Leeds, UK; 133 Leeds Institute of Health Sciences, University of Leeds, Leeds, UK; 134 Leeds Institute of Cancer and Pathology, University of Leeds, Leeds, UK; 135 The Institute of Genetics and Molecular Medicine, Edinburgh Cancer Research Centre, University of Edinburgh, Edinburgh, UK; 136 UK NEQAS [Edinburgh]. Department of Laboratory Medicine, Royal Infirmary, Edinburgh, UK; 137 Department of Blood Sciences, Old Medical School, Leeds General Infirmary, Great George Street, Leeds, UK; 138 MRC Centre for Transplantation, DTIMB, King’s College London, London, UK; 139 NIHR Comprehensive Biomedical Research Centre at Guy’s and St Thomas’ NHS Foundation Trust in partnership with King’s College London and King’s College Hospital, London, UK; 140 Guy’s and St Thomas’ NHS Foundation Trust, London, UK; 141 King’s College Hospital NHS Foundation Trust, London, UK; 142 East Kent Hospitals University NHS Foundation Trust, Canterbury, UK; 143 Department of Medicine, Transplant Institute, Beth Israel Deaconess Medical Center, Harvard Medical School, Boston, MA USA; 144 Department of Biostatistics, Institute of Psychiatry, Psychology and Neuroscience, King’s College London, London, UK; 145 Julius Center for Health Sciences and Primary Care, and Cochrane Netherlands, University Medical Center Utrecht, Utrecht, The Netherlands; 146 Cochrane Netherlands, Julius Center for Health Sciences and Primary Care, University Medical Center Utrecht, Utrecht, The Netherlands; 147 Julius Center for Health Sciences and Primary Care, University Medical Center Utrecht, 3584 CG Utrecht, Netherlands; 148 Julius Center for Health Sciences and Primary Care, and Cochrane Netherlands, University Medical Center Utrecht, Utrecht, The Netherlands; 149 Cochrane Netherlands, Julius Center for Health Sciences and Primary Care, University Medical Center Utrecht, Utrecht, The Netherlands; 150 Centre for Statistics in Medicine, Nuffield Department of Orthopaedics, Rheumatology and Musculoskeletal Sciences, University of Oxford, Oxford, UK; 151 Department of Epidemiology and Biostatistics, School of Public Health, Imperial College London, London, UK; 152 Department of Cardiology, Bern University Hospital, 3010 Bern, Switzerland; 153 MRC Epidemiology Unit, University of Cambridge School of Clinical Medicine, Cambridge, UK; 154 Julius Center for Health Sciences and Primary Care, University Medical Center Utrecht, Utrecht, Netherlands; 155 Julius Center for Health Sciences and Primary Care, and Cochrane Netherlands, University Medical Center Utrecht, Utrecht, The Netherlands; 156 Cochrane Netherlands, Julius Center for Health Sciences and Primary Care, University Medical Center Utrecht, Utrecht, The Netherlands; 157 Julius Center for Health Sciences and Primary Care, University Medical Center Utrecht, 3584 CG Utrecht, Netherlands; 158 Centre for Statistics in Medicine, Nuffield Department of Orthopaedics, Rheumatology and Musculoskeletal Sciences, University of Oxford, Oxford, UK; 159 Nuffield Department of Primary Care Health Sciences, University of Oxford, Oxford, UK; 160 Nuffield Department of Medicine, University of Oxford, Oxford, UK; 161 Institute for Medical Biometry and Statistics, Medical Faculty and Medical Center, University of Freiburg, Freiburg im Breisgau, Germany; 162 Department of Radiology and Nuclear Medicine, University Medical Center, Utrecht, The Netherlands; 163 Department of Hematology, VU University Medical Center, Amsterdam, The Netherlands; 164 Department of epidemiology and biostatistics, VU University Medical Center, Amsterdam, The Netherlands; 165 Exeter Test Group, University of Exeter Medical School, Exeter, UK; 166 NIHR Diagnostic Evidence Co-operative Newcastle, Newcastle upon Tyne Hospitals NHS Foundation Trust, Newcastle upon Tyne, UK; 167 NIHR Diagnostic Evidence Co-operative Newcastle, Newcastle University, Newcastle upon Tyne, UK; 168 NIHR Diagnostic Evidence Co-operative Newcastle, Newcastle University, Newcastle upon Tyne, UK; 169 NIHR Diagnostic Evidence Co-operative Newcastle, Newcastle upon Tyne Hospitals NHS Foundation Trust, Newcastle upon Tyne, UK; 170 NIHR Diagnostic Evidence Co-operative Newcastle, Newcastle University, Newcastle upon Tyne, UK; 171 NIHR Diagnostic Evidence Co-operative Newcastle, Newcastle upon Tyne Hospitals NHS Foundation Trust, Newcastle upon Tyne, UK; 172 Institute of Applied Health Research, University of Birmingham, Birmingham, UK; 173 Nuffield Department of Primary Health Care Sciences, University of Oxford, Oxford, UK; 174 Applied Vision Research Centre, Loughborough University, Loughborough, UK; 175 Centre for Medical Imaging, University College London, London, UK; 176 Institute of Applied Health Research, University of Birmingham, Birmingham, UK; 177 Institute for Medical Biometry and Statistics, Faculty of Medicine and Medical Center – University of Freiburg, Freiburg im Breisgau, Germany; 178 Centre for Statistics in Medicine, Nuffield Department of Orthopaedics, Rheumatology and Musculoskeletal Sciences, University of Oxford, Oxford, UK; 179 Institute of Applied Health Research, University of Birmingham, Birmingham, UK; 180 Nuffield Department of Primary Health Care Sciences, University of Oxford, Oxford, UK; 181 Cambridge Clinical Trials Unit, Cambridge University Hospitals, Cambridge, UK; 182 Centre for Statistics in Medicine & Oxford Clinical Trials Research Unit, Nuffield Department of Orthopaedics, Rheumatology and Musculoskeletal Sciences, University of Oxford, Oxford, UK; 183 NIHR Leeds Musculoskeletal Biomedical Research Unit & Leeds Institute of Rheumatic and Musculoskeletal Medicine, University of Leeds, Leeds, UK; 184 Department of Statistics & Warwick Manufacturing Group, University of Warwick, Warwick, UK; 185 Academic Unit of Health Economics, Leeds Institute of Health Sciences, University of Leeds, Leeds, UK; 186 Nuffield Department of Primary Health Care Sciences, University of Oxford, Oxford, UK; 187 Institute of Applied Health Research, University of Birmingham, Birmingham, UK; 188 Centre for Statistics in Medicine, Nuffield Department of Orthopaedics, Rheumatology and Musculoskeletal Sciences, University of Oxford, Oxford, UK; 189 Department of Medical Oncology, Henri Mondor & Albert Chenevier Teaching Hospital, APHP, Créteil, France; 190 Department of Epidemiology, Hôtel Dieu Teaching Hospital, APHP, Paris, France; 191 Julius Center for Health Sciences and Primary Care, University Medical Center Utrecht, 3584 CG Utrecht, Netherlands; 192 Center for Medical Biometry and Medical Informatics, University of Freiburg, Freiburg, Germany; 193 Health Statistics Group, University of Exeter Medical School, Exeter, UK; 194 University of Exeter Medical School, Exeter, UK; 195 University of Dundee, Dundee, UK; 196 Cochrane Netherlands, Julius Center for Health Sciences and Primary Care, University Medical Center, Utrecht, The Netherlands; 197 Julius Center for Health Sciences and Primary Care, University Medical Center, Utrecht, The Netherlands; 198 Centre for Statistics in Medicine, NDORMS, Botnar Research Centre, University of Oxford, Oxford, UK; 199 University of Exeter, Exeter, UK; 200 University of Dundee, Dundee, UK; 201 Institute of Quality and Efficiency in Health Care, Cologne, Germany; 202 Health Services Research Unit, University of Aberdeen, Aberdeen, UK; 203 Centre for Experimental Medicine, Queen’s University Belfast, Belfast, UK; 204 Nuffield Department of Orthopaedics, Rheumatology and Musculoskeletal Sciences, University of Oxford, Oxford, UK; 205 Robertson Centre for Biostatistics, University of Glasgow, Glasgow, UK; 206 School of Medicine, University of St Andrews, St Andrews, UK; 207 Department of Ophthalmology, NHS Grampian, Aberdeen, UK; 208 Vision & Eye Research Unit, Anglia Ruskin University, Cambridge, UK; 209 The National Institute for Health Research (NIHR) Diagnostic Evidence Co-operative (DEC) London, London, UK; 210 Surgery & Cancer, Imperial College Healthcare NHS Trust, London, UK; 211 Imperial Clinical Trials Unit (ICTU), School of Public Health, Imperial College London, London, UK; 212 The National Institute for Health Research (NIHR) Diagnostic Evidence Co-operative (DEC), London, UK; 213 Surgery & Cancer, Imperial College Healthcare NHS Trust, London, UK; 214 Institute of Global Health Innovation (IGHI), Imperial College London, London, UK; 215 Imperial Clinical Trials Unit (ICTU), School of Public Health, Imperial College London, London, UK; 216 Department of Computer Science, Brunel University London, London, UK; 217 The National Institute for Health Research (NIHR) Health Protection Research Unit in Healthcare Associated Infections and antimicrobial resistance at Imperial College, London, UK; 218 The National Institute for Health Research (NIHR) Diagnostic Evidence Co-operative (DEC) London, London, UK; 219 Public Health England, England, UK; 220 Imperial College Healthcare NHS Trust, London, UK; 221 The National Institute for Health Research (NIHR) Diagnostic Evidence Co-operative (DEC) London, London, UK; 222 Surgery & Cancer, Imperial College Healthcare NHS Trust, London, UK; 223 Department of Clinical Biochemistry, The Christie Pathology Partnership, The Christie NHS Foundation Trust, Manchester, UK; 224 School of Medicine, University of Notre Dame, Notre Dame, Australia; 225 National Health and Medical Research Council (NHMRC) Clinical Trials Centre, University of Sydney, Camperdown, Australia; 226 ARC Consulting, Perth, Australia; 227 The Norwegian Quality Improvement of Primary Care Laboratories (NOKLUS), Haraldsplass Deaconess Hospital, Bergen, Norway; 228 Department of Public Health and Primary Health Care, University of Bergen, Bergen, Norway; 229 Laboratory of Clinical Biochemistry, Haukeland University Hospital, Bergen, Norway; 230 Department of Clinical Chemistry and Laboratory Medicine, Leiden University Medical Center, Leiden, The Netherlands; 231 Abbott Diagnostics, Wiesbaden, Germany; 232 Medical and Scientific Affairs, Roche Diagnostics International Ltd, Rotkreuz, Switzerland; 233 Medical and Scientific Affairs, Roche Diagnostics GmbH, Penzberg, Germany; 234 Department of Clinical Epidemiology, Biostatistics & Bioinformatics, Academic Medical Center, University of Amsterdam, Amsterdam, The Netherlands; 235 SEALS Department of Clinical Chemistry and Endocrinology, Prince of Wales Hospital and School of Medical Sciences, University of New South Wales, Sydney, Australia; 236 Screening and Test Evaluation Program, School of Public Health, University of Sydney, Camperdown, Australia; 237 Julius Center for Health Sciences and Primary Care, University Medical Center Utrecht, 3584 CG Utrecht, Netherlands; 238 Exeter Test Group, University of Exeter Medical School, Exeter, UK; 239 Exeter Test Group, University of Exeter Medical School, Exeter, UK; 240 Exeter Test Group, University of Exeter Medical School, Exeter, UK; 241 Statistics Group, NIHR CLAHRC South West Peninsula (PenCLAHRC), University of Exeter Medical School, Exeter, UK; 242 Lady Davis Institute for Medical Research, Jewish General Hospital, Montréal, Québec Canada; 243 Department of Epidemiology, Biostatistics and Occupational Health, McGill University, Montréal, Québec Canada; 244 Department of Medicine, McGill University, Montréal, Québec Canada; 245 Respiratory Epidemiology and Clinical Research Unit, McGill University Health Centre, Montréal, Québec Canada; 246 Department of Medicine, Department of Health Research and Policy, Department of Statistics, Stanford University, Stanford, California USA; 247 Department of Clinical, Neuro and Developmental Psychology, Vrije Universiteit (VU) University, Amsterdam, The Netherlands; 248 Hull York Medical School and the Department of Health Sciences, University of York, Heslington, York UK; 249 Libraries, Concordia University, Montréal, Québec Canada; 250 Departments of Community Health Sciences and Psychiatry, University of Calgary, Calgary, Alberta Canada; 251 Department of Mathematics and Statistics, McGill University, Montréal, Québec Canada; 252 Department of Medicine, Johns Hopkins University School of Medicine, Baltimore, Maryland USA; 253 Department of Rehabilitation Medicine, University of Washington, Seattle, Washington USA; 254 Department of Neuroscience and Behavior, Faculty of Medicine of Ribeirão Preto, University of São Paulo, Ribeirão Preto, Brazil; 255 Department of Psychiatry and Behavioral Sciences, University of Washington, Seattle, Washington USA; 256 Department of Family Medicine and Community Health, University of Minnesota, Minneapolis, Minnesota USA; 257 Department of Psychiatry, EMGO Institute, VU University Medical Center, Amsterdam, The Netherlands; 258 Department of Psychiatry, Faculty of Medicine, Ramathibodi Hospital, Mahidol University, Bangkok, Thailand; 259 Department of Psychosomatic Medicine and Psychotherapy, University Medical Center Hamburg-Eppendorf and Schön Klinik Hamburg Eilbek, Hamburg, Germany; 260 Department of Family Medicine, School of Medical Sciences, Universiti Sains Malaysia, Kelantan, Malaysia; 261 Centre for Women’s Mental Health, Royal Women’s Hospital, Parkville, Victoria Australia; 262 Department of General Practice, Academic Medical Center, University of Amsterdam, Amsterdam, The Netherlands; 263 Department of Veterans Affairs Medical Center, San Francisco, California USA; 264 Richard L Roudebush Veteran Affairs Medical Center, Health Services Research and Development, Indiana University School of Medicine, Indianapolis, Indiana USA; 265 Department of Psychiatry and Department of General Practice, Academic Medical Center, University of Amsterdam, Amsterdam, The Netherlands; 266 Depression Clinical and Research Program, Massachusetts General Hospital, Boston, Massachusetts USA; 267 Departments of Psychiatry, Educational and Counselling Psychology, and Psychology, and School of Nursing, McGill University, Montréal, Québec Canada; 268 Exeter Test Group, University of Exeter Medical School, Exeter, UK; 269 PenTAG, University of Exeter Medical School, Exeter, UK; 270 Health Economics Research Centre, University of Oxford, Oxford, UK; 271 Department of Surgery, University of Birmingham, Birmingham, UK; 272 Birmingham Clinical Trials Unit, Institute of Applied Health Research, University of Birmingham, Birmingham, UK; 273 Department of Surgery, Heart of England Foundation Trust, Birmingham, UK; 274 Institute of Applied Health Research, University of Birmingham, Birmingham, UK; 275 Institute of Applied Health Research, University of Birmingham, Birmingham, UK; 276 University of Birmingham, Birmingham, UK; 277 Keele University, Keele, UK; 278 The Institute of Cancer Research, London, UK

**Keywords:** Faecal Immunochemical Test, Chronic Obstructive Pulmonary Disorder, Circulate Tumour Cell Count, Apply Health Research, Faecal Immunochemical Test Result

## O1 User testing of Test-Treatment Pathway derivation to help formulating focused diagnostic questions

### Gowri Gopalakrishna^1^, Miranda Langendam^1^, Rob Scholten^2^, Patrick Bossuyt^1^, Mariska Leeflang^1^

#### ^1^Department of Clinical Epidemiology, Biostatistics & Bioinformatics, Academic Medical Center, University of Amsterdam, Amsterdam, The Netherlands; ^2^Cochrane Netherlands, Julius Center for Health Sciences and Primary Care, University Medical Center Utrecht, Utrecht, Netherlands

##### **Correspondence:** Mariska Leeflang (m.m.leeflang@amc.uva.nl)

**Background:** The Test-Treatment Pathway has been proposed as a method to link test accuracy to downstream outcomes. By describing the clinical actions before and after testing, it illustrates how a test is positioned in the pathway, relative to other tests and diagnostics, and how the introduction of a new test may change the current diagnostics pathway. However, there is limited practical guidance on how to model such Test-Treatment Pathways.

**Methods:** We selected the Patient - Index test- Comparator - Outcome (PICO) format, as also used elsewhere in evidence-based medicine, as a starting point for building the Test-Treatment Pathways. From there we developed a structured set of triggering questions. We defined these questions based on several brainstorm sessions and iteratively made changes to this basic structure after three rounds of user testing. During the user testing meetings, a pathway was drawn for each specific application. All sessions were recorded both on audio and video.

**Results:** We present examples of four different Test-Treatment Pathways. User testing revealed that all users found the process of drawing the pathway very useful, but they also felt that this is just the first step in a process. The steps from pathway derivation to key questions remains difficult. Challenges in deriving the pathway were that interviewee(s) may wander off topic and that some problems cannot be captured in only one pathway. Further training was deemed desirable. Users would also like to see an electronic tool. They had no clear preference when offered a choice between a more open interviewing approach versus a more closed checklist approach.

**Discussion:** Modelling Test-Treatment pathways is a useful step in synthesizing the evidence about medical tests and developing recommendations about them, but further technical development and training are needed to facilitate their use in evidence-based medicine.

## O2 Using machine learning and crowdsourcing for the identification of diagnostic test accuracy

### Anna Noel-Storr^1^, James Thomas^2^, Iain Marshall^3^, Byron Wallace^4^

#### ^1^Cochrane Dementia and Cognitive Improvement Group, University of Oxford, Oxford, UK; ^2^EPPI-Centre, Department of Social Science, University College London, London, UK; ^3^Division of Health and Social Care Research, King’s College London, London, UK; ^4^College of Computer and Information Science, Northeastern University, Boston, USA

##### **Correspondence:** Anna Noel-Storr (anna.noel-storr@rdm.ox.ac.uk)

Identifying studies of diagnostic test accuracy (DTA) is challenging. Poor reporting and inconsistent indexing hampers retrieval, and the lack of validated filters means that sensitive searches often yield tens of thousands of results which require further manual assessment. Machine learning (ML) and crowdsourcing have shown to be highly effective at identifying reports of randomized trials, with Cochrane’s Embase screening project accurately identifying over 20,000 reports using a crowd model. Additionally, the project generated a large data set that could be used to train ML systems. The new workflow for RCT identification will combine automated and human screening to optimize system efficiency.

**Aims and objectives**

This study set out to evaluate the application of these two innovative approaches to DTA identification.

**Methods**

A gold standard data set (n = 1120) was created, composed of known DTA studies and realistic non-DTA reports. This data set was made available to both machine and crowd. Two ML strategies were evaluated: 1. An ‘active learning’ simulation, in which the abstracts presented for manual assessment were prioritized as a function of their predicted probability of relevance; 2. A binary classifier, which was evaluated via cross-validation. Outcomes of interest were machine and crowd recall and precision.

**Results**

At the time of writing, the experiments are ongoing. The active learning approach achieved 95% recall at a cost of 30% being manually screened, increasing to 100% after 77% screened. The binary classifier retrieved DTA articles with 95% recall, and 40% precision; 100% recall was possible, but with an associated precision of 13%.

**Discussion**

The gold standard used for this study was small but had the advantage of not being generated through the relative recall method. If the crowd can do this successfully, as has been shown in the case of Cochrane’s Embase project, then we will be in a position to create a vast human-generated gold standard dataset (across all relevant healthcare areas) that can be used to further improve machine learning accuracy.

This work could also be used to inform methodological filter development and refinement.

## O3 Developing plain language summaries for diagnostic test accuracy (DTA) reviews

### Penny Whiting^1^, Clare Davenport^2^, Mariska Leeflang^3^, Gowri GopalaKrishna^3^, Isabel de Salis^4^

#### ^1^University Hospitals Bristol NHS Foundation Trust, School of Social and Community Medicine, Bristol, UK; ^2^Institute of Applied Health Research, University of Birmingham, Birmingham, UK; ^3^Department of Clinical Epidemiology, Biostatistics & Bioinformatics, Academic Medical Center, University of Amsterdam, Amsterdam, The Netherlands; ^4^School of Social and Community Medicine, University of Bristol, Bristol, UK

##### **Correspondence:** Penny Whiting (Penny.whiting@bristol.ac.uk)

A plain language summary (PLS) is a stand-alone summary of a Cochrane systematic review and should provide rapid access to its content. A clear PLS is essential to ensure that systematic reviews are useful to users who are not familiar with the more technical content of the review. Explaining the results of a Diagnostic Test Accuracy (DTA) review in plain language is challenging. The review methodology and results are less familiar than reviews of interventions and the two dimensional nature of the measure of a test’s accuracy (sensitivity and specificity) introduces further complexity. Additionally, DTA reviews are characterized by a large degree of heterogeneity in results across studies. The reason for this variation is not always clear and explaining this to readers, especially lay readers, is difficult. A further challenge is providing information about the downstream consequences of testing. Challenges in the interpretation of DTA reviews may be different for different target user groups, but this is something that has yet to be established. Ideally, a PLS should be accessible to all potential target audiences (patients, clinicians, policy makers).

**Fig. 1 (abstract O3).**
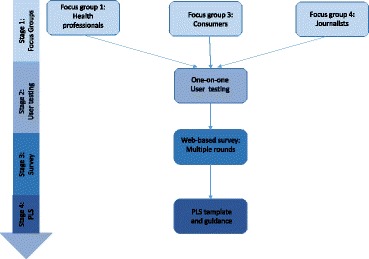
See text for description

The overall aim of this project is to develop a template and guidance for PLS for Cochrane DTA reviews. We are using a four staged approach to develop this: qualitative focus groups, one-on-one user testing, web-based survey, and producing a template and guidance for PLS for DTA reviews based on the findings from the first three stages (Fig. 1). This presentation will provide a summary of the results from the focus groups, user testing and first rounds of the web-based survey. We will present the current version of the proposed PLS based on an example review of the IQCODE for diagnosing dementia. We will then invite the audience to provide feedback on various aspects of the proposed example PLS using interactive turning point voting software. Feedback from the presentation will then be incorporated into the next version of the PLS.

## O4 Prediction model study risk of bias assessment tool (PROBAST)

### Sue Mallett^1^, Robert Wolff^2^, Penny Whiting^3^, Richard Riley^4^, Marie Westwood^2^, Jos Kleinen^2^, Gary Collins^5^, Hans Reitsma^6,7^, Karel Moons^6^

#### ^1^Institute of Applied Health Research, University of Birmingham, Birmingham, UK; ^2^Kleijnen Systematic Reviews Ltd, York, UK; ^3^University Hospitals Bristol NHS Foundation Trust, School of Social and Community Medicine, Bristol, UK; ^4^Research Institute for Primary Care and Health Sciences, Keele University, Keele, UK; ^5^Centre for Statistics in Medicine, Nuffield Department of Orthopaedics, Rheumatology and Musculoskeletal Sciences, University of Oxford, Oxford, UK; ^6^Julius Center for Health Sciences and Primary Care, University Medical Center Utrecht, Utrecht, The Netherlands; ^7^Cochrane Netherlands, University Medical Center Utrecht, Utrecht, The Netherlands

##### **Correspondence:** Sue Mallett (s.mallett@bham.ac.uk)

**Background**: Quality assessment of included studies is a crucial step in any systematic review. Review and synthesis of prediction modelling studies is a relatively new and evolving area and a tool facilitating quality assessment for prognostic and diagnostic prediction modelling studies is needed.

**Objectives**: To introduce PROBAST, a tool for assessing the risk of bias and applicability of prediction modelling studies.

**Methods**: A Delphi process, involving 42 experts in the field of prediction research, was used until agreement on the content of the final tool. Existing initiatives in the field of prediction research such as the REMARK (Reporting Recommendations for Tumor Marker Prognostic Studies) guidelines and the TRIPOD prediction model reporting guidelines formed part of the evidence base for the tool development. The scope of PROBAST was determined with consideration of existing tools, such as QUIPS and QUADAS.

**Results**: After seven rounds of the Delphi procedure, a final tool has been developed which utilises a domain-based structure supported by signalling questions similar to QUADAS-2, which assesses risk of bias and applicability of diagnostic accuracy studies. PROBAST assesses the risk of bias and applicability of prediction modelling studies. Risk of bias refers to the likelihood that a prediction model leads to distorted predictive performance for its intended use and targeted individuals. The predictive performance is typically evaluated using calibration, discrimination, and (re)classification. Applicability refers to the extent to which the prediction model from the primary study matches your systematic review question, for example in terms of the population or outcomes of interest.

PROBAST comprises five domains (participant selection, outcome, predictors, sample size and flow, and analysis) and 24 signalling questions grouped within these domains.

**Conclusions**: PROBAST can be used for the quality assessment of prediction modelling studies. The presentation will give an overview of the development process and the final version of the tool (including the addressed domains and signalling questions).

## O5 Nonparametric meta-analysis for diagnostic accuracy studies

### Antonia Zapf^1^, Annika Hoyer^2^, Katharina Kramer^1^, Oliver Kuss^2^

#### ^1^Department of Medical Statistics, University Medical Center Göttingen, Göttingen, Germany; ^2^Institute for Biometry and Epidemiology, German Diabetes Center, Leibniz Institute for Diabetes Research at Heinrich Heine University, Düsseldorf, Germany

##### **Correspondence:** Antonia Zapf (Antonia.Zapf@med.uni-goettingen.de)

**Background**

Summarizing the information of many studies using a meta-analysis becomes more and more important, also in the field of diagnostic studies. The special challenge in meta-analysis of diagnostic accuracy studies is that in general sensitivity and specificity are co-primary endpoints. Across the studies, both endpoints are correlated, and this correlation has to be considered in the analysis.

**Methods**

The standard approach for such a meta-analysis is the bivariate logistic random effects model. An alternative, more flexible approach is to use marginal beta-binomial distributions for the true positives and the true negatives, linked by copula distributions. However, both approaches can lead to convergence problems. We developed a new, nonparametric approach of analysis, which has greater flexibility with respect to the correlation structure. Furthermore, the nonparametric approach avoids convergence problems.

**Results**

In a simulation study, it became apparent that the empirical coverage of all three approaches is in general below the nominal level. Regarding bias, empirical coverage, and mean squared error the nonparametric model is often superior to the standard model, and comparable with the copula model. I will also show the application of the three approaches for two example meta-analyses: one with very high specificities and low variability, and one with an outlier study.

**Conclusion**

In summary, the nonparametric model as compared with the standard model and the copula model has better or comparable statistical properties, no restrictions on the correlations structure and always converges. Subject of further research is the consideration of multiple thresholds per study.

**Reference**

[1] Zapf A, Hoyer A, Kramer K, Kuss O (2015). Nonparametric meta‐analysis for diagnostic accuracy studies. Statistics in Medicine, 34(29):3831–41.

## O6 Meta-analysis of test accuracy studies using imputation for partial reporting of multiple thresholds

### J. Ensor^1^, J. J. Deeks^2^, E. C. Martin^3^, R. D. Riley^1^

#### ^1^Research Institute for Primary Care and Health Sciences, Keele University, Keele, UK; ^2^Institute of Applied Health Research, University of Birmingham, Birmingham, UK; ^3^Manchester Pharmacy School, University of Manchester, Manchester, UK

##### **Correspondence:** J. Ensor (j.ensor@keele.ac.uk)

**Introduction**: For continuous tests, primary studies usually report test accuracy results at multiple thresholds, but the set of thresholds used often differs. This creates missing data when performing a meta-analysis at each threshold. A standard meta-analysis (NI: No Imputation) ignores such missing data. A Single Imputation (SI) approach was recently proposed to recover missing threshold results using a simple piecewise linear interpolation. Here, we propose a new method (MIDC) that performs Multiple Imputation of the missing threshold results using Discrete Combinations, and compare the approaches via simulation.

**Methods**: The new MIDC method imputes missing threshold results (two by two tables) by randomly selecting from the set of all possible discrete combinations which lie between the results for two known bounding thresholds. Imputed and observed results are then synthesised in a bivariate meta-analysis at each threshold separately. This is repeated M times, and the M pooled results at each threshold are combined using Rubin’s rules to give final estimates.

**Results**: Compared to the standard NI approach, our simulations suggest both SI and MIDC approaches give more precise pooled sensitivity and specificity estimates, due to the increase in data. Coverage of 95% confidence intervals was also closer to 95%, with the MIDC method generally performing best, especially when the prevalence was low. This is primarily due to improved estimation of the between-study variances. In situations where the linearity assumption was valid in logit ROC space, and there was selective reporting of thresholds, the imputation methods also reduced bias in the summary ROC curve.

**Conclusions**: The MIDC method is a new option for dealing with missing threshold results in meta-analysis of test accuracy studies, and generally performs better than the current method in terms of coverage, precision and, in some situations, bias. A real example will be used to illustrate the method.

## O7 Modelling multiple biomarker thresholds in meta-analysis of diagnostic test accuracy studies

### Gerta Rücker^1^, Susanne Steinhauser^2^, Martin Schumacher^1^

#### ^1^Institute for Medical Biometry and Statistics, Faculty of Medicine and Medical Center – University of Freiburg, Stefan-Meier-Str. 26, 79104 Freiburg, Germany; ^2^Institute of Medical Statistics, Informatics and Epidemiology, University of Cologne, Kerpener Str. 62, 50937 Cologne, Germany

##### **Correspondence:** Gerta Rücker (ruecker@imbi.uni-freiburg.de)

**Background**

In meta-analyses of diagnostic test accuracy, routinely only one pair of sensitivity and specificity per study is used. However, for tests based on a biomarker often more than one threshold and the corresponding values of sensitivity and specificity are known.

**Methods**

We present a new meta-analysis approach using this additional information. It is based on the idea of estimating the distribution functions of the underlying biomarker within the non-diseased and diseased individuals. Assuming a normal or logistic distribution, we estimate the distribution parameters in both groups applying a linear mixed effects model to the transformed data. The model accounts for both the within-study dependence of sensitivity and specificity and between-study heterogeneity.

**Results**

We obtain a summary receiver operating characteristic (SROC) curve as well as the pooled sensitivity and specificity at every specific threshold. Furthermore, the determination of an optimal threshold across studies is possible through maximization of the Youden index. The approach is demonstrated on a meta-analysis on the accuracy of Fractional Exhaled Nitric Oxide (FENO) for diagnosing asthma.

**Conclusion**

Our approach uses all the available information and results in an estimation not only of the performance of the biomarker but also of the threshold at which the optimal performance can be expected.

## O8 Summarising and validating test accuracy results across multiple studies for use in clinical practice

### Richard Riley^1^, Joie Ensor^1^, Kym Snell^2^, Brian Willis^3^, Thomas Debray^4^, Karel Moons^5^, Jon Deeks^3^, Gary Collins^6^

#### ^1^Research Institute for Primary Care and Health Sciences, Keele University, Keele, UK; ^2^Keele University, Keele, UK; ^3^Institute of Applied Health Research, University of Birmingham, Birmingham, UK; ^4^University of Utrecht, Utrecht, Netherlands; ^5^Julius Center for Health Sciences and Primary Care, University Medical Center Utrecht, 3584 CG Utrecht, Netherlands; ^6^Centre for Statistics in Medicine, Nuffield Department of Orthopaedics, Rheumatology and Musculoskeletal Sciences, University of Oxford, Oxford, UK

##### **Correspondence:** Richard Riley (r.riley@keele.ac.uk)

Following a meta-analysis of test accuracy studies, the translation of summary results into clinical practice is potentially problematic. The sensitivity, specificity, and positive (PPV) and negative (NPV) predictive values of a test may differ substantially from the average meta-analysis findings, due to heterogeneity. Clinicians thus need more guidance: given the meta-analysis, is a test likely to be useful in new populations and, if so, how should test results inform the probability of existing disease (for a diagnostic test) or future adverse outcome (for a prognostic test)? In this presentation, we propose ways to address this [1].

Firstly, following a meta-analysis we suggest deriving prediction intervals and probability statements about the potential accuracy of a test in a new population. Secondly, we suggest strategies for how clinicians should derive post-test probabilities (PPV and NPV) in a new population based on existing meta-analysis results, and propose a cross-validation approach for examining and comparing their calibration performance. Application is made to two clinical examples. In the first, the joint probability that both sensitivity and specificity will be > 80% in a new population is just 0.19, due to a low sensitivity. However, the summary PPV of 0.97 is high and calibrates well in new populations, with a probability of 0.78 that the true PPV will be at least 0.95. In the second example, post-test probabilities calibrate better when tailored to the prevalence in the new population, with cross-validation revealing a probability of 0.97 that the observed NPV will be within 10% of the predicted NPV. We recommend that meta-analysts should go beyond presenting just summary sensitivity and specificity results, by also evaluating and, if necessary, tailoring their meta-analysis results for clinical practice [2].

We conclude with brief extension to the risk prediction modelling field, where similar issues occur: in particular, the distribution of model performance (e.g. in terms of calibration, discrimination and net-benefit) should be evaluated across multiple settings, as focusing only on summary performance can mask serious deficiencies [3].

**References**

1. Riley RD, Ahmed I, Debray TP, et al. Summarising and validating test accuracy results across multiple studies for use in clinical practice. Stat Med 2015; 34: 2081–2103.

2. Willis BH, Hyde CJ. Estimating a test’s accuracy using tailored meta-analysis: How setting-specific data may aid study selection. J Clin Epidemiol 2014; 67: 538–546.

3. Snell KI, Hua H, Debray TP, Ensor J, Look MP, Moons KG, Riley RD. Multivariate meta-analysis of individual participant data helped externally validate the performance and implementation of a prediction model. J Clin Epidemiol 2016; 69: 40–50

## O9 Barriers to blinding: an analysis of the feasibility of blinding in test-treatment RCTs

### Lavinia Ferrante di Ruffano^1^, Brian Willis^1^, Clare Davenport^1^, Sue Mallett^1^, Sian Taylor-Phillips^2^, Chris Hyde^3^, Jon Deeks^1^

#### ^1^Institute of Applied Health Research, University of Birmingham, Birmingham, UK; ^2^Division of Health Sciences, Warwick Medical School, The University of Warwick, Coventry, UK; ^3^Exeter Test Group, University of Exeter Medical School, Exeter, UK

##### **Correspondence:** Lavinia Ferrante di Ruffano (ferrantl@bham.ac.uk)

**Background**: Test-treatment strategies are complex interventions involving four main ingredients: 1) testing, 2) diagnostic decision–making, 3) therapeutic decision–making, 4) subsequent treatment. Methodologists have argued that it may be impossible to control for performance bias when evaluating these strategies using RCTs, since test results must be used by clinicians to plan patient management whilst patients are often actively involved in testing processes and treatment selection. Analysis of complex therapeutic interventions has shown blinding is not always feasible, however claims regarding the ability to blind in test-treatment trials have not been evaluated.

**Aim**: This methodological review analysed a systematically–derived cohort of 103 test-treatment trials to determine the frequency of blinding, and feasibility of blinding care–providers, patients and outcome assessors.

**Methods**: Judgments of feasibility were based on subjective assessments following previously published methods1. Extraction and judgements were completed in duplicate, with final judgement decisions made as a group consensus consisting of methodologists and clinicians.

**Fig. 2 (abstract O9).**
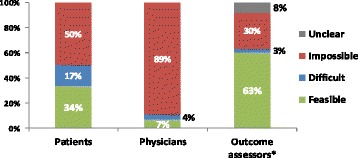
The feasibility of blinding patients, care-providers and outcome assessors in test-treatment RCTs

**Provisional results**: Care–providers, patients and outcome assessors were masked by 4%, 5% and 22% of trials, and could have been masked by a total of 11%, 50% and 66% respectively (Fig. 2). Scarcity of attempts to blind reflected the practical and ethical difficulties in performing sham diagnostic procedures, or masking real test results from patients and clinicians. Feasibility hinged on: the types of tests, nature of their comparison, type of information produced and circumstances surrounding their administration.

**Conclusions**: These findings present worrying implications for the validity of test-treatment RCTs. Unexpectedly we found that in some circumstances blinding may alter or eliminate the desired test–treat effect, and recommend further investigation to determine the true impact of masking in these highly complicated trials.

**Reference**

1. Following method of Boutron et al. J Clin Epidemiol 2004;57:543–550

## O10 Measuring the impact of diagnostic tests on patient management decisions within three clinical trials

### Sue Mallett^1^, Stuart A. Taylor^2^, Gauraang Batnagar^2^, STREAMLINE COLON Investigators^2^, STREAMLINE LUNG Investigators^2^, METRIC Investigators^2^

#### ^1^Institute of Applied Health Research, University of Birmingham, Birmingham, UK; ^2^Centre for Medical Imaging, University College London, London, UK

##### **Correspondence:** Sue Mallett (s.mallett@bham.ac.uk)

**Background**: Standard studies comparing diagnostic tests measure diagnostic test accuracy. Some trials also provide information on additional outcomes such as time to diagnosis and differences between the number of additional tests in patient pathway. Ideally diagnostic tests would be compared as interventions in randomised controlled trials (RCTs). However RCTs for comparison of diagnostic tests as interventions can be problematic to design and run. Problems include long time periods required for studies following patient outcomes during which either test or treatment pathways change, high numbers of patients required, high costs, ethical issues about randomising to receive tests, difficulty to understand role of diagnostic test as complex intervention, plus other barriers. However for some tests it may be possible to measure how tests affect patient management decisions within current diagnostic accuracy trials.

**Aims**: To describe three ongoing clinical trials measuring the impact of diagnostic tests on patient management.

**Methods:** Three trials, each comparing alternative diagnostic tests or diagnostic test pathways against a reference standard of normal clinical practice have been designed to collect patient management decisions. In each patient management decisions based on the alternative pathways are reported based on eight or ten alternative management options. STREAMLINE COLON and LUNG compare whole body MRI to current NICE recommended pathways for detection of metastases at diagnosis of colon and lung cancer respectively. METRIC compares ultrasound and MRI for diagnosing the extent and activity of Crohn’s disease in newly diagnosed and relapsed patients.

**Discussion of bias and applicability**: Including patient management decision into diagnostic accuracy studies increases understanding when comparing the role of diagnostic tests. Including patient management decision making can be onerous to collect in terms of clinical and trialist time. Reduction of bias through blinding of test results and patient management decisions between test pathways being compared, may only be achieved when patient management decisions are made outside of normal clinical pathways. However the most applicable decisions of patient management will be made by normal treating clinicians within normal clinical pathways, when blinding of both test results to each other is less feasible. Constraints of timing and personnel mean trialists may be choosing between trial designs at risk of bias with high applicability or at low risk of bias but high risk of clinical applicability. More methodology work on including patient management decisions based on diagnostic tests is required to understand best ways to design studies and to understand robustness and realism of different methods.

## O11 Comparison of international evidence review processes for evaluating changes to the newborn blood spot test

### Sian Taylor-Phillips^1^, Lavinia Ferrante Di Ruffano^2^, Farah Seedat^3^, Aileen Clarke^3^, Jon Deeks^2^

#### ^1^Division of Health Sciences, Warwick Medical School, The University of Warwick, Coventry, UK; ^2^Institute of Applied Health Research, University of Birmingham, Birmingham, UK; ^3^University of Warwick, Coventry, UK

##### **Correspondence:** Sian Taylor-Phillips (s.taylor-phillips@warwick.ac.uk)

**Background**

Newborn blood spot screening involves taking a spot of blood from a baby’s heel in the first 7 days of life, and testing for a range of rare disorders using Tandem Mass Spectrometry. It is not possible to conduct randomised controlled trials of screening for these rare diseases, so decisions about which disorders to include must be made in the absence of such evidence. In this study we evaluated how the evidence is used to make national policy decisions about which diseases to include in the newborn blood spot test.

**Methods**

In the absence of RCT evidence, the evidence can be linked together to understand probable patient outcomes. We developed a framework of pathways to patient outcomes building on the work of Raffle and Gray, Harris et al., and Adriaensen et al. in screening, and di Ruffano et al. in test evaluation. We systematically reviewed the literature to identify national screening decision making organisations, their criteria and processes of decision making, and all policy and review documents related to the Newborn blood spot test with no time limits. For each country we analysed how the evidence for each patient pathway and outcome had been considered in practice.

**Results**

There was large variation between countries, the median number of disorders included in the newborn blood spot test was 19, ranging from 5 in Finland to 54 in the US. Methods of deciding which disorders to include involved expert panel consensus without formal evidence review (Netherlands), systematic review with meta-analysis and economic modelling (UK), and using recommendations and reviews from other countries (Italy). Key elements of pathways to patient outcomes included test accuracy, treatment benefit of early detection, and overdiagnosis. While 8/15 countries considered potential overdiagnosis in at least one review, only 1/15 (the UK) attempted to quantify the numbers overdiagnosed, and this used a comparison of prevalence between countries with and without screening which is subject to significant bias. Complete results by country by disease for pathways to patient outcomes covered, evidence review methods, and association between these and policy decisions will be available in time for the conference.

## O12 Reviewing the quantity and quality of evidence available to inform NICE diagnostic guidance. Initial results focusing on end-to-end studies

### Sarah Byron^1^, Frances Nixon^1^, Rebecca Albrow^1^, Thomas Walker^1^, Carla Deakin^1^, Chris Hyde^2^, Zhivko Zhelev^2^, Harriet Hunt^2^, Lavinia Ferrante di Ruffano^3^

#### ^1^National Institute for Health and Care Excellence, Diagnostic Assessment Programme, Manchester, UK; ^2^Exeter Test Group, University of Exeter Medical School, Exeter, UK; ^3^Institute of Applied Health Research, University of Birmingham, Birmingham, UK

##### **Correspondence:** Chris Hyde (c.j.hyde@exeter.ac.uk)

**Background**

NICE has been producing guidance on medical diagnostic technologies since 2011. This has so far resulted in 24 pieces of guidance on wide-ranging topics. As part of the process of reviewing its methods, the pieces of guidance and the underpinning evidence are being examined to inform thinking on potential future developments. The expectation in diagnostics assessments is that end-to-end (E2E) studies, directly linking test use to patient outcome, such as comparative outcome studies like RCTs, are rarely available and so there will be a greater reliance on economic modelling as the main tool to assess whether the diagnostic technology is effective and cost-effective. This study reports findings on the availability, nature and impact of any E2E studies informing the guidance so far.

**Objectives**

- To identify how many pieces of NICE diagnostics guidance were informed by E2E studies
- Where E2E studies were found, to describe their nature
- To describe how the E2E studies informed committee discussions and the final guidance

**Methods**

The approach was a document analysis of all pieces of published diagnostics guidance and the underpinning evidence. A data extraction form was developed and piloted on one of the pieces of diagnostics guidance and its underpinning evidence. Extraction was performed by one researcher and checked by a second. Data was tabulated and conclusions derived from the tables produced.

**Main results**

Although identifiable, the number of E2E studies could often not be quickly located in either the under-pinning reports or the guidance. 11/24, 46% (95% CI 26, 66) of guidance had any E2E studies, but in three of these the numbers were very small. The E2E studies were mostly RCTs. Where the test in the guidance was used for diagnosis, there was a mean of 1.9 E2E studies and where used for monitoring there was a mean of 12.2 E2E studies. The difference was unlikely to have occurred by chance alone (Wilcoxon Mann-Whitney Test U = 20 p < 0.05). In the guidance where there were substantial numbers of RCTs, clear account was taken of them as evidenced by the amount of space devoted to them in the “Outcomes” and “Considerations” sections

**Authors’ conclusions**

End-to-end studies are already an important part of the evidence base in the assessment of diagnostic technologies. HTA methods need to anticipate the likely continuing growth of these study types.

## O13 Use of decision modelling in economic evaluations of diagnostic tests: an appraisal of Health Technology Assessments in the UK since 2009

### Yaling Yang^1^, Lucy Abel^1^, James Buchanan^1^, Thomas Fanshawe^1^, Bethany Shinkins^2^

#### ^1^Nuffield Department of Primary Health Care Sciences, University of Oxford, Oxford, UK; ^2^Academic Unit of Health Economics, Leeds Institute of Health Sciences, University of Leeds, Leeds, UK

##### **Correspondence:** Yaling Yang (yaling.yang@phc.ox.ac.uk)

**Background**: Diagnostic tests play an important role in the clinical decision-making process by providing information that enables patients to be stratified to the most appropriate treatment and management strategies. Timely and accurate diagnosis is therefore crucial for improving patient outcomes. By synthesising evidence from multiple sources, decision analytic modelling can be used to evaluate the cost-effectiveness of diagnostic tests in a comprehensive and transparent way.

**Objectives**: This study critically assesses the methods currently used to model the cost-effectiveness of diagnostic tests in Health Technology Assessment (HTA) reports published in the UK, and highlights areas in need of methodological development.

**Methods**: HTA reports published from 2009 onwards were screened to identify those reporting an economic evaluation of a diagnostic test using decision modelling. Existing decision modelling checklists were identified in the literature and reviewed. Based on this review a modified checklist was developed and piloted. This checklist covered 11 domains of good practice criteria, including:

- whether the decision problem is clearly defined and the analytical perspective specified
- whether the comparators are appropriate given the scope
- whether the model structure is justified and reflects the natural progress of the condition and available treatment options
- whether the inputs are consistent with the stated perspective
- whether sources of parameter values are systematically identified, clearly referenced, and appropriately synthesised
- whether model assumptions are discussed
- whether appropriate sensitivity analyses are performed

A scoring system was then applied, with marks of ‘0, 0.5 and 1’ indicating that criteria were ‘not met, partially met, and met’, respectively. The results were analysed and summarised to demonstrate to what extent the HTA reports meet the quality criteria, and identify any outstanding challenges.

**Results and conclusions**: A total of 484 HTA reports have been published since 2009, of which 38 met the inclusion criteria. The reports covered a variety of conditions including cancers, chronic diseases, acute diseases and mental health conditions. The diagnostic tests included lab-based, genetic and point-of-care tests, imaging, clinical risk prediction scores, and quality of life measures. In general, the models were of high quality with a clearly defined decision problem and analytical perspective. The model structure was usually consistent with the health condition and care pathway. However, the inherent complexity of the models was rarely handled appropriately: limited justification was provided for selection of comparators and few models fully accounted for uncertainty in treatment effects. The analysis is ongoing and full results will be presented in the paper.

## O14 Clinical utility of prediction models for ovarian tumor diagnosis: a decision curve analysis

### Laure Wynants^1,2^, Jan Verbakel^3^, Sabine Van Huffel^1,2^, Dirk Timmerman^4^, Ben Van Calster^4,5^

#### ^1^KU Leuven Department of Electrical Engineering (ESAT), STADIUS Center for Dynamical Systems, Signal Processing and Data Analytics, Leuven, Belgium; ^2^KU Leuven iMinds Department Medical Information Technologies, Leuven, Belgium; ^3^University of Oxford, Nuffield Department of Primarcy Care Health Sciences, Oxford, UK; ^4^KU Leuven Department of Development and Regeneration, Leuven, Belgium; ^5^Department of Public Health, Erasmus MC, Rotterdam, Netherlands

##### **Correspondence:** Laure Wynants (laure.wynants@esat.kuleuven.be)

**Purpose**: To evaluate the clinical utility of prediction models to diagnose ovarian tumors as benign versus malignant using decision curves.

**Methods**: We evaluated the widely used RMI scoring system using a cut-off of 200, and the following risk models: ROMA and three models from the International Ovarian Tumour Analysis (IOTA) consortium (LR2, SRrisks, and ADNEX). We used a multicenter dataset of 2403 patients collected by IOTA between 2009 and 2012 to compare RMI, LR2, SRrisks, and ADNEX. Additionally, we used a dataset of 360 patients collected between 2005 and 2009 at the KU Leuven to compare RMI, ROMA, and LR2. The clinical utility was examined in all patients, as well as in several relevant subgroups (pre- versus postmenopausal, oncology versus non-oncology centers).

We quantified clinical utility through the Net Benefit (NB). NB corrects the number of true positives for the number of false positives using a harm-to-benefit ratio. This ratio is the odds of the risk of malignancy threshold at which one would suggest treatment for ovarian cancer (e.g. surgery by an experienced gynaecological oncologist). A threshold of 20% (odds 1:4) implies that up to 4 false positives are accepted per true positive. Using NB, a model can be compared to competing models or to default strategies of treating all or treating none. We expressed the difference between models as gain in ‘net specificity (i.e., sensitivity for a constant specificity, ΔNB/prevalence). 95% confidence intervals were obtained by bootstrapping.

**Fig. 3 (abstract O14).**
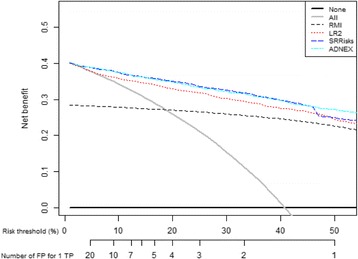
See text for description

**Results**: Thresholds between 5% (odds 1:19) and 50% (odds 1:1) were considered reasonable. RMI performed worst and was harmful, i.e. worse than treat all, at thresholds <20%. ADNEX and SRrisks consistently showed best performance (see Fig. 3). At the 10% threshold, SRrisks’ net sensitivity was 4% (95% CI 3% to 6%) higher than that of LR2, but similar to the net sensitivity of ADNEX (difference 0%, 95% CI -1% to 1%). Subgroup results showed similar patterns. On the second dataset, results for RMI were similar. In addition, LR2 performed best for the entire range of thresholds, and was the only model with clinical utility at a risk threshold of 10%.

**Conclusions**: NB supersedes discrimination and calibration to quantify the clinical utility of prediction models. Our data suggest superior utility of IOTA models compared to RMI and ROMA.

## O15 Adjusting for indirectness in comparative test accuracy meta-analyses

### Mariska Leeflang, Aeliko Zwinderman, Patrick Bossuyt

#### Department of Clinical Epidemiology, Biostatistics & Bioinformatics, Academic Medical Center, University of Amsterdam, Amsterdam, The Netherlands

##### **Correspondence:** Mariska Leeflang (m.m.leeflang@amc.uva.nl)

**Background**: The accuracy of a diagnostic test should be compared to the accuracy of its alternatives. Direct comparisons of tests, in the same patients and against the same reference standard, offer the most valid study design, but are not always available. Comparative systematic reviews are therefore bound to rely on indirect comparisons. As the results from these comparisons can be biased, we investigated ways to correct for indirectness.

**Methods**: From a large systematic review about the accuracy of D-Dimer testing for venous thromboembolism, we selected those comparisons between two assays that contained three or more direct comparisons and four or more indirect comparisons or single assay studies. Each comparison was analyzed using the bivariate random effects meta-regression model with assay-type, directness and interaction between the two as covariates in the model. In comparisons with a significant effect of the interaction term on sensitivity or specificity, we included the following study features to correct for these differences: referral filter, consecutive enrolment, time-interval, one or more reference standards, verification and year of publication.

**Results**: Seventeen comparisons were eligible for our analyses. In nine of these, the direct comparisons showed a significant difference between test A and test B while the indirect comparisons did not; or vice versa. However, the interaction term between assay and indirectness showed a significant (P < 0.05) effect on logit-sensitivity and/or logit-specificity in only four of them. Addition of study features as covariates removed the significant effect of the interaction term in two meta-analyses. In the first one, the interaction term was significant for sensitivity (P = 0.006), but after addition of the covariate ‘time-interval between index test and reference standard’ and after addition of the covariate ‘year of publication’, the P-value became 0.086 and 0.096 respectively. In the other analysis, the interaction term was significant for specificity (P = 0.039), but after addition of the covariate ‘all results verified’ and after addition of the covariate ‘only one reference standard used’, the P-value became 0.160 and 0.083 respectively.

**Conclusions**: Adjusting the effect of directness for study features seems to be possible in some instances, but no systematic effects were found. Study characteristics that may be influential in one comparison, may have no influence at all in another comparisons.

## O16 Estimates of excess cancer incidence and cancer deaths avoided in Great Britain from 1980 -2012: the potential for overdiagnosis

### Jason Oke, Jack O’Sullivan, Rafael Perera, Brian Nicholson

#### Nuffield Department of Primary Care Health Sciences, University of Oxford, Oxford, UK

##### **Correspondence:** Jason Oke (Jason.oke@phc.ox.ac.uk)

**Introduction**: Overdiagnosis is often described as the detection of disease that will not progress to cause symptoms or premature death. No consensus exists on the most appropriate method to estimate overdiagnosis. At a population level it can be estimated using incidence and mortality data with sufficient length of observation to account for lead time. We examined incidence and mortality patterns over the last 30 years for the most common cancers in Great Britain, with the aim of developing a method to identify potential overdiagnosis.

**Methods**: Mortality data were available since 1950 while incidence data were obtained from 1979. We used log-linear regression to model the long-term trend in age-standardised cancer-specific mortality rates for the “pre-diagnostic era” (1950–78) and used these results to predict both mortality and incidence rate in the “diagnostic era” (1980–2012). We used current (“diagnostic era”) incidence and mortality data from Cancer Research UK to calculate excess incidence and deaths avoided by subtracting the observed rates from the predicted rates in ten cancers types for men and women separately. We used the ratio of excess incidence to deaths avoided to summarise our findings.

**Results**: Simple straight-line models accounted for between 50 and 92% of variation seen in mortality rates in the pre-diagnostic era. Mortality in the diagnostic era closely followed the predicted trends except for breast cancer. In contrast, observed incidence was generally greater by several orders of magnitude to that predicted by the model. Cumulative excess incidence ranged from between 16 cases per 100,000 for thyroid cancer to 1763 per 100,000 for cervical cancer. The model estimated the number of cumulative deaths avoided as zero for the following cancers: oral (both men and women), prostate (men), bowel (men) and kidney (women). For the cancers where the ratio of excess incidence to deaths avoided could be estimated, these ratios varied from 1:1 (non-Hodgkin’s Lymphoma (NHL) in women) to 107:1 (Uterine in women).

**Conclusions**: The use of long-term mortality data may be useful for identifying and quantifying overdiagnosis by ecological analysis. Our results show that the incidence of many of the most common cancers in Great Britain has increased significantly in the last three decades but this has not necessarily prevented cancer deaths. We suggest that much of the increased detection represents the overdiagnosis of cancer.

## O17 Identifying the utility and disutility associated with the over-diagnosis of early breast cancers for use in the economic evaluation of breast screening programmes

### Hannah L. Bromley^1^, Tracy E. Roberts^1^, Adele Francis^2^, Denniis Petrie^3^, G. Bruce Mann^4^

#### ^1^Department of Health Economics, University of Birmingham, Birmingham, UK; ^2^Department of Breast Surgery, Nuffield House, University Hospital Birmingham, Birmingham, UK; ^3^Melbourne School of Population and Global Health, University of Melbourne, Parkville, Australia; ^4^Department of Breast Surgery, Royal Women’s Hospital, Melbourne, Australia

##### **Correspondence:** Tracy E Roberts (T.E.ROBERTS@bham.ac.uk)

**Background**: Misplaced policy decisions about screening programmes may exist unless the decision process explicitly accommodates the disutility of screening and treating individuals subject to over-diagnosis. In breast cancer screening, radical surgery or radiotherapy for a woman with an over-diagnosed result would impose a serious unnecessary harm on that woman. At the individual level the harm may not actually be realised because the woman may never know that she had her breast removed unnecessarily. However, at a societal level these collective harms, if quantified, could be included in the analysis and might serve to outweigh the benefits of the screening programme. Recent evidence suggests the benefits of screening programmes have been overstated but the extent and duration of the loss of quality of life as a result of over-diagnosis has been under-researched.

**Objectives**: To explore the hypothesis that the explicit inclusion of potential disutility associated with the treatment of over-diagnosed early breast cancers will change the relative cost-effectiveness of the current recommended breast screening strategy.

**Methods**: Preliminary literature searches have shown that although multiple utility health states exist for early and metastatic breast cancers, there is significant heterogeneity between values and limited research on quantifying values associated with the breast screening programme itself. Little has been done to address the problem of over-diagnosis in breast cancer screening or attempts made to quantify associated losses in quality of life. A systematic review of utility and disutility values associated with breast screening is carried out to inform the design of a pilot study devised to capture the disutilities associated with over-diagnosis in screening mammography.

**Results**: The review results and protocol for the primary work will be completed by June. It is anticipated that women will report a loss in utility associated with the screening process, in particular with false positive mammograms, but limited numbers of such studies may render pooling of these states problematic. Very few economic evaluations of screening mammography explicitly include over-diagnosis in their analysis.

**Discussion**: This study highlights the challenges of estimating and incorporating the disutility of over-diagnosis in evaluations of screening programmes. The results from the review and the pilot study will be incorporated into a model based economic evaluation of the breast screening programme to estimate losses in quality of life associated with unnecessary treatment as a result of over-diagnosis.

## O18 Systematic review of frameworks for staged evaluation of predictive biomarkers

### Kinga Malottki^1^, Holly Smith^1^, Jon Deeks^2^, Lucinda Billingham^1^

#### ^1^Cancer Research UK Clinical Trials Unit (CRCTU), Institute of Cancer and Genomic Sciences, University of Birmingham, Birmingham, UK; ^2^Institute of Applied Health Research, University of Birmingham, Birmingham, UK

##### **Correspondence:** Kinga Malottki (k.malottki@bham.ac.uk)

**Background**: Stratified medicine has been defined as using predictive biomarkers to identify cohorts of patients more likely to benefit from a treatment (or less likely to experience a serious adverse event). There are numerous successful predictive biomarkers that have changed clinical practice. There are also examples where potential predictive biomarkers failed at a late stage of development (ERCC1 expression – platinum-based chemotherapy), or there is uncertainty about their utility in spite of being introduced into clinical practice (EGFR expression - erlotinib). These examples, together with the need to optimise the use of resources by prioritising research activities, suggest a structured approach to biomarker development may be necessary. There is a well-established model for phased evaluation of drugs, however no such model is in place for predictive biomarkers. There have been various publications on this topic both by research groups and institutions (such as the FDA). However there is no accepted model and it remains unclear whether there is consensus in the literature on the best approach to staged evaluation of predictive biomarkers.

**Aim**: To identify existing frameworks for staged evaluation of predictive biomarkers and the stages these propose. For the identified stages, to explore the outcomes, relevant study designs and requirements for the entry into and completion of each stage. To compare and contrast the different frameworks and therefore identify requirements for development of a predictive biomarker.

**Methods**: We have undertaken a systematic review of papers suggesting a framework for staged evaluation of predictive biomarkers. These were identified through broad searches of MEDLINE, EMBASE and additional internet searches. The identified frameworks were compared and grouped based on the context in which the development of a predictive biomarker was discussed (for example development of a biomarker predicting treatment safety) and the stages proposed.

**Findings**: We identified 22 papers describing a framework for staged evaluation of predictive biomarkers. These were grouped into four models: (1) general predictive biomarker development, (2) integrated into phased drug development, (3) development of a multi-marker classifier and (4) development of marker predicting treatment safety. It appeared that the most complete model was (1) general, which comprised stages: pre-discovery, discovery, analytical validation, clinical validation, clinical utility, implementation. The remaining models mostly contained stages corresponding to these, however models (2) and (3) did not contain analytical validation and model (4) clinical validation. The stages in models (2-4) corresponding to those in model (1) were occasionally merged or divided into multiple stages. Different terminology was also used to describe similar concepts. Relevant study designs were described for all stages, however there seemed to be consensus mainly for the clinical utility stage, where generally RCTs designed to evaluate the biomarker were suggested (including enrichment, stratified and biomarker-strategy designs).

**Conclusions**: The identified models suggest the need to consider the context in which the biomarker is developed. There was a large overlap between the four models, suggesting consensus on at least some of the research steps that may be necessary prior to predictive biomarker implementation into clinical practice.

## O20 Biological variability studies: design, analysis and reporting

### Alice Sitch, Sue Mallett, Jon Deeks

#### Institute of Applied Health Research, University of Birmingham, UK

##### **Correspondence:** Alice Sitch (a.j.sitch@bham.ac.uk)

**Introduction**: To use a test to the best effect when monitoring disease progression or recurrence of patients it is vital to have accurate information regarding the variability of the test, including sources and estimation of measurement error. There are many sources of variability when testing a population. There is variability in the results for a single patient even when in a stable disease state, this fluctuation in results is within-individual variability, and there is also variability in results from person-to-person which is known as between-individual variability. When undertaking a test, there is pre-analytical variability which occurs before the test is analysed, including within-individual and between-individual variations e.g. timing of measurement. Analytical variation is the variability in test results during the process of obtaining the result, such as when a sample is assayed in a laboratory test. Where interpretation of tests can be subjective, there are intra-inter reader studies to assess variability, comparing interpretations from multiple observers.

**Objectives**: To review the current state of variability studies for tests to identify best methods and where studies could be improved. Our research focusses on design, sample size, methods of analysis and quality of studies.

**Methods**: To understand the scope of studies evaluating biological variability, the design, methods for analysis, reporting and overall quality, a review of studies of biological variation was conducted and, whilst conducting this review, the key methodological papers influencing these studies were identified. The searches used to identify papers to be included in the review were: key word search (bio* AND vari*) for the period 1st November 2013 to 31st October 2014; all articles published in the journals Clinical Biochemistry, Radiology and Clinical Chemistry during the period 1st January 2014 to 31st December 2014; papers included in the Westgard QC database published from 1st January 2000 onwards; and, detailed searches for three different test types (imaging, laboratory and physiological) in specific clinical areas: ultrasound imaging to assess bladder wall thickness in patients with incontinence; creatinine and Cystatin C measurements to estimate glomerular filtration rate (GFR) in patients with chronic kidney disease (CKD); and, spirometry to measure forced expiratory volume (FEV) in patients with chronic obstructive pulmonary disorder (COPD). In addition to the papers identified by these searches, published articles identified by previous and concurrent work meeting the criteria were included to enrich the sample.

Key information regarding the design, analysis and results reported was extracted from each paper. In addition, analyses of data from a biological variability study were conducted to demonstrate the current framework for design and analysis and investigate the impact of various components within this.

**Results**: The review identified 106 studies for assessment allowing the current state of the field with regard to design, analysis and reporting to be evaluated. We will present our findings on typical designs including examples of patient recruitment, sample sizes, analysis methods and sources of variability addressed.

**Conclusions**: This work will identify the current state of the methodology in this area to help identify where future work to improve the design, analysis and reporting of biological variability studies is needed.

## O21 Intra- and interrater agreement with quantitative positron emission tomography measures using variance component analysis

### Oke Gerke^1^, Mie Holm-Vilstrup^1^, Eivind Antonsen Segtnan^1^, Ulrich Halekoh^2^, Poul Flemming Høilund-Carlsen^3^

#### ^1^Department of Nuclear Medicine, Odense University Hospital, Odense, Denmark; ^2^Epidemiology, Biostatistics and Biodemography, University of Southern Denmark, Odense, Denmark; ^3^Department of Nuclear Medicine, Odense University Hospital & Department of Clinical Research, University of Southern Denmark, Odense, Denmark

##### **Correspondence:** Oke Gerke (oke.gerke@rsyd.dk)

**Purpose**: Any quantitative measurement procedure needs to be both accurate and reliable in order to justify its use in clinical practice. Reliability concerns the ability of a test to distinguish patients from each other, despite measurement errors, and is usually assessed by intraclass correlation coefficients (ICC). Agreement, on the other hand, focuses on the measurement error itself, and various parameters are used in practice, comprising proportions of agreement, standard errors of measurement, coefficients of variations, and Bland-Altman plots. We revive variance component analysis (VCA) in order to decompose the observed variance attributable to different sources of variation (e.g. rater, scanner, time point), to derive relevant repeatability coefficients (RC), and to show the connection to Bland-Altman plots in a test-retest setting. Moreover, we propose a sequential sample size strategy when assuming differences in a test-retest setting to follow approximately a Normal distribution.

**Methods**: Variants of the commonly used standard uptake value (SUV) in cancer imaging from two studies at our institution were used. In study 1, thirty patients were scanned once pre-operatively for the assessment of ovarian cancer. These 30 images were assessed two times by the same rater two months apart. In study 2, fourteen patients with a confirmed diagnosis of glioma were scanned up to 5 times before and during treatment, and the resulting 50 images were assessed by three raters. Studies 1 and 2 served as examples for intra- and interrater variability assessment, respectively. In study 1, we treated ‘reading’ (1st vs. 2nd) as fixed factor and ‘patient’ as random factor. In study 2, both ‘rater’ and ‘time point’ were considered fixed effects, whereas ‘patient’ and ‘scanner’ were treated as random effects. The sequential sample size strategy, post hoc applied to data from study 1, was based on a hypothesis test on the population variance of the differences between measurements, assuming that the differences follow a Normal distribution. An overall recruitment plan of 15 + 15 + 20 patients was assumed, and the adjustment for multiple testing was done by applying a α-spending function according to Kim, DeMets (Biometrika 1987).

**Results**: In study 1, the within-subject standard deviation times 2.77 resulted in a RC of 2.46, which is equal to the half width of the Bland-Altman band. The RC is the limit within which 95% of differences will lie. In Study 2, the RC for identical conditions (same patient, same rater, same time point, same scanner) was 2392, allowing for different scanners resulted in a RC of 2543. The differences between raters were, though, negligible compared to the other factors: estimated difference between reader 1 and 2: -10, 95% CI: -352 to 332; reader 1 vs. 3: 28, 95% CI: -313 to 370. The adjusted significance levels for the tests conducted with 15 and 30 patients, respectively, were 0.015 and 0.03. Investigating a range of hypothetical population variance values of 0.25, 0.5, …, 4 resulted in rejecting the one-sided null for 3 at both stages.

**Conclusion**: VCA seems to be an obvious, yet intriguing approach to agreement studies which often are tackled with simple measures only. VCA is, indeed, built upon various model assumptions, but that had been neither obstacle to the wide use of ICCs in reliability analysis. The ice is getting even thinner when basing the sample size strategy on population variance tests (and the normality assumption), which, though, could be applied in an adaptive manner in the same way as it is often done in therapeutic trials.

## O22 Robust novel tolerance intervals and correlated-errors-in-variables regressions for the equivalence and validation of new clinical measurement methods

### Bernard G. Francq (bernard.francq@glasgow.ac.uk)

#### Robertson Centre for Biostatistics, University of Glasgow, Glasgow, UK

The need of laboratories to quickly assess the quality of samples leads to the development of new methods, and improvement of existing methods. It is hoped that these will be more accurate than the reference method. To be validated, these alternative methods should lead to results comparable (equivalent) with those obtained by a standard method.

Two main methodologies for assessing equivalence in method-comparison studies are presented in the literature. The first one is the well-known and widely applied Bland–Altman approach with its agreement intervals, where two methods are considered interchangeable if their differences are not clinically significant. The second approach is based on errors-in-variables regression in a classical (X,Y) plot and focuses on confidence intervals, whereby two methods are considered equivalent when providing similar measures notwithstanding the random measurement errors. This research reconciles these two methodologies and shows their similarities and differences using both real data and simulations. New consistent correlated-errors-in-variables regressions are introduced as the errors are shown to be correlated in the Bland–Altman plot. Indeed, the coverage probabilities collapse and the biases soar when this correlation is ignored. Robust novel tolerance intervals are compared with agreement intervals, and novel predictive intervals are introduced with excellent coverage probabilities.

We conclude that the (correlated)-errors-in-variables regressions should not be avoided in method comparison studies, although the Bland–Altman approach is usually applied to avert their complexity. We argue that tolerance or predictive intervals are better than agreement intervals. It will be shown that tolerance intervals are easier to calculate and easier to interpret. Guidelines for practitioners regarding method comparison studies will be discussed.

**Reference**

1. Francq, B. G., and Govaerts, B. (2016) How to regress and predict in a Bland–Altman plot? Review and contribution based on tolerance intervals and correlated-errors-in-variables models. Statist. Med., doi: 10.1002/sim.6872

## O23 Validation of using early modelling to predict the performance of a monitoring test – the use of the ELF biomarker in liver disease modelling and the ELUCIDATE trial

### Jon Deeks^1^, Alice Sitch^1^, Jac Dinnes^1^, Julie Parkes^2^, Walter Gregory^3^, Jenny Hewison^3^, Doug Altman^4^, William Rosenberg^5^, Peter Selby^3^

#### ^1^Institute of Applied Health Research, University of Birmingham, Birmingham, UK; ^2^University of Southampton, Southampton, UK; ^3^University of Leeds, Leeds, UK; ^4^Centre for Statistics in Medicine, Nuffield Department of Orthopaedics, Rheumatology and Musculoskeletal Sciences, University of Oxford, Oxford, UK; ^5^University College London, London, UK

##### **Correspondence:** Jon Deeks (j.deeks@bham.ac.uk)

**Background**: Monitoring tests can be used to identify disease recurrence or progression. Monitoring strategies are complex interventions, involving specification of a test, a schedule, a threshold or decision rule based on test results, and subsequent diagnostic or therapeutic action. Before undertaking an RCT of a monitoring strategy all four of these components of the monitoring intervention need to be defined. Excess false positives and potentially unnecessary interventions can be caused by using a poorly discriminating or imprecise test, monitoring too frequently, or choosing to act at too low a threshold.

**Aim**: The study focused on evaluating monitoring strategies using the ELF test to detect progression of fibrosis to decompensated cirrhosis in patients with severe liver disease. The study had two aims: (1) To use early modelling to predict performance of the ELF test for different monitoring schedules and thresholds; (2) To validate the modelling by comparison with results from an RCT of the monitoring strategy.

**Methods**: A simulation model was constructed using evidence from the literature, existing data sources and expert opinion to inform disease progression, relationship of the marker with the disease state, and measurement error in the marker. The test schedule and decision rule were varied to identify optimal strategies. The ELUCIDATE RCT randomized 878 patients to an ELF based monitoring strategy or usual care, and was undertaken at the same time as the modelling. Data from the RCT are now available on process of care outcomes, disease based outcomes will be available in the future. Comparisons are made between predictions from the simulation model and results of the trial.

**Results**: Identifying data to build the simulation model was challenging, particularly concerning test characteristics. The simulation model demonstrated that the performance of the monitoring strategy was most influenced by estimates of disease progression, measurement error of the test and the test threshold. The test strategy as used in the ELUCIDATE trial was predicted to lead to high rates of early intervention, which was then observed in the trial in terms of numbers of patients being referred for further investigation in the monitoring arm than in usual care.

**Conclusion**: Early modelling of monitoring strategies is recommended prior to undertaking RCTs of monitoring strategies to assist in determining optimal test thresholds and frequencies. The study highlights the importance of obtaining valid data on both the performance of the test and the progression of disease before planning trials.

## O24 Diagnostic accuracy in the presence of an imperfect reference standard: challenges in evaluating latent class models specifications (a Campylobacter infection case)

### Julien Asselineau^1^, Paul Perez^1^, Aïssatou Paye^1^, Emilie Bessede^2^, Cécile Proust-Lima^3^

#### ^1^Bordeaux University Hospital, Public Health Department, Clinical Epidemiology Unit and CIC 1401 EC, Bordeaux, France; ^2^French National Reference Center for Campylobacter and Helicobacter, Bordeaux, France; ^3^INSERM U1219, Bordeaux Population Health Research Center, Bordeaux, France; Univ. Bordeaux, ISPED, Bordeaux, France

##### **Correspondence:** Julien Asselineau (julien.asselineau@isped.u-bordeaux2.fr)

**Introduction**: Usual methods to estimate diagnostic accuracy of index tests in the presence of an imperfect reference standard result in biased accuracy and prevalence estimates. The latent class model (LCM) methodology deals with imperfect reference standard by statistically defining the true disease status and possibly assuming residual dependences between diagnostic tests conditionally on this status. Different dependence specifications can lead to inconsistent accuracy estimates, therefore thorough evaluation of models should be systematically undertook although this is rarely done in practice. We use the study of new campylobacter detection tests in which bacteriological culture is an imperfect reference standard to illustrate the complexity of the implementation of a LCM methodology to assess the diagnostic accuracy of detection tests.

**Methods**: Five tests of campylobacter infection (bacteriological culture, one molecular test and three immunoenzymatic tests) were applied to stool samples of 623 symptomatic patients at Bordeaux and Lyon University Hospital in 2009. Their sensitivity and specificity were estimated with LCMs using maximum likelihood method after probit or logit transformations. Conditional independence hypothesis between tests was relaxed by specifying alternative dependence structures based on random effects. Performances of the models were compared using information criteria, goodness-of-fit statistics with asymptotic or empirical distributions (to tackle many rare or missing profiles) and bivariate residual statistics. Two main functions implementing LCMs were used: NLMIXED procedure in SAS® and randomLCA package in R.

**Results**: Among the 25 = 32 theoretical profiles of test responses, 17 were observed including 10 with 3 patients or more. The model under conditional independence hypothesis presented the worst Akaike information criterion (AIC) and was highly rejected by all statistics. Introducing a random effect common to all diagnostic tests improved the AIC but the model was still rejected by nearly all statistics. Among the other dependence structures evaluated, the model assuming a residual dependence between the three immunoenzymatic tests showed the best AIC. Statistics using empirical distributions were just above the significance level (p > 0.05) except for the total bivariate residual statistics (p = 0.03). With this model, prevalence of campylobacter infection was 0.11. As expected, culture presented the lowest sensitivity (82.1% vs 85.2%–98.5% for other tests) and the highest specificity (99.6% vs 95.8%–98.4% for other tests). When evaluated by simulations, performances of NLMIXED procedure for the random effect shared by all diagnostic tests showed low coverage rates while randomLCA package provided correct inferences.

**Conclusion**: LCM methodology allowed estimating the diagnostic accuracy of new campylobacter detection tests and of culture which is an imperfect reference standard as confirmed by our results. Model assessment steps are crucial to select the best specification in LCM and get valid accuracy estimates. However, their interpretation is tricky because of discordant conclusions depending on the statistics used. Rare or missing profiles are frequent when diagnostic tests present high accuracy making use of asymptotic distributions inadequate. Statistics using empirical distributions therefore need to be specifically implemented. Lastly, usual softwares can have limitations due to their unreliability (NLMIXED) or lack of flexibility (randomLCA).

## O26 Measures to reduce the impact of missing data on the reference standard data when designing diagnostic test accuracy studies

### Christiana Naaktgeboren^1^, Joris de Groot^1^, Anne Rutjes^2,3^, Patrick Bossuyt^4^, Johannes Reitsma^1^, Karel Moons^1^

#### ^1^Julius Center for Health Sciences and Primary Care, University Medical Center Utrecht, 3584 CG Utrecht, Netherlands; ^2^CTU Bern, Department of Clinical Research, University of Bern, Bern, Switzerland; ^3^Institute of Social and Preventive Medicine, University of Bern, Bern, Switzerland; ^4^Department of Clinical Epidemiology and Biostatistics, Academic Medical Center, University of Amsterdam, Amsterdam, Netherlands

##### **Correspondence:** Christiana Naaktgeboren (c.naaktgeboren@umcutrecht.nl)

Despite efforts to determine the presence or absence of the condition of interest in all participants in a diagnostic accuracy study, missing reference standard results (i.e. missing outcomes) are often inevitable and should be anticipated in any prospective diagnostic accuracy study.

Analyses that include only the participants in whom the reference standard was performed are likely to produce biased estimates of the accuracy of the index tests. Several analytical solutions for dealing with missing outcomes are available; however, these solutions require knowledge about the pattern of missing data, and they are no substitute for complete data.

In this presentation we aim to provide an overview of the different patterns of missing data on the reference standard (i.e. incidental missing data, data missing by research design, data missing due to clinical practice, data missing due to infeasibility), the recommended corresponding solutions (i.e. analytical correction methods or including a second reference standard), and the specific measures that can be taken before and during a prospective diagnostic study to enhance the validity and interpretation of these solutions. In the presentation various examples will be discussed.

Researchers should anticipate the mechanisms that generate missing reference standard results before the start of a study, so that measures and actions can explicitly be taken to reduce the potential for biased estimates of the accuracy of the tests, markers, or models under study, as well as to facilitate correction in the analysis phase. In all cases, researchers should include in their study report how missing data on the index test and reference standard were handled, as invited by the STARD reporting guideline.

## O27 Quantifying the impact of different approaches for handling continuous predictors on the performance of a prognostic model

### Gary Collins^1^, Emmanuel Ogundimu^1^, Jonathan Cook^1^, Yannick Le Manach^2^, Doug Altman^1^

#### ^1^Centre for Statistics in Medicine, Nuffield Department of Orthopaedics, Rheumatology and Musculoskeletal Sciences, University of Oxford, Oxford, UK; ^2^Departments of Anesthesia & Clinical Epidemiology and Biostatistics, Michael DeGroote School of Medicine, Faculty of Health Sciences, McMaster University and the Perioperative Research Group, Population Health Research Institute, Hamilton, Canada

##### **Correspondence:** Gary Collins (gary.collins@csm.ox.ac.uk)

**Background**: Continuous predictors are routinely encountered when developing a prognostic model. Categorising continuous measurements into two or more categories has been widely discredited. However, it is still frequently done when developing a prognostic model due to its simplicity, investigator ignorance of the potential impact and of suitable alternatives, or to facilitate model uptake.

**Methods**: A resampling study was performed to examine three broad approaches for handling continuous predictors on the performance of a prognostic model: 1. Multiple methods of categorising predictors (including dichotomizing at the median; categorising into 3, 4, 5, equal size groups; categorising age only into 5 and 10-year age groups), 2. modelling a linear relationship between the predictors and outcome, and 3. modelling a nonlinear relationship using fractional polynomials or restricted cubic splines. Using the THIN dataset, we used primary care general practice data (from England) to develop models using Cox regression to predict a) the 10-year risk of cardiovascular disease (n = 1.8 million) and b) 10-year risk of hip fracture (n = 1 million). We also examine the impact of sample in developing the prognostic models on model performance (using data sets with 25, 50, 100 and 2000 outcome events). We compare the performance (measured by the c-index, calibration and net benefit) of prognostic models built using each approach, evaluating them using separate data from Scotland.

**Results**: Our results show that categorising continuous predictors produces models with poor predictive performance (calibration and discrimination) leading to limited clinical usefulness (net benefit). A large difference of between the mean c-index produced by the approaches (as large as 0.1 for the hip fracture model) that did not categorise the continuous predictors and the approach that dichotomised the continuous predictors at the median. The calibration of the models was poor for all methods that used categorisation, which was further exacerbated when the models were developed on small sample sizes. Over a range of clinically relevant probability thresholds, an additional net 5 to 10 cardiovascular disease cases per 1,000 were found during validation if models that implemented fractional polynomials or restricted cubic splines were used, rather than models that dichotomised all of the continuous predictors at the median without conducting any unnecessary treatment. The models that used fractional polynomials or restricted cubic splines (no difference between the two nonlinear approaches), or that assumed a linear relationship between the predictor and outcome all showed a higher net benefit, over a range of thresholds, than the categorising approaches

**Conclusions**: Categorising continuous predictors is unnecessary, biologically implausible and inefficient and should not be used in prognostic model development.

## O28 Does ignoring clustering in multicenter data influence the performance of prediction models? A simulation study

### Laure Wynants^1,2^, Yvonne Vergouwe^3^, Sabine Van Huffel^1,2^, Dirk Timmerman^4^, Ben Van Calster^4,5^

#### ^1^KU Leuven Department of Electrical Engineering (ESAT), STADIUS Center for Dynamical Systems, Signal Processing and Data Analytics, Leuven, Belgium; ^2^KU Leuven iMinds Department Medical Information Technologies, Leuven, Belgium; ^3^Center for Medical Decision Sciences, Department of Public Health, Erasmus MC, Rotterdam, the Netherlands; ^4^KU Leuven Department of Development and Regeneration, Leuven, Belgium; ^5^Department of Public Health, Erasmus MC, Rotterdam, Netherlands

##### **Correspondence:** Laure Wynants (laure.wynants@esat.kuleuven.be)

**Background**: Clinical risk prediction models are increasingly being developed and validated on multicenter datasets. We investigate how the choice of modeling technique affects the predictive performance of the model, and whether this effect depends on the level of validation.

**Method**: We present a comprehensive framework for the evaluation of the predictive performance of prediction models at both the center level and the population level, considering population-averaged predictions, center-specific predictions and predictions assuming average center effects. We sampled large (100 events per variable) datasets from simulated source populations (n = 20,000, 20 centers) with strong clustering (intraclass correlation 20%). A random intercept (RI) model and a standard logistic regression (LR) model were built in each sample. The agreement between predicted and observed risks was evaluated in the remainder of the source population using the calibration slope, which ideally equals 1, the calibration intercept, which ideally equals 0, and the c-statistic.

**Results**: Predictions from the RI model assuming average center effects were well calibrated within clusters (${\widehat{\upbeta}}_{\mathrm{cal}\kern0.5em \mathrm{within}}\kern0.5em =\kern0.5em 0.98$) but too extreme at the population level (${\widehat{\upbeta}}_{\mathrm{cal}}\kern0.5em =\kern0.5em 0.88$). Center-specific predictions from the RI model were well calibrated within clusters (${\widehat{\upbeta}}_{\mathrm{cal}\kern0.5em \mathrm{within}}\kern0.5em =\kern0.5em 0.99$) and at the population level (${\widehat{\upbeta}}_{\mathrm{cal}}\kern0.5em =\kern0.5em 0.98$). Population-averaged predictions from the standard LR model were not extreme enough for within-cluster calibration (${\widehat{\upbeta}}_{\mathrm{cal}\kern0.5em \mathrm{within}}\kern0.5em =\kern0.5em 1.09$) but were well calibrated at the population level (${\widehat{\upbeta}}_{\mathrm{cal}}\kern0.5em =\kern0.5em 0.99$). We show that this pattern is explained by the well-known difference between marginal and conditional effects[1]. The same pattern was observed for the calibration intercepts, with miscalibration of the predictions assuming an average center effect at the population level and of the population-averaged predictions at the cluster level. The c-statistic at the population level was higher for center-specific predictions (C = 0.815) than for average center predictions and population-averaged predictions (Cs = 0.764). At the cluster level, all c-statistics were the same (C_within_ = 0.785).

**Conclusion**: We recommend that model development (standard vs RI model) reflects the data structure, while the level of model validation (cluster level vs population level) reflects the research question. Generally, center-specific predictions offer the best population-level and center-level calibration and discrimination. However, center-specific predictions are not available for patients from new centers. Population-averaged predictions are a good alternative when population-level calibration is required, while predictions assuming an average center effect are a good alternative when center-level calibration is required.

**Reference**

1. Zeger SL, Liang K-Y, Albert PS: Models for Longitudinal Data: A Generalized Estimating Equation Approach. Biometrics 1988, 44(4):1049–1060.

## O29 The effects of treatment use when externally validating a prediction model that did not include treatment as predictor

### Romin Pajouheshnia^1^, Rolf Groenwold^1^, Karen Moons^1^, Johannes Reitsma^1,2^, Linda Peelen^1^

#### ^1^Julius Center for Health Sciences and Primary Care, University Medical Center Utrecht, Utrecht, The Netherlands; ^2^Cochrane Netherlands, University Medical Center Utrecht, Utrecht, The Netherlands

##### **Correspondence:** Romin Pajouheshnia (R.Pajouheshnia@umcutrecht.nl)

**Background and objectives**: Prognostic models are, among other things, used to provide risk predictions for individuals who are not receiving a certain treatment, in order to assess the natural course of a disease and in turn guide treatment decisions. As treatment availability and use changes over time, researchers may be faced with assessing the validity of existing models in partially treated populations, and may account for treatment use in different ways. We aimed to investigate how treatment use contributes to the poor performance commonly observed in external validation studies, and to explore methods to address the issue.

**Methods**: The effect of treatment use on the observed performance of a model was evaluated analytically. Development data sets representing untreated individuals were simulated using a logistic model and “optimal” models were developed using those sets. Validation sets drawn from the same theoretical population were simulated to receive an effective (binary) treatment. The prevalence and effectiveness of treatment were varied, with and without being dependent on true risk. Model performance in the validation sets was expressed in terms of calibration slope, observed:expected ratio (O:E) and C statistic. We examined the results of i) ignoring treatment, ii) restricting validation to untreated patients, and iii) adjusting the observed event rates to account for treatment effects. This was expressed through the difference (Δ) between each performance measure after applying a method and the value observed in the untreated set.

**Results**: Validation of a model derived in untreated individuals in a treated validation set resulted in poorer model performance than that observed in the same population, if left untreated. Treatment of 50% of patients with a highly effective treatment (higher risk patients had a higher probability of receiving treatment; treatment effect odds ratio: 0.5), resulted in a decrease in the O:E from 1.0 to 0.7, and a decrease in the C statistic from 0.67 to 0.62 when compared to the observed statistics in an untreated set. This trend was observed across settings with different mechanisms for treatment allocation and different population risk distributions. As treatment prevalence and effectiveness increased, the observed model performance almost invariably decreased. Restricting the validation to only untreated individuals resulted in performance measures closer to those observed in the full untreated validation set, at the cost of precision (Δ O:E = 0.0; C statistic = 0.03). When treatment allocation was completely based on risk (I.e. according to a risk threshold), the restriction approach was less effective (ΔO:E = 0.0; ΔC statistic = 0.07). Increasing the observed event rates to account for treatment effects improved the observed model calibration, but this was highly sensitive to incorrect assumptions about treatment use and effectiveness.

**Conclusions**: Validating a model designed to make predictions of the “natural course” of an individual’s health in a validation data set containing treated individuals may result in an underestimation of the performance of the model in untreated individuals. Current methods are not sufficient to account for the effects of treatment, and findings from such studies should be interpreted with caution.

## O30 A calibration hierarchy for risk models: strong calibration occurs only in utopia

### Ben Van Calster^1,2^, Daan Nieboer^2^, Yvonne Vergouwe^2^, Bavo De Cock^1^, Micael J. Pencina^3^, Ewout W. Steyerberg^2^

#### ^1^KU Leuven, Department of Development and Regeneration, Leuven, Belgium; ^2^Department of Public Health, Erasmus MC, Rotterdam, Netherlands; ^3^Duke Clinical Research Institute, Duke University, Durham (NC), USA; Department of Biostatistics and Bioinformatics, Duke University, Durham, NC, USA

##### **Correspondence:** Ben Van Calster (ben.vancalster@med.kuleuven.be)

**Background and objective**. Calibrated risk models are vital for valid decision support. However, definitions and approaches to investigate calibration vary. We define a hierarchy of calibration definitions and describe implications for model development and external validation of predictions, with a specific focus on model utility and sample size.

**Study design and methods**. We present results based on simulated datasets, assuming a multivariable prediction model for a dichotomous outcome based on logistic regression.

**Results**. At the bottom of the calibration hierarchy is ‘mean calibration’ or calibration-in-the-large, which only requires that the average predicted risk corresponds to the observed event rate. Next, ‘weak calibration’ or logistic calibration requires the average prediction effects to be correct, implying a calibration slope of 1 and a calibration intercept of 0. This level is by definition achieved on the development data when standard maximum likelihood is used. ‘Moderate calibration’ refers to the common definition of calibration as “having an event rate of *R*% among patients with a predicted risk of *R*%”. This implies that the flexible calibration curve lies on the diagonal. At the top of the hierarchy is ‘strong calibration’, which requires that the event rate equals the predicted risk for every covariate pattern based on the model predictors. In fact, this implies that the model is fully correct for the validation setting. We argue that this is unrealistic: the model type (e.g. the logit link) may be incorrect, at model development the linear predictor is only asymptotically unbiased, and all nonlinear and interaction effects should be correctly modeled. We prove that moderate calibration already guarantees non-harmful decision-making. Finally, results indicate that a flexible assessment of calibration in small validation datasets is problematic. We updated the val.prob function of Harrell’s rms package for R.

**Conclusion**. Strong calibration is desirable for individualized decision support, but unrealistic and counter-productive by stimulating the development of overly complex models. Model development and external validation should focus on moderate calibration.

## P1 Risk-adjusted colorectal cancer screening using the FIT: development of a risk prediction model

### Jennifer Cooper^1^, Sian Taylor-Phillips^1^, Nick Parsons^1^, Chris Stinton^1^, Steve Smith^2^

#### ^1^Division of Health Sciences, Warwick Medical School, University of Warwick, Coventry, UK; ^2^Midlands and North West Bowel Cancer Screening Hub, Hospital of St Cross, University Hospitals Coventry and Warwickshire NHS Trust, Rugby, UK

##### **Correspondence:** Jennifer Cooper (jennifer.cooper@warwick.ac.uk)

**Background & rationale**: The National Screening Committee as of January 2016 have recommended a change from the guaiac based faecal occult blood test (FOBT) to the more accurate faecal immunochemical test (FIT) in the UK following a successful Bowel Cancer Screening Programme (BCSP) pilot study. The increased uptake and test positivity demonstrated from this pilot however will challenge current colonoscopy capacity and countries will need to set the positivity threshold accordingly. The FIT has the advantage of an adjustable positive threshold and provides a quantitative haemoglobin concentration which relates to the risk of colorectal cancer (CRC). Risk scoring systems which combine the FIT concentration with individual risk factors have been shown to improve the sensitivity of the test and the cancer detection rate compared to using the screening test alone. For instance, a study in the Netherlands combined risk factors with the FIT result and showed improved sensitivity at a similar level of specificity but no such study currently exists for the UK population.

**Research aim**: The purpose of this study is to determine whether integrating routinely available predictors from the Bowel Cancer Screening System (BCSS) with the FIT result improves test performance.

**Methods**: This will be achieved using data collected for the FIT pilot study which took place April to October 2014. There were 40,930 individuals who were invited to take part in the study from the Midlands and Southern hubs with 27,167 returning a FIT. Predictive models will be developed initially using logistic regression. The first model will use the FIT result alone as a predictor of CRC and the second will use both the FIT result and the additional predictors available on the BCSS including; age, gender, IMD (from postcode), screening history and previous results. These models will be compared using model performance measures such as calibration and discrimination and test performance will be investigated by producing ROC curves and determining whether sensitivity at set specificity is improved. The model will then be developed further using more complex statistical techniques, including neural networks, to take into account potential non-linear associations.

**Relevance, application and benefits**: A risk based approach to screening may have several benefits for both patients and for the screening programme including an increased detection of early stage cancers and their precursors as well as minimising the number of false positives and false negatives. In addition, colonoscopies are an expensive and limited resource and carry their own risks for patients. This approach would aim to refer those at higher risk for colonoscopy and place those at lower risk back into the screening pool for continued surveillance and could offer an opportunity for risk communication. This study has future implications for CRC screening in the UK as well as contributing to research in risk based screening and CRC prediction models.

## P2 A comparison of the ADO, BODE and DOSE scores for predicting respiratory hospitalisations in a primary care COPD Cohort

### Andy Dickens, Rachel Jordan, Alexandra Enocson, David Fitzmaurice, Alice Sitch, Peymane Adab

#### Institute of Applied Health Research, University of Birmingham, Birmingham, UK

##### **Correspondence:** Andy Dickens (a.p.dickens@bham.ac.uk)

**Introduction** Several multidimensional prognostic indices (PI) for COPD have been developed, mostly based on patients with moderate/severe COPD. PIs have been developed to predict a range of outcomes including mortality, hospitalisations and exacerbations. The Birmingham COPD Cohort study will examine the performance of these indices in a primary care COPD population.

**Aims & objectives** Use preliminary data to determine the predictive ability of selected PIs in relation to self-reported respiratory hospitalisations at 1 and 2 years.

**Methods** Patients were recruited from 71 general practices in the West Midlands, UK. Patients either had diagnosed COPD or were identified through case-finding. Baseline data from 668 participants were used to calculate 3 PIs (ADO, BODE, DOSE). Discrimination and calibration of the PIs was examined.

**Results** According to c statistic estimates, all models had reasonable discrimination in relation to 1-year respiratory hospitalisations (c; 95% CI: ADO 0.81; 0.75–0.88, DOSE 0.78; 0.70–0.86, BODE 0.75; 0.67–0.84) and 2-year respiratory hospitalisations (c; 95% CI: ADO 0.76; 0.69–0.83, DOSE 0.75; 0.69–0.82, BODE 0.70; 0.62–0.78).

All models had poor calibration for predicting 1-year and 2-year respiratory hospitalisations.

**Conclusions** All models performed moderately well identifying patients with respiratory hospitalisations, but were poor at predicting future events. The data suggest that ADO could be a useful tool for identifying those at higher risk of respiratory hospitalisations. The analyses will be repeated on the complete sample once data is available. Components from existing PIs will be considered alongside candidate variables from the Cohort study, to modify or develop a prognostic index that more accurately predicts events in primary care COPD patients.

**Funding** This abstract summarises independent research funded by the NIHR under its Programme Grants for Applied Research Programme (Grant Reference Number RP-PG-0109-10061). The views expressed are those of the authors and not necessarily those of the NHS, the NIHR or Department of Health. The Birmingham COPD Cohort study is part of the Birmingham Lung Improvement StudieS – BLISS.

**Fig. 4 (abstract P2).**
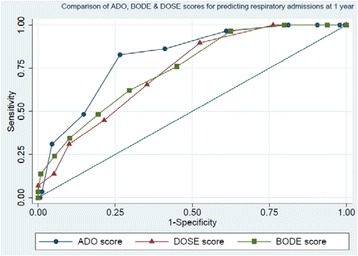
See text for description

## P3 A paradox when comparing two correlated agreement coefficients

### Bernard G. Francq^1^, Charles Boachie^1^, Gaj Vidmar^2,3,4^

#### ^1^Robertson Centre for Biostatistics, University of Glasgow, Glasgow, UK; ^2^University Rehabilitation Institute, Ljubljana, Slovenia; ^3^Faculty of Medicine, University of Ljubljana, Ljubljana, Slovenia; ^4^University of Primorska, Koper, Slovenia

##### **Correspondence:** Bernard G. Francq (bernard.francq@glasgow.ac.uk)

The kappa statistic is widely applied to assess the agreement between two clinicians rating patients on a nominal or ordinal scale. The Gwet’s coefficient has been proposed as a better alternative.

Three or more raters are often involved in such studies, e.g., 2 junior and 2 senior clinicians assessing the same patients. The question that arises is: is the agreement between the 2 junior clinicians equal to the one between the 2 senior ones. Such pairs of agreement coefficients (AC) are correlated. The confidence interval (CI) for the difference (or ratio) between 2 correlated ACs can easily be obtained by bootstrap. The closer 2 correlated ACs are, the more likely it is that the CI for their difference (ratio) contains and is ‘centred’ on 0 (1 in the case of ratio), and the larger the corresponding p-value. However, a paradox arises in practice as the CI is less likely to include 0 (1 in the case of ratio). This is due to the bootstrap distribution being truncated. This leads to the CIs being biased, having wrong limits, being too narrow and not corresponding any more to the p-values. The truncation is described and explained with real data. A simple solution is provided to tackle this issue.

The real data includes 2 trained and 2 experienced physicians assessing patients from a prosthetics and orthotics clinic with the International Classification of Functioning, Disability and Health (ICF).

## P4 Evaluation of different meta-analytic methods of outcomes of tests predicting loss of response to Infliximab in Crohn’s disease patients

### Karoline Freeman, Martin Connock, Sian Taylor-Phillips, Rachel Court, Aileen Clarke

#### Division of Health Sciences, Warwick Medical School, The University of Warwick, Coventry, UK

##### **Correspondence:** Karoline Freeman (k.freeman@warwick.ac.uk)

**Background**: Assays measuring levels of antidrug antibodies in Crohn’s disease patients treated with anti-TNF agents (e.g. Infliximab) are becoming increasingly used and may be predictive of a patient’s treatment response status possibly enabling optimisation of treatment. In most studies test results are dichotomized (+ve and –ve) and allocated to patients with loss of response (LOR) or response to treatment, thereby generating a 2 × 2 contingency table of association. The four cells of the 2 × 2 table characterise the four possible associations between the binary test and patient outcome. Published meta-analyses (MA) of such studies have not adopted a test evaluation perspective. Two differing types of MA have been undertaken: Lee et al. (2012) and Nanda et al. (2013) pooled relative risk ratios ([a/a + b]/[c/c + d]), where [a/a + b] is equivalent to PPV and [c/c + d] to “1 – NPV”, and the resulting ratio does not correspond to any commonly used measure of test accuracy; while Paul et al. (2014) pooled the odds ratio [a/b] /[ c/d] which rearranges to [a*d / b*c] and is equivalent to an estimate of the diagnostic odds ratio (DOR). Neither of these statistical approaches allow for the correlation between columns in the 2 × 2 table.

**Aim**: We aimed to compare meta-analytical outputs using the MA approaches taken by Nanda and Paul with the bivariate random effects model using studies of assays measuring antibodies to Infliximab to predict LOR in Crohn’s disease patients to document differences in MA outcomes and interpretation.

**Methods**: We used systematic review methods to search for, identify and quality assess relevant studies. We calculated unadjusted risk ratios and unadjusted odds ratios and pooled data in Stata using fixed (Mantel-Haenszel) and random effects (Der Simonian and Laird) models. We calculated sensitivity and specificity and undertook hierarchical / bivariate meta-analysis with the user-written “metandi” package of Harbord and Whiting in Stata. Sensitivity analyses were undertaken.

**Results**: We included 20 studies reporting results in 1240 patients with Crohn’s disease. Studies were considered at high risk of bias in at least one quality domain. The pooled risk ratio using fixed effects and random effects models was 2.1 (95% CI 1.8–2.4) and 2.2 (95% CI 1.6–3.2), respectively. This outcome could be interpreted as: the risk of experiencing LOR in the test positive population (a/a + b) is twice the risk of experiencing LOR in the test negative population (c/c + d). The pooled DOR was 4.6 (95% CI 2.5–8.5). This outcome could be interpreted as: the ratio of the odds of the test being positive if the patient experiences LOR relative to the odds of the test being positive if the patient is a responder is 4.6. An odds ratio of 5 would suggest a very strong association of marker and clinical outcome in traditional epidemiological studies of association but is uninformative in terms of the discriminatory power of the marker. Using the bivariate meta-analysis the pooled summary estimates of sensitivity and specificity were 0.56 (95% CI 0.44–0.67) and 0.79 (95% CI 0.69–0.87), respectively suggesting the assay to be a fair predictor of response at the most and does not render itself as a tool for treatment decisions. Sensitivity analyses showed different results for different methods.

**Conclusion**: The meaning of a pooled risk ratio is questionable and the pooled DOR can be misleading. Neither method is as useful in evaluating tests as a bivariate model.

## P5 Over-diagnosis or real patient benefit: How to evaluate new diagnostics that challenge existing disease definitions

### Joris de Groot, Christiana Naaktgeboren, Hans Reitsma, Carl Moons

#### Julius Center for Health Sciences and Primary Care, University Medical Center Utrecht, 3584 CG Utrecht, Netherlands

##### **Correspondence:** Joris de Groot (j.degroot-17@umcutrecht.nl)

A major contributor to the rising problem of overdiagnosis, with the subsequent risk of overtreatment, is the development of highly sensitive diagnostic technologies that challenge and sometimes expand prevailing disease definitions. Although the value of such new technology might be that it identifies new, milder, earlier or even other abnormalities, it is uncertain whether these “abnormalities” provide the same diagnostic and prognostic information, or require the same treatment as the original targeted disease. It is often unclear which of the newly detected abnormalities are benign and how many people might be diagnosed and treated unnecessarily as a result of widespread introduction of the new test. Failure to investigate the clinical relevance of broadening disease definitions which include these newly detected abnormalities may therefore lead to overdiagnosis and overtreatment. Spiral CT used in diagnosing pulmonary embolism, detecting small subsegmental embolisms, has been mentioned as an example of such situation.

On-going technological advancements in medicine will only further increase the development of new diagnostic technologies that challenge existing disease definitions. We show why traditional cross-sectional diagnostic accuracy studies are insufficient to evaluate such new tests and how methodology for assessing their performance should catch up and keep pace with present-day technological developments. It is crucial to improve data analysis and presentation of current diagnostic studies, to make better use of existing data, or ultimately perform test-treatment trials to answer the question whether introduction of a new high sensitive test will in fact improve patient relevant outcomes, or rather induce overdiagnosis and overtreatment.

## P6 The accuracy of near-patient haemostasis tests for predicting bleeding after cardiac surgery

### Jessica Harris^1^, Andrew Mumford^2^, Zoe Plummer^1^, Kurtis Lee^3^, Barnaby Reeves^1^, Chris Rogers^1^, Veerle Verheyden^4^, Gianni D. Angelini^4^, Gavin J. Murphy^5^

#### ^1^Clinical Trials and Evaluation Unit, University of Bristol, Bristol, UK; ^2^School of Cellular and Molecular Medicine, University of Bristol, Bristol, UK; ^3^University Hospitals Bristol NHS Foundation Trust, Bristol, UK; ^4^School of Clinical Sciences, University of Bristol, Bristol, UK; ^5^Department of Clinical Sciences, University of Leicester, Leicester, UK

##### **Correspondence:** Jessica Harris (jessica.harris@bristol.ac.uk)

**Background**: Coagulopathic bleeding is a common and severe complication of cardiac surgery. Identifying patients likely to bleed may allow earlier and more targeted therapy and reduce morbidity attributable to bleeding and transfusion. We aimed to evaluate whether the results of near-patient haemostasis tests performed before, and immediately after cardiac surgery improved the prediction of subsequent clinical concern about bleeding (CCB).

**Methods**: Eligible patients were invited to participate in a prospective cohort study to estimate the predictive value of baseline patient and procedural characteristics and 28 near-patient platelet and viscoelastometry test results obtained before and after surgery. Eligible participants were adults having a range of elective or urgent open-heart surgery procedures at the Bristol Heart Institute between March 2010 and August 2012. The primary outcome CCB is a composite of high blood loss, re-operation for bleeding that excluded surgical causes or administration of a pro-haemostatic treatment that was not part of the standard surgical care protocol. A predictive model that incorporated the baseline characteristics (such as age, sex, operative urgency and procedure) was compared with various alternative models that also included the 28 pre-operative and post-operative test results. The best predictive model was selected on the basis of the c-statistic (area under receiver operating characteristic curve).

**Findings**: CCB occurred in 449 (24.5%) of 1833 patients. A multivariable predictive model for CCB based only on baseline demographic and clinical characteristics had a c-statistic = 0.72; (95% CI 0.69 to 0.75) and correctly classified 76.8% of patients. Adding the most predictive near-patient test results before and after surgery to the baseline characteristics model improved the prediction of CCB (c-statistic = 0.75 (0.72 to 0.77)), but increased the proportion of patients correctly classified by only 0.98% (18 patients). The best predictive model incorporating both baseline characteristics and near-patient test results correctly reclassified 63 patients and incorrectly reclassified 45 patients compared to the model for baseline characteristics only. Both models were internally validated using bootstrapping and cross-validation techniques; the baseline model was also externally validated in a separate cohort of 1611 adult cardiac surgery patients (c-statistic = 0.64 (0.60 to 0.67)).

**Conclusions**: The results from existing near-patient haemostasis blood tests performed immediately before and after cardiac surgery offer little improvement in the prediction of CCB compared to baseline patient characteristics alone.

## P7 Randomised controlled trials for the evaluation of point-of-care diagnostic tests: a systematic review

### Jeremy Huddy^1^, Melody Ni^1^, Katherine Good^2^, Graham Cooke^3^, Patrick Bossuyt^4^, George Hanna^1^

#### ^1^NIHR-Diagnostic Evidence Cooperative, Imperial College London, London, UK; ^2^Department of Anaesthetics, Imperial College Healthcare NHS Trust, London, UK; ^3^Division of Infectious Diseases, Imperial College, London, UK; ^4^Department of Clinical Epidemiology, Biostatistics & Bioinformatics, Academic Medical Center, University of Amsterdam, Amsterdam, The Netherlands

##### **Correspondence:** Jeremy Huddy (j.huddy@imperial.ac.uk)

**Objective**: Randomised controlled trials (RCT) are widely regarded as the gold standard of trial design but remain rare in the evaluation of diagnostic test strategies. The use of point-of-care testing (POCT) is increasing and an understanding of the impact to clinical pathways is required by policy makers to facilitate adoption. This study aimed to investigate the endpoints used in RCTs to evaluate POCT. Secondary aims were to assess study design and quality of included studies.

**Study design and setting**: An electronic search of EMBASE and Medline was performed. Descriptive data of study design was extracted and a hybrid quality assessment tool used to score included studies.

**Results**: Eighty-four studies were included evaluating 37 POCT. Five (6%) studies investigated more than one test. The five most commonly studied test devices were coagulation (11 studies (13%)), malaria (10 studies (12%)), human immunodeficiency virus antibody (9 studies (11%)), cardiac enzyme markers (7 studies (8%)) and thromboelastography (5 studies (6%)). 76 (90%) of studies had primary endpoints that reflected patient outcomes with the most frequently observed primary endpoints being treatment efficacy and therapeutic yield that together accounted for 43 (51%) of included studies. 14 (17%) of studies investigated resource use as a primary endpoint including cost-effectiveness (11 studies (13%)), length of stay (3 studies (4%)) and frequency of doctor-patient interactions (2 studies (2%)). Forty (47%) studies were multi-centre and 18 (21%) studies were undertaken in World Bank defined developing countries. Five (6%) studies had appropriate blinding; method of randomization and sample size were not reported in 24 (29%) and 19 (23%) of studies respectively.

**Conclusion**: There remains controversy as to the importance of RCTs in diagnostics and to what degree RCT evidence contributes to successful adoption. This review highlights the scope of endpoints that can be obtained from RCTs that in the main are markers of clinical utility rather than validity and therefore may provide a better understanding of how diagnostic tests affect patient and societal outcomes.

## P8 Assessing the impact of handling missing data when validating a prognostic model

### Jie Ma, Doug Altman, Gary Collins

#### Centre for Statistics in Medicine, Nuffield Department of Orthopaedics, Rheumatology and Musculoskeletal Sciences, University of Oxford, Oxford, UK

##### **Correspondence:** Jie Ma (jie.ma@ndorms.ox.ac.uk)

**Background**: After a prognostic model has been developed it is important to evaluate its performance in an independent dataset, often referred as external validation. However, data sets used to evaluate prognostic models are frequently too small, and the handling of missing data has been shown to be poor.

**Method**: Using resampling methods with large real dataset (THIN), we investigate the impact of the missing data in the validation cohort on the evaluation of performance of the QRISK2 model for predicting the 10-year risk of developing cardiovascular disease. We also include an examination of the influence of varying the sample size. Five levels of missingness (varying from 5% to 75%) were imposed using a missing at random (MAR) mechanism, as well as varying the sample size (number of events; from 10 to 1000). Four missing data methods were applied: complete case analysis, multiple imputation using regression switching, multiple imputation using predictive mean matching and multiple imputation using flexible additive imputation models. The performance of QRISK2 was assessed by calculating measures of discrimination (c-index, D-statistic) and calibration (calibration plot). The impact of the four different approaches for handling the missing data was examined by calculating the percentage bias.

**Results**: When the amount of missing data was small, there was little difference between the various approaches for handling missing data. However, as the amount of missing data increased, multiple imputation methods provided least biased estimates and better performance than the complete case analysis. These findings were also consistent over all the sample size scenarios examined.

**Conclusion**: Our study provides insight into the impact and handling of missing data on model performance. In all scenarios, regardless of the sample size, multiple imputation outperformed complete-case analyses and should be considered when validating a prognostic model

## P9 CHecklist for critical Appraisal and data extraction in systematic Reviews of clinical prediction Modelling Studies (CHARMS)

### K. G. M. (Carl) Moons^1^, Joris A. H. de Groot^1^, Sue Mallett^2^, Doug G. Altman^3^, Johannes B. Reitsma^1,4^, Gary S. Collins^3^

#### ^1^Julius Center for Health Sciences and Primary Care, University Medical Center Utrecht, 3584 CG Utrecht, Netherlands; ^2^Institute of Applied Health Research, University of Birmingham, Birmingham, UK; ^3^Centre for Statistics in Medicine, Nuffield Department of Orthopaedics, Rheumatology and Musculoskeletal Sciences, University of Oxford, Oxford, UK; ^4^Cochrane Netherlands, University Medical Center Utrecht, Utrecht, The Netherlands

##### **Correspondence:** K. G. M. (Carl) Moons (k.g.m.moons@umcutrecht.nl)

**Background**: Publications on multivariable clinical prediction models have become abundant for both prognostic and diagnostic purposes. Systematic reviews of these studies are increasingly required to identify and critically appraise the existing evidence. There is currently no checklist or tool providing guidance for systematic reviews of studies developing or validating prediction models that can assist reviewers to define the review objectives and appraise study methodology.

**Objective**: To develop a checklist to help reviewers framing a well-defined review question, and to determine which details to extract and critically appraise from primary studies on the development or validation of multivariable diagnostic or prognostic prediction models, with a view to assessing the risk of bias and sources of heterogeneity.

**Methods**: We critically examined existing reporting guidelines and quality assessment tools, key methodological publications on clinical prediction modelling, and tools used in published systematic reviews of multivariable prediction models, to identify the relevant characteristics and domains. The checklist was tested in various systematic reviews.

**Results**: We identified 7 items important for framing the review question (diagnostic versus prognostic model, intended scope of the review, type of prediction modelling studies, target population, outcome to be predicted, time span of prediction, intended moment of using the model), and 11 domains to critically appraise the primary included studies (source of data, participants, outcome, predictors, sample size, missing data, model development, model performance, model evaluation, results, interpretation). Both were combined into the CHecklist for critical Appraisal and data extraction for systematic Reviews of prediction Modelling Studies (CHARMS).

**Conclusions**: CHARMS is designed to assist reviewers to help systematic reviewers framing their review objectives, and to determine which data to extract and critically appraise from primary studies on the development and/or validation of (diagnostic and prognostic) prediction models.

## P10 New Guideline for the Reporting of Studies Developing, Validating, or Updating a Prediction Model: the TRIPOD Statement

### Karel G. M. Moons^1^, Douglas G. Altman^2^, Johannes B. Reitsma^1,3^, Gary S. Collins^2^

#### ^1^Julius Center for Health Sciences and Primary Care, University Medical Center Utrecht, 3584 CG Utrecht, Netherlands; ^2^Centre for Statistics in Medicine, Nuffield Department of Orthopaedics, Rheumatology and Musculoskeletal Sciences, University of Oxford, Oxford, UK; ^3^Cochrane Netherlands, University Medical Center Utrecht, Utrecht, The Netherlands

##### **Correspondence:** Karel G. M. Moons (k.g.m.moons@umcutrecht.nl)

**Background and objective**. Patients and care providers are confronted with making numerous decisions based on a probability; a probability that a specific disease or condition is present (diagnostic setting) or a specific event or outcome will occur in the future (prognostic setting). To guide practitioners and patients in these probability estimations, so-called multivariable prediction models are developed. Prediction models convert 2 or more pieces of information, i.e. predictors, from the participant - e.g., an individual’s age, gender, symptoms, signs, laboratory and imaging test results - into a diagnostic or prognostic probability. Prediction models are becoming increasingly abundant. In virtually all medical domains, prediction models are being developed, evaluated (validated), extended and implemented. For some specific diseases, there are even an overwhelming number of competing prediction models for the same outcome or target population. It is therefore important that these clinical prediction models and the research done to develop, evaluate or extend these models be transparently reported. However, the overwhelming evidence shows that the quality of reporting of prediction model studies is poor. Only with full and clear reporting of information on all aspects of a prediction model can risk of bias and potential usefulness of prediction models be adequately assessed.

**Methods and results**. The Transparent Reporting of a multivariable prediction model for Individual Prognosis Or Diagnosis (TRIPOD) initiative, which has included numerous clinicians, statisticians, epidemiologists and journal editors, has produced a guideline for the reporting of studies developing, validating or updating a prediction model, whether for diagnostic or prognostic purposes. The TRIPOD Statement is a checklist of 22 items, deemed essential for transparent reporting of any prediction model study, and addresses model development, model validation and model extension studies, regardless of the study methods used. The TRIPOD Statement is accompanied by an Explanation and Elaboration article that describes the rationale for the checklist, clarifies the meaning of each item, and discusses why transparent reporting is important, with a view to assessing risk of bias and clinical usefulness of a prediction model. Each item is explained in detail and accompanied by published examples of good reporting. The document also provides a valuable reference of issues to consider when designing, conducting, and analyzing prediction model studies.

**Conclusions**. The endorsement and use this checklist by researchers and medical journal editors will help ensure that medical research findings are complete and accurately reported, understood by readers, and ultimately used by medical practitioners.

## P11 Incorporating the time-dependency in ROC methodology for censored clinical event

### Adina Najwa Kamarudin, Ruwanthi Kolamunnage-Dona, Trevor Cox

#### Department of Biostatistics, University of Liverpool, Liverpool, UK

##### **Correspondence:** Adina Najwa Kamarudin (adinajwa@liv.ac.uk)

The classical approach of ROC (receiver operating characteristic) analysis considers event (disease) status and biomarker of an individual as fixed over time; however in practice both the disease status and biomarker change over time. Individuals who are disease-free earlier may develop the disease later due to longer study follow-up, and also have their biomarker changed from baseline over follow-up. Thus, an ROC as function of time is more appropriate. The time-dependent sensitivity and specificity can be defined into three definitions which are cumulative/dynamic (C/D), incident/dynamic (I/D) and incident/static (I/S). We focus on I/D and I/S definitions in this presentation. Incident sensitivity and dynamic specificity use a pre-defined time point for discriminating between individuals who failed and individuals who remained disease-free while static specificity uses a time interval. Further, I/D definition is used for a single marker while I/S definition is used for longitudinal marker. We review the current estimation methods and compare their behaviour in practice using a real dataset in primary biliary cirrhosis.

**Keywords**: ROC, time-dependent, accuracy, biomarker, event-time, longitudinal data

## P12 Bayesian network approach to assessment of medical technologies

### Melody Ni, Jeremy Huddy, Simone Borsci, George Hanna

#### NIHR-Diagnostic Evidence Cooperative, Imperial College London, London, UK

##### **Correspondence:** Melody Ni (z.ni@imperial.ac.uk)

Over the past few decades, an unprecedented number of medical devices emerged, including point-of-care tests (POCTs). Convenience and accessibility support timely clinical decision-making, promoting better, more efficient care. Despite the potential, however, the use of POCTs is still limited in the UK. Adoptions are crippled by immediate and definitive costs but future and uncertain benefits. Bayesian networks are graphical tools for dealing with uncertainties. Within BNs, events are represented by circles and their relationship by arrows. BNs offer an intuitive way of understanding uncertainties. In this research, we use BNs to map out adoption and evidence generation process involved in assessing medical technologies. We demonstrate how BNs can help decision makers understand the roles and stages of evidence generation, importance of engaging with stakeholders, achieving effective communications and more efficient allocation of resources.

**Fig. 5 (abstract P12).**
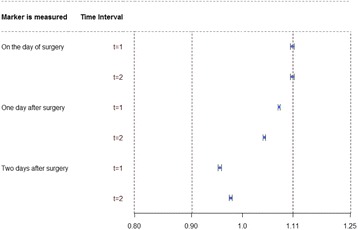
See text for description

## P13 Urinary biomarkers for acute kidney injury: study and validation

### Teresa Pérez^1^, M.Carmen Pardo^2^, Angel Candela-Toha^3^, Alfonso Muriel^4^, Javier Zamora^5,6^

#### ^1^Department of Statistics and OR III, Complutense University of Madrid, Madrid, Spain; ^2^Department of Statistics and OR I, Complutense University of Madrid, Madrid, Spain; ^3^Department of Anesthesia and Reanimation, Ramón y Cajal Hospital (IRYCIS), Madrid, Spain; ^4^Department of Clinical Biostatistics, Ramón y Cajal Hospital (IRYCIS), Madrid, Spain; ^5^Department of Clinical Biostatistics, Ramón y Cajal Hospital (IRYCIS), Madrid, Spain; ^6^Barts and The London School of Medicine and Dentistry, Queen Mary University, London, UK

##### **Correspondence:** Teresa Pérez (teperez@estad.ucm.es)

**Background**: Cardiac surgery-associated acute kidney injury (CSA-AKI) has received increasing attention over the last years, when new biomarkers have been discovered and some of them have been broadly tested. Unfortunately, none has reached the clinical phase and diagnosis and classification are still based on serum creatinine (sCr) and diuresis. We investigate alternative biomarkers to predict CSA-AKI.

Design: It is a retrospective study of consecutive patients undergoing major cardiac surgery using a computerized database with prospectively collected data. The total sample was divided in two halves, the exploratory sample, used to achieve the main objectives and the validation sample, used to validate the results.

**Methods**: The ability of the biomarkers to predict AKI in advance was measured with time-dependent ROC curves. We evaluated if the results in the validation sample were similar to the results in the exploratory one. The simplest and most widely used approach to test equivalence is the two one-sided test (TOST). Equivalence is accepted when the 90% confidence interval (CI) of mean AUC difference between the exploratory sample and the validation sample fall within the acceptance limits (0.8 to 1.25).

**Fig. 6 (abstract P13).**
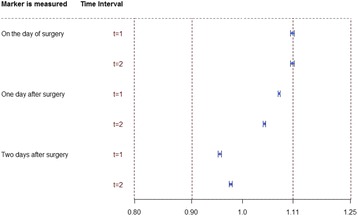
The 90% CI of mean AUC differences between the exploratory sample and the validation sample corresponding to severe AKI. Dotted line are the equivalence margin with Δ = 20% (*wider interval*) and Δ = 10% (*narrower interval*)

**Results**: An AKI event was developed in 610 out of 1980 (30.7%) patients, in the exploratory sample. One of the biomarker studied showed a good performance for AKI prediction, with values for the area under the ROC curve between 0.70 and 0.94. Similar results were obtained in the validation sample where equivalence was accepted in all cases, Fig. 6.

**Interpretation**: An alternative biomarker to sCr has been obtained to predict AKI events after cardiac surgery using time-dependent ROC curves as the statistical tool to measure its performance. The results in the validation sample are similar to the results in the exploratory sample then we can be reassured about its accuracy.

## P14 Key issues in model-based economic evaluations of prostate cancer screening: a systematic review of methods

### Sabina Sanghera^1^, Syed Mohiuddin^1^, Richard Martin^2^, Jenny Donovan^2^, Joanna Coast^1^

#### ^1^Health Economics at Bristol (HEB), School of Social and Community Medicine, University of Bristol, Bristol, UK; ^2^School of Social and Community Medicine, University of Bristol, Bristol, UK

##### **Correspondence:** Sabina Sanghera (sabina.sanghera@bristol.ac.uk)

**Background**: There is an ongoing debate about the harms and benefits of implementing a national screening programme for prostate cancer. The screening test (Prostate Specific Antigen, PSA) has poor sensitivity and specificity, meaning it produces a high proportion of false positive and false negative results. The PSA screen is followed by a biopsy which itself has relatively poor sensitivity. The subsequent treatments for screen and biopsy detected prostate cancer impact significantly on quality of life and resources.

Men may have screen-detected prostate cancer but the disease may never develop to cause symptoms within their lifetime. The potential benefits of screening are for those who have prostate cancer that is destined to progress because the cancer is identified sooner than in clinical practice and is treated earlier, potentially improving life expectancy. Men with a false positive PSA test result are, on the other hand, subjected to unnecessary tests and treatment, whilst the men with a false negative result (and with prostate cancer that is destined to progress) may have necessary treatment delayed.

Since the mid-1990s, there have been many model-based economic evaluations published assessing the cost-effectiveness of screening for prostate cancer, using a variety of modeling methods that in turn may impact the results from the model.

**Objective**: To summarise existing methods used in decision models of prostate cancer screening, and to identify key issues and areas for further methodological development.

**Method**: Systematic review of modelling studies from electronic databases and grey literature that assess PSA screening for prostate cancer. The review follows guidance from the Centre for Reviews and Dissemination and is restricted to evidence from the last 10 years to reflect current practice in screening for prostate cancer and economic evaluation methods. Information is extracted on model type, structure and calibration, how the natural history of disease has been handled, and the extent to which uncertainty in the cost-effectiveness result has been quantified.

**Results**: 12 modelling studies have been identified to date in settings across Europe, USA, and Asia. Analysis suggests that model types range from decision tree to discrete event simulation, different approaches to model calibration are adopted and the conditional dependence between the screen and biopsy is rarely mentioned. The quality of life values used are crucial to the results and are often taken from outdated studies using a variety of elicitation methods. Numerous assumptions are made due to the limited availability of relevant information on disease natural history, and these assumptions do not include clinical input. The methods for assessing overdiagnosis are inconsistent.

**Discussion**: Despite the relatively large number of models, there are still important issues when modelling prostate cancer screening that can impact the results and require refinement.

## P15 A systematic review of the cost-effectiveness of diagnostic biomarkers for metastatic colorectal cancer in the context of targeted therapies

### Mikyung Kelly Seo^1^, John Cairns^1,2^

#### ^1^London School of Hygiene and Tropical Medicine, London, UK; ^2^University of Bergen, Bergen, Norway

##### **Correspondence:** Mikyung Kelly Seo (seokelly@gmail.com)

**Background**: In recent years, advances in targeted therapies for the treatment of mCRC have elevated the expectations of biomarker-guided treatments because a biomarker may improve health outcomes by delivering the right treatment to the right patient while avoiding an unnecessary treatment of patients unresponsive to the therapy. However, the cost-effectiveness (CE) of biomarkers remains unclear. This study aims to review recent literature on the CE of biomarkers together with targeted therapies for mCRC.

**Method**: A literature search was performed using MEDLINE (Ovid) and National Health Service Economic Evaluation Database (NHSEED). Cost-effectiveness as well as cost-utility studies were identified. The study selection was based on the inclusion and exclusion criteria formulated by the framework of PICOS (population, intervention, comparators, outcomes, study types). Publications from 2010 through February 2016 were searched.

**Result**: Five hundrend sixty-six publications were searched in total, and twenty-two papers were included in the review. Of these, twenty studies were model-based analyses including Markov models and area under curve (AUC) models, and two were trial-based. The number of strategies compared ranged from two to seven arms per study. Overall, the CE of targeted therapies with or without biomarkers produced conflicting results. No studies on bevacizumab, aflibercept and regorafenib considered the use of biomarkers. However, two studies reported incremental cost-effectiveness ratios (ICERs) on patients with wild-type Kirsten rat sarcoma (KRAS). As for ramucirumab, no economic evaluations published to date.

Meanwhile, all studies of cetuximab and panitumumab have considered the use of biomarkers. Overall, the CE results varied depending on different combination of strategy arms consisting of: (a) no prior biomarker test performed and all patients treated with intervention, (b) no prior biomarker test performed and all patients treated with best supportive care(BSC)/chemotherapy, and (c) biomarker test performed and treatment guided by the biomarker result (wild-type KRAS patients receive the intervention and mutant KRAS patients receive BSC/chemotherapy). Ten studies analysed cetuximab including seven studies as a monotherapy and six studies as a combination therapy. For cetuximab strategy (a) was never cost-effective compared to strategy (b) and (c). However, when strategy (c) was compared to strategy (b), it revealed conflicting results; two studies indicated that it was cost-effective, and two that it was not cost-effective. Six studies were done on panitumumab and they showed similar patterns of CE results depending on the different combination of strategies.

**Conclusion**: Our review found that the choice of comparators is a key driver in determining the CE of biomarkers in the context of targeted therapies. Strategies that ‘treat all with intervention without KRAS testing’ were not cost-effective compared to ‘treat all with BSC/chemotherapy without testing’ strategies. In contrast, ‘treat WT KRAS only with testing’ is either cost-effective or cost-saving when compared to ‘treat all with intervention without testing’. It was then shown mixed results when ‘treat with testing’ was compared with ‘treat all with BSC/chemotherapy without testing’. Whether or not use of biomarkers to inform the treatment decision is cost-effective is largely driven by the expected impact on health outcomes.

## P16 Review strategies to inform research prioritization of biomarkers: AKI-Diagnostics case study

### Elizabeth Mitchell^1^, Alison Smith^1^, Judy Wright^1^, Peter Hall^3^, Michael Messenger^2^, Nicola Calder^4^, Nyantara Wickramasekera^1^, Karen Vinall-Collier^5^, Andrew Lewington^2^

#### ^1^Leeds Institute of Health Sciences, University of Leeds, Leeds, UK; ^2^NIHR Diagnostic Evidence Co-Operative Leeds , Leeds Institute of Health Sciences, University of Leeds, Leeds, UK; ^3^The Institute of Genetics and Molecular Medicine, Edinburgh Cancer Research Centre, University of Edinburgh, Edinburgh, UK; ^4^Leeds Teaching Hospitals NHS Trust, St James’s University Hospital, Leeds, UK; ^5^School of Dentistry, University of Leeds, Leeds, UK

##### **Correspondence:** Judy Wright (J.M.Wright@leeds.ac.uk)

**Background**: Numerous biomarkers for the early diagnosis and monitoring of Acute Kidney Injury (AKI) have recently been developed. Evidence on their clinical validity and utility is variable, and there is a time-limited opportunity to propose an efficient future research strategy for AKI diagnostics in the UK, to inform optimal test-reimbursement decisions. The ongoing NIHR AKI-Diagnostics study aims to inform future research by conducting a systematic review and early economic evaluation of biomarkers for AKI in the critical care setting.

**Methods**: A two-stage search process was adopted. Stage 1 consisted of a broad scoping search of world literature to identify candidate biomarkers for evaluation. The results were used to produce a ranked shortlist of priority biomarkers according to criteria agreed via expert consensus: volume and currency of evidence, number of samples studied, and biological plausibility. Stage 2 (underway) consists of a systematic review to identify evidence on the analytical and clinical validity, and clinical utility of the priority biomarkers.

**Results**: The scoping search identified 4,804 references. After screening by titles/abstract, 487 potentially relevant papers remained, relating to 152 individual biomarkers. Those already used in standard care (11; including serum creatinine) or with incomplete data related to the dimensions outlined above (19) were excluded. Ten priority biomarkers/tests were shortlisted: BNP, Cystatin C, IL-6, IL-18, KIM-1, L-FABP, NAG, Nephrocheck©, NGAL and TNF-α. The subsequent systematic review has identified 5,071 citations. Data extraction to date has focused on the top 3 tests: Nephrocheck© (which has received significant marketing and is the only FDA licensed test), Cystatin C and NGAL (both with the greatest volume of evidence). Currently, 110 papers have been included: 6 Nephrocheck©, 48 Cystatin C, 56 NGAL.

**Reflections**: Several key issues were encountered in the review. First, in the absence of published guidance, the test shortlisting criteria were developed by expert consultation, and may not capture promising in-development tests due to the pragmatic focus on objective criteria (e.g. volume of evidence). Second, the volume of evidence was substantially greater than originally indicated by pre-study scoping searches, largely due to the decision to broaden the final scope to include tests developed outside the critical care setting. Together with the number of candidate tests identified (including multiple tests used in conjunction) and the complexity of data extraction involved, this resulted in extended study timelines. Finally, poor reporting, especially of analytical factors, makes comprehensive synthesis of test analytical and clinical validity difficult.

**Conclusion**: As the number of biomarkers entering the healthcare market continues to rapidly expand, the role of reviews to inform future research priorities is becoming increasingly important. The two-stage search process outlined here represents a novel approach in this area; however, it is clear that further work is required to establish efficient and optimal search strategies and shortlisting criteria for such reviews.

## P18 How treatment use is addressed in prognostic research: a systematic review

### Romin Pajouheshnia^1^, Johanna Damen^1,2^, Rolf Groenwold^1^, Karel Moons^1^, Linda Peelen^1^

#### ^1^Julius Center for Health Sciences and Primary Care, University Medical Center Utrecht, Utrecht, The Netherlands; ^2^Cochrane Netherlands, University Medical Center Utrecht, Utrecht, The Netherlands

##### **Correspondence:** Romin Pajouheshnia (R.Pajouheshnia@umcutrecht.nl)

**Background** Prognostic models are often designed to estimate an individual’s future risk of disease given that they are not receiving a certain treatment and will remain untreated. In practice, individuals enrolled in studies that aim to develop or validate such models may receive treatment to prevent the outcome of interest during the study. This can lead to an underestimation of the true untreated risk in those who were treated, which may impact upon the accuracy or validity of newly derived models, or may bias the findings of a validation study. It is not yet clear how and to what extent treatment use is being addressed in prognostic modelling studies.

**Objectives** To provide insight into the degree to which relevant treatment information is reported and handled in the derivation and validation of prognostic models, and what impact this may have, using the field of cardiovascular risk prediction as an example.

**Methods** For the current study, we made use of a previously conducted systematic review (search: June 2013) to identify articles that reported prognostic models in the field of cardiovascular preventative medicine, in a general population setting. Data were collected on the reporting of treatments (blood pressure, lipid and other medications, surgical procedures and lifestyle modifications), including the frequency and timing of treatment use, how treatments were handled in the analysis, and any discussion regarding the implications of treatment use.

**Results** The search strategy yielded 9965 unique titles, of which 302 articles were included for the current analysis.

Of these articles, 91 (30%) did not mention treatments with respect to the characteristics of study participants, prediction modelling, or their relevance to the findings of the study. 146 articles (48%) reported specific information about treatment use at study entry; 78 articles (26%) provided information about more than one treatment. Information about changes in medication use during follow-up was rare (1%). Treatment effects were accounted for in 79 articles (26%) by including only individuals without a certain treatment in the analysis. Of all the articles that developed a model, 80 included treatment use at baseline as a predictor; changes in treatment during follow-up were not modelled. Possible implications of treatment use with respect to model performance or applicability were discussed in only 24 articles (8%).

**Conclusions** This review finds that treatment use has largely not been addressed in cardiovascular prognostic modelling studies. The absence of treatment information in reporting may lead to difficulties when validating or implementing a prognostic model, and may lead to uncertainty over whether a model will provide correct risk estimates when used in practice. Greater consideration is needed when collecting, reporting and handling treatment information.

## P19 A framework for the quality assessment of measurement proceedures using in vitro diagnostic medical devices (IVDs)

### Michael Messenger^1^, David Cairns^2^, Alison Smith^3^, Michelle Hutchinson^4^, Judy Wright^3^, Peter Hall^5^, Nicola Calder^1^, Cathie Sturgeon^6^, Liz Mitchel^3^, Rebecca Kift^7^

#### ^1^NIHR Diagnostic Evidence Co-Operative Leeds, Leeds Teaching Hospitals NHS Trust, St James’s University Hospital, Leeds, UK; ^2^Leeds Institute of Clinical Trials Research, University of Leeds, Leeds, UK; ^3^Leeds Institute of Health Sciences, University of Leeds, Leeds, UK; ^4^Leeds Institute of Cancer and Pathology, University of Leeds, Leeds, UK; ^5^The Institute of Genetics and Molecular Medicine, Edinburgh Cancer Research Centre, University of Edinburgh, Edinburgh, UK; ^6^UK NEQAS [Edinburgh]. Department of Laboratory Medicine, Royal Infirmary, Edinburgh, UK; ^7^Department of Blood Sciences, Old Medical School, Leeds General Infirmary, Great George Street, Leeds, UK

##### **Correspondence:** David Cairns (d.a.cairns@leeds.ac.uk)

n Vitro Diagnostic (IVD) medical devices form the basis of ~70% of clinical decision making in the NHS. The accuracy and associated uncertainty surrounding diagnostic testing consequently has a major impact on the overall quality of clinical decisions and subsequent clinical and cost effectiveness. Numerous pre-analytical, analytical and biological factors can contribute to the measurement uncertainty in diagnostic testing proceedures. These uncertainties accumulate through the measurement system and may introduce bias into clinical trials; contribute towards heterogeneity between biomarker research studies; and limit the applicability of research findings to clinical practice. Whilst prior reports have highlighted the scale and impact of these issues and reporting guidelines have been published (e.g. BRISQ, PROBE-ME), we are not aware of any methods in use for evaluating the quality and appropriateness of measurement procedures within systematic reviews of IVDs. We suggest that this is limiting the ability of systematic reviewers and health technology assessors to fully evaluate risk and model uncertainty within assessments. This has been highlighted in several recent NICE diagnostic assessment programme reports.

**Fig. 7 (abstract P19).**
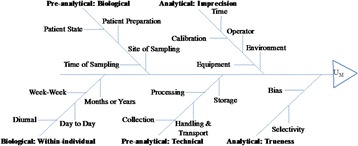
Feather diagram depicting factors that may contribute to measurement uncertainty (UM)

As part of an NIHR funded “Multi-Centre Programme into the Evaluation of Biomarkers Suitable for Use in Patients with Kidney and Liver Diseases” and an NIHR funded health technology assessment “AKI-Diagnostics” key parameters for consideration by systematic reviewers were identified (Fig. 7) and an initial framework for assessing the quality of measurement procedures developed. Pilot evidence from early testing of this template will be presented and has proved useful in highlighting inadequacies in the reporting and conduct of measurement procedures that may introduce bias, irreproducibility or inapplicability. However, further work is required to refine the parameters and signaling questions for inclusion within the framework, develop guidance for users and validate its utility more widely.

## P20 Earlier detection of acute rejection in kidney transplantation using a biomarker-based signature derived from longitudinal gene-expression data

### Sofia Christakoudi^1,7^, Manohursingh Rungall^1,2,3^, Paula Mobillo^1,2^, Rosa Montero^1,3^, Tjir-Li Tsui^2^, Sui Phin Kon^4^, Beatriz Tucker^4^, Steven Sacks^1^, Chris Farmer^5^, Terry Strom^5^, Paramit Chowdhury^6^, Irene Rebollo-Mesa^1,7^, Maria Hernandez-Fuentes^1,2^

#### ^1^MRC Centre for Transplantation, DTIMB, King’s College London, London, UK; ^2^NIHR Comprehensive Biomedical Research Centre at Guy’s and St Thomas’ NHS Foundation Trust in partnership with King’s College London and King’s College Hospital, London, UK; ^3^Guy’s and St Thomas’ NHS Foundation Trust, London, UK; ^4^King’s College Hospital NHS Foundation Trust, London, UK; ^5^East Kent Hospitals University NHS Foundation Trust, Canterbury, UK; ^6^Department of Medicine, Transplant Institute, Beth Israel Deaconess Medical Center, Harvard Medical School, Boston, MA, USA; ^7^Department of Biostatistics, Institute of Psychiatry, Psychology and Neuroscience, King’s College London, London, UK

##### **Correspondence:** Sofia Christakoudi (sofia.christakoudi@kcl.ac.uk)

**Background**: Biopsy is a golden standard for diagnosis of acute rejection in kidney transplantation, but is associated with risks and is conducted when graft dysfunction is manifest and graft damage has occurred. Therefore, there is a need of a non-invasive method for early identification of rejection prior to graft damage.

**Samples**: Five hundrend seventy-three whole-blood samples were collected at intervals post transplantation from 69 kidney transplant recipients (KTR), part of the cohort KALIBRE study (Kidney Allograft Immune Biomarkers of Rejection Episodes). 23 KTRs had experienced biopsy-proven acute cellular rejection at a median time post transplantation 110 days (min-max 6–364 days) and 46 KTRs had stable kidney function with serum creatinine within 15% from a baseline and no clinical signs of graft failure for the first post-transplant year.

**Methods**: RT-qPCR expression was examined for 22 literature-based genes, including the Allomap signature of heart transplant acute rejection (1), as well as Granzyme B, Perforin, Fas-Ligand, FoxP3, IP-10, IL-15, TGFβ, CXCR3, NGAL, INFγ and RORc. Time-adjusted residuals were generated from gene expression data using linear mixed-effects models fitted to samples from stable patients, accounting for changes unrelated to rejection. A gene signature was selected based on penalised Cox regression using as time-independent covariates the time-adjusted residuals for a sample prior to and nearest to rejection for rejectors or the mean of all samples for each stable patient. Longitudinal data were used as a test set.

**Fig. 8 (abstract P20).**
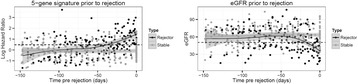
See text for description

**Results**: For the timepoint close to rejection: eGFR alone showed AUC 0.90 (95% confidence interval 0.83–0.97); the Allomap gene set showed AUC 0.76 (0.62–0.89); the full model with 22 genes showed AUC 0.87 (0.78–0.96); a newly-selected 5-gene signature, containing two Allomap genes, showed comparable performance to the 22-gene set and eGFR: AUC 0.87 (0.77–0.96). A cut-off of 0.48 for the log hazard ratio (logHR) relative to an average patient would provide sensitivity 0.87, specificity 0.80, PPV 0.69 and NPV 0.93, similar to eGFR with cut-off 50.4 ml/min/1.73 m2 (sensitivity 0.83, specificity 0.78, PPV 0.66, NPV 0.90). However, taking into account the longitudinal follow-up of logHR predictions from a Cox model with time-independent co-variates for the 5-gene signature, rejections could be indicated approximately two weeks earlier compared to eGFR (see Fig. 8).

**Conclusion**: Molecular markers of rejection in blood emerge well ahead of the time of clinically presented acute rejection. Monitoring of gene expression signatures for early detection of acute rejection is promising.

**Reference**

(1) Deng MC et al. Am J Transplant 2006; 6(1):150–60

## P21 Performance of the Framingham risk models and Pooled Cohort Equations for the prediction of cardiovascular disease in the general population: a meta-analysis

### Johanna A. A. G. Damen^1^, Thomas P. A. Debray^1^, Pauline Heus^2^, Lotty Hooft^2^, Karel G. M. Moons^3^, Romin Pajouheshnia^3^, Johannes B. Reitsma^1^, Rob J. P. M. Scholten^2^

#### ^1^Julius Center for Health Sciences and Primary Care, and Cochrane Netherlands, University Medical Center Utrecht, Utrecht, The Netherlands; ^2^Cochrane Netherlands, Julius Center for Health Sciences and Primary Care, University Medical Center Utrecht, Utrecht, The Netherlands; ^3^Julius Center for Health Sciences and Primary Care, University Medical Center Utrecht, 3584 CG Utrecht, Netherlands

##### **Correspondence:** Johanna A. A. G. Damen (j.a.a.damen@umcutrecht.nl)

**Background**: The implementation of the Framingham risk models and Pooled Cohort Equations (PCE) is currently recommended in the United States for predicting ten-year risk of developing cardiovascular disease (CVD) in individuals. Over the past few years, these prediction models have been extensively validated in other individuals, settings and countries.

**Objectives**: To systematically review and summarize the discrimination and calibration of three CVD prediction models, and to determine heterogeneity in this performance across subpopulations or geographical regions.

**Methods**: In December 2015, Medline and Embase were searched for studies investigating the external validation of three CVD risk equations (Framingham Wilson 1998, Framingham ATP III 2002 guideline and PCE 2013). This search was combined with a search in Web of Science and Scopus for citations of these three articles. Studies published before June 2013 were identified from a previous review in which we mapped all CVD risk prediction models until that date. Studies were eligible for inclusion if they externally validated the original prediction model without updating, in a general population setting. Critical appraisal was performed based on the CHARMS checklist. Data were extracted on participant selection, case-mix, essential study design characteristics, and model performance (quantified by the c-statistic and observed/expected ratio). Performance estimates were summarized using random effects meta-analysis models that accounted for differences in case-mix to explore sources of heterogeneity.

**Results**: The search identified 10,687 references, of which 1,501 were screened in full text and 45 met our eligibility criteria. These articles described the external validation of Framingham Wilson (25 articles), Framingham ATP III (15 articles) or the PCE (10 articles). Our meta-analytical results will be presented during the MEMTAB symposium as we are currently meta-analyzing the results. We will present the overall range of performance of the three risk equations and attempt to compare these to each other. Furthermore, we will present the range of performance for case-mix differences such as age, comorbidities and treatment.

**Conclusion**: The results of this study can help in identifying which of these three CVD models can reliably be used, whether there is heterogeneity in their performance and whether there are subpopulations for which further research is necessary to improve CVD risk prediction.

## P22 Systematic review of prediction models for cardiovascular disease risk in the general population: stop developing, start validating

### Johanna A. A. G. Damen^1^, Lotty Hooft^2^, Ewoud Schuit^1^, Thomas P. A. Debray^1^, Gary S. Collins^3^, Ioanna Tzoulaki^4^, Camille M. Lassale^4^, George C. M. Siontis^5^, Virginia Chiocchia^3^, Corran Roberts^3^, Michael Maia Schlüssel^3^, Stephen Gerry^3^, James A. Black^6^, Pauline Heus^2^, Yvonne T. van der Schouw^7^, Linda M. Peelen^7^, Karel G. M. Moons^7^

#### ^1^Julius Center for Health Sciences and Primary Care, and Cochrane Netherlands, University Medical Center Utrecht, Utrecht, The Netherlands; ^2^Cochrane Netherlands, Julius Center for Health Sciences and Primary Care, University Medical Center Utrecht, Utrecht, The Netherlands; ^3^Centre for Statistics in Medicine, Nuffield Department of Orthopaedics, Rheumatology and Musculoskeletal Sciences, University of Oxford, Oxford, UK; ^4^Department of Epidemiology and Biostatistics, School of Public Health, Imperial College London, London, UK; ^5^Department of Cardiology, Bern University Hospital, 3010 Bern, Switzerland; ^6^MRC Epidemiology Unit, University of Cambridge School of Clinical Medicine, Cambridge, UK; ^7^Julius Center for Health Sciences and Primary Care, University Medical Center Utrecht, Utrecht, Netherlands

##### **Correspondence:** Johanna A. A. G. Damen (j.a.a.damen@umcutrecht.nl)

**Background**: Cardiovascular disease (CVD) is a leading cause of morbidity and the leading cause of mortality worldwide. Many prediction models have been developed to assess individual CVD risk to allow targeting of preventive treatment.

**Objectives**: To provide an overview of all prognostic models that predict future risk of CVD in the general population, and to describe their reporting on predicted outcomes, study populations, predictors, and methods.

**Methods**: In June 2013 a systematic search was performed in Medline and Embase to identify studies that described the development or external validation of a model predicting CVD in the general population.

**Results**: Nine thousand nine hundred sixty-five references were identified, of which 1388 were screened in full text. 212 articles were included in the review, describing the development of 363 prediction models and 473 external validations. Most models were developed in Europe (n = 167, 46%), predicted risk of coronary heart disease (n = 118, 33%), over a 10-year period (n = 209, 58%). The most common predictors were smoking (n = 325, 90%) and age (n = 321, 88%), and the majority of models was sex-specific (n = 250, 69%). Substantial heterogeneity in predictor and outcome definitions was observed between models and important clinical and methodological information was often missing. For 49 models (13%) the prediction time horizon was not specified and for 92 (25%) crucial information was missing to actually use the model for individual risk prediction. Only 132 developed models (36%) were externally validated and only 70 (19%) by independent investigators. Model performance was very heterogeneous and measures such as discrimination and calibration were reported for 65% and 58% of the external validations respectively.

**Conclusion**: There is an excess of models predicting incident CVD in the general population. The usefulness of the majority of the models remains unclear due to methodological shortcomings, incomplete presentation, and lack of external validation and model impact studies. Rather than developing yet another similar CVD risk prediction model, future research should focus on externally validating and head-to-head comparisons of the promising existing CVD risk models, on tailoring these models to local settings or even combining them, and investigating whether they can be extended by addition of new predictors.

## P23 Design characteristics of external validation studies influencing the performance of risk prediction models

### Johanna A. A. G. Damen^1^, Thomas P. A. Debray^1^, Pauline Heus^2^, Lotty Hooft^2^, Karel G. M. Moons^3^, Romin Pajouheshnia^3^, Johannes B. Reitsma^1^, Rob J. P. M. Scholten^2^

#### ^1^Julius Center for Health Sciences and Primary Care, and Cochrane Netherlands, University Medical Center Utrecht, Utrecht, The Netherlands; ^2^Cochrane Netherlands, Julius Center for Health Sciences and Primary Care, University Medical Center Utrecht, Utrecht, The Netherlands; ^3^Julius Center for Health Sciences and Primary Care, University Medical Center Utrecht, 3584 CG Utrecht, Netherlands

##### **Correspondence:** Johanna A. A. G. Damen (j.a.a.damen@umcutrecht.nl)

**Background**: Meta-epidemiological studies have shown that study results are directly influenced by study design characteristics. The results of a randomized trial may for example be biased by inadequate allocation concealment, lack of blinding of outcome assessments, exclusion of participants (e.g. due to selective loss to follow-up) and reporting of intermediate outcomes. The diagnostic accuracy of tests may be overestimated in case-control studies, and the choice of reference standards can lead to biased study results. Meta-epidemiological studies assessing the influence of design features on the results of prognostic research are yet missing.

**Objectives**: To determine which design characteristics of a study influence the performance of a prognostic model upon external validation, taking the validations of three established risk prediction models for cardiovascular disease (CVD) as an example.

**Methods**: In December 2015, Medline and Embase were searched for articles investigating the external validation of three CVD risk equations (Framingham Wilson 1998, Framingham ATP III 2002 guideline and Pooled Cohort Equations (PCE) 2013). This search was combined with a search in Web of Science and Scopus for citations of these three articles. Studies published before June 2013 were identified from a previous review in which we mapped all CVD risk prediction models until that date. Studies were eligible for inclusion if they externally validated the original prediction model without updating, in a general population setting. Data were extracted on essential study design characteristics. By conducting a random effects meta-regression of model performance statistics (c-statistic and observed/expected ratio), we will determine which study characteristics influence model performance statistics.

**Results**: The search identified 10,687 references, of which 1,501 were screened in full text and 45 met our eligibility criteria. These articles described the external validation of Framingham Wilson (25 articles), Framingham ATP III (15 articles) and the PCE (10 articles). Our meta-analytical results will be presented during the MEMTAB symposium as we are currently meta-analyzing the results. We will present the range of performance for the three prediction models for different design characteristics, including study design (e.g. cohort, case control), median follow-up time, total sample size, assessment of predictors and outcomes, and handling of missing data.

**Conclusion**: This study will identify design characteristics influencing the performance of CVD risk prediction models in external validation studies. This information will help when interpreting the potential impact of validation studies with certain design flaws, and thereby facilitate risk of bias assessment in systematic reviews of prognostic studies.

## P24 Clinical prediction models: a critical review of online risk calculators

### Jie Ma, Doug Altman, Gary Collins

#### Centre for Statistics in Medicine, Nuffield Department of Orthopaedics, Rheumatology and Musculoskeletal Sciences, University of Oxford, Oxford, UK

##### **Correspondence:** Jie Ma (jie.ma@ndorms.ox.ac.uk)

**Background**: Clinical prediction models are increasingly made available on the Internet or as applications for smart phones. These models are available for use to both clinicians and the general public. However, the evidence of whether the model should be used is often unclear.

**Aim**: The aim of this study was to review the availability of clinical prediction models as calculators on the Internet and identify the evidence base on the performance of the model. We also provide some guidance on principles to follow when preparing a prediction model calculator to be made available on the Internet.

**Methods**: In March 2015, the Google search engine was used with a combination of fifteen search terms that described the concepts of prediction and calculator. For each search term, the first 50 hits (total 750 websites) were recorded. Websites that presented an online clinical prediction model that required manual entry of patient level information that produced a probability or risk of having an undiagnosed condition (diagnostic) or a probability or risk of developing a health condition in the future (prognostic), were eligible. Information such as the background of the model (e.g. country), intended patient population, intended user of the model (clinician or patient), information on how the prediction models were developed and any details about their validation model, and finally information on how the web-calculators are presented (graphical, text, lay terminology) and to be used were extracted using a pilot-tested extraction form.

**Results**: A total of 116 models were included; only less than half of the websites cited references to the articles describing the development of model and only 8 websites cited references for the validation of the model on the website. Most of the prediction models are poorly documented on the Internet, with little information to help users actually use them. In many instances, it was unclear on who the prediction calculator was intended for (with only 44 mentioning the target group), less than 20% of websites provided help to use the model (including frequently asked questions). Only 25 models reported the description for each risk factors, nearly half of the models (n = 56) presented no information or checks on the ranges of any continuous factors. Furthermore, many calculators (n = 33) did not display warning messages when information is entered incorrectly.

**Conclusion**: Prediction models are widely available on the Internet to support decision-making for clinicians and general public, yet the information presented alongside the models is inadequate, should be used with care.

## P25 Does CRP have value as a marker of infection or inflammation in the elderly? An investigation using samples taken in primary care

### Graeme Spence^1^, David McCartney^1^, Ann van den Bruel^1^, Daniel Lasserson^2^, Gail Hayward^1^

#### ^1^Nuffield Department of Primary Care Health Sciences, University of Oxford, Oxford, UK

^2^Nuffield Department of Medicine, University of Oxford, Oxford, UK

##### **Correspondence:** Graeme Spence (graeme.spence@phc.ox.ac.uk)

**Background**: Diagnosis of infection in the elderly primary care population is a clinical challenge, and point of care CRP testing could offer diagnostic benefit. However, GPs have expressed doubts over the value of CRP in the context of an aging immune system. The use of C-reactive protein (CRP) as a reliable marker of severe infection in the elderly undifferentiated primary care population has not been sufficiently validated, and the relationship between elevated CRP and neutrophils, another blood marker of infection, has not been well characterised.

**Objectives**: To examine 1) the proportion of the elderly primary care population on whom CRP is measured currently; 2) the distributions of CRP and neutrophils in blood tests taken in primary care settings; 3) whether correlation and categorization agreement exist between these tests and whether this varies with increasing age; 4) whether a change from baseline CRP is demonstrated in the elderly by examining how CRP varies over short time periods in the same patient.

**Data**: Routinely collected, anonymised and de-identified data was obtained from the main laboratory for Oxford University Hospital NHS Trust. A database was created of all adults who had a blood count taken in primary care within Oxfordshire CCG during 2012 and 2013 (161,225 patients). The database was then populated with the available blood test results for those individuals during that period (478,697 tests).

**Methods**: Individuals with greater than 12 blood tests in the database were excluded to remove those patients undergoing frequent monitoring for chronic disease. Analysis was undertaken on the remaining database (416,989 tests from 158,097 patients). The results for CRP and neutrophils were categorised as abnormal according to laboratory reference ranges (CRP: >5 mg/L, neutrophils >7 × 109/L).

**Results**: Overall, 29% of the target population (registered patients in Oxfordshire) appear in the database, although this varies with age (54% of 85-89 year olds, compared with 25% of under 65 s). 34% of CRP tests were classed as elevated, compared with 10% of neutrophil tests. The proportions of elevated tests increased with age over 65 years old, for example from 37% of CRP tests elevated in 65-69 year olds to 61% in 95-99 year olds. The majority of tests with CRP <50 mg/L displayed neutrophil values <7 × 109/L. For CRP >50 mg/L, approximately half of the corresponding neutrophil tests were not elevated. Little variation in correlation or agreement was observed with age. In patients with a sequence of CRP tests each within 7 days of the previous test, the CRP values were significantly lower on the second test compared to the first (p <0.001), for both under and over 65 year olds.

**Conclusions**: In a large dataset of blood tests taken in primary care, elevated CRP was evident even at the extremes of age, and varied over short time periods. This suggests that it may have potential as a diagnostic marker of acute infection or inflammation even at the extremes of age. Poor correlation between elevated CRP and neutrophils was evident across all age ranges and suggests that CRP can be elevated in the absence of a neutrophilia. A high proportion of the Oxfordshire elderly population had a CRP blood test performed in the two year period of our dataset, suggesting that clinicians do attach importance to this marker. Further work is required to evaluate these findings in a clinical cohort.

## P27 Benefit based strategies and value based strategies should be distinguished when deciding to bring a diagnostic test into use

### Werner Vach (wv@imbi.uni-freiburg.de)

#### Institute for Medical Biometry and Statistics, Medical Faculty and Medical Center, University of Freiburg, Freiburg im Breisgau, Germany

**Background**: In developing new diagnostic tests and assessing their benefit for patients, enrichment designs are one popular design. In enrichment designs, only the test positive patients are randomized to the two treatments of interest, typically the standard treatment currently given to all patients and a new treatment expected to improve patient outcomes in test positive patients. Consequently, we can only conclude that the new treatment is beneficial for test positive patients. It may happen that other studies randomizing (also) test negative patients demonstrate (later) that also the test negative patients benefit from the new treatment. Then there is actually no need for the test: We can improve patient outcomes just by giving the new treatment to all patients. Then we can say that the test has no value. Regulatory and HTA agencies have commented in different manners on the question to which degree results from enrichment studies can be used to justify to bring a test into use.

**Objective**: The different attitudes can be seen as decisions between a value based and a benefit based strategy. In a value based strategy, a test is introduced when the value is proven, and in a benefit based strategy, a test is introduced when the benefit is proven, but later the decision may be changed and the new therapy may be introduced to all patients. We investigate the potential conclusions form enrichment designs and interactions designs about value or benefit. We further investigate the main determinants for the superiority of one strategy over the other by considering 6 different consequences for a single test.

**Results**: Enrichment designs can inform the benefit based strategy and interaction designs or separate studies in test positive and test negative patients can inform the value based strategy. We have to expect that interaction studies are underpowered to come to a definite conclusion about the value of a test. Advantages and disadvantages from the strategies are mainly determined by the influence of the strategies on the timing of performing corresponding studies: The benefit based strategy allows test positive patients to benefit earlier from the new therapy, but the strategy may delay the conduct of interaction studies and hence delay the detection of tests with no value, but with a benefit from the new therapy for all patients.

**Conclusions**: Benefit based strategies are preferable if the risk of off label use and delayed decisions on the value of a test can be limited. If this cannot be achieved by administrative means like conditional approval, the superiority of the two approaches depends on how often the value based strategy would never allow a test of value to come into use and on how often the benefit based strategy may prevent to detect the no-value status of a test.

## P29 Challenges of combining prognostic and diagnostic studies in a systematic review: predictive value of interim FDG-PET/CT in diffuse large B-cell lymphoma

### Antoinette de Jong^1^, Coreline Burggraaff^2^, Otto Hoekstra^1^, Josée Zijlstra^2^, Henrica de Vet^3^

#### ^1^Department of Radiology and Nuclear Medicine, University Medical Center, Utrecht, the Netherlands; ^2^Department of Hematology, VU University Medical Center, Amsterdam, the Netherlands; ^3^Department of epidemiology and biostatistics, VU University Medical Center, Amsterdam, the Netherlands

##### **Correspondence:** Coreline Burggraaff (C.Burggraaff@vumc.nl)

**Objectives**: In this systematic review we assessed the predictive value of interim 18F-fluoro-2-deoxy-D-glucose positron emission computed tomography (FDG-PET/CT) on progression free survival (PFS), event free survival (EFS), and overall survival (OS) in patients with diffuse large B-cell lymphoma (DLBCL) treated with first-line chemotherapy regimens. Studies were designed as prognostic/predictive studies and/or as diagnostic studies. We present the challenge in searching, scoring and outcome presentation of this mix of studies and analyses.

**Methods**

*Search and inclusion criteria*

We performed a search in three databases (Medline, Embase, Cochrane) with languages restricted to English, French, German or Dutch. Search terms consisted of various descriptions of FDG-PET/CT and non-Hodgkin lymphoma. We included full-text publications of original prospective and retrospective studies in which adult DLBCL patients (>10) with first-line treatment for DLBCL received an interim FDG-PET/CT between the first and fifth cycle of chemotherapy. Treatment regimens were not changed based on the outcome of the interim FDG-PET/CT. Data on PFS, EFS, OS and/or diagnostic value of interim FDG-PET/CT were required, with a median follow-up period of at least 24 months.

*Prognostic and diagnostic parameters for several types of outcome measures*

Prognostic studies present Kaplan Meier data and/or hazard ratio’s with PFS, EFS and/or OS as clinical outcome. Diagnostic studies present parameters such as sensitivity, specificity, negative predictive value (NPV) and/or positive predictive value (PPV) using PFS, EFS, OS or the end-of-treatment FDG-PET/CT as reference standard. Primary endpoint is PFS, as this outcome is of greatest clinical interest when performing an interim FDG-PET/CT. OS and EFS are considered secondary outcomes.

QUADAS-2, PROBAST and the CHARMS checklist were used to assess the methodological quality and all prognostic and diagnostic data.

**Results**: We found 47 eligible studies by screening inclusion criteria, title and abstract. Of this selection, 22 studies presented both prognostic and diagnostic results. Twenty-two studies reported only prognostic results, and three studies reported only diagnostic results.

**Discussion**: Focus of the presentation will be on the integration of the prognostic and diagnostic results.

## P30 An overview of systematic reviews summarising the accuracy of brief cognitive assessments for identifying dementia in primary care

### Harriet Hunt, Chris Hyde

#### Exeter Test Group, University of Exeter Medical School, Exeter, UK

##### **Correspondence:** Harriet Hunt (h.a.hunt@exeter.ac.uk)

**Background**: In the UK, there is a lack of agreement between leading organizations on which tests should be used for dementia identification in primary care. The accuracy of many of the commonly-used brief cognitive assessments for dementia is imperfect, and guidelines for diagnosis lack consistent direction for health care professionals, policy makers and the public.

**Objectives**: To conduct an overview of existing systematic reviews summarizing the accuracy of brief cognitive assessments for identifying dementia, particularly for use in primary care.

**Methods**: We searched EMBASE, MEDLINE, PsychInfo and the Cochrane Database of Systematic Reviews from inception to 2015. We assessed the quality of included reviews using the Assessment of Multiple Systematic Reviews (AMSTAR) measurement tool and the risk of bias in systematic reviews tool ROBIS. Results were presented narratively with detailed tables summarizing key data.

**Main results**: We identified 13 reviews which included a number of different brief cognitive assessments for identifying dementia in primary care at a range of different thresholds. Included reviews assessed the diagnostic accuracy of 56 different assessments.

Based on diagnostic test accuracy findings, we summarize the existing systematic review evidence, comment on the quality of evidence and make recommendations for research and clinical practice.

**Authors’ conclusions**: This overview has shown that the breadth of diagnostic test accuracy evidence is mixed, and there is not one brief cognitive assessment that clearly emerges as superior to others in terms of test accuracy.

A number of methodological challenges present themselves within this overview. The value of conducting an overview review of diagnostic test accuracy is still debated, and we discuss both our initial aims and how these match against our overview findings. We encountered a number of issues of quality and consistency across the evidence base, and within this presentation we will consider applicability of the evidence and how generalizable this may be to the primary care population of interest.

Finally, we will discuss the different purposes of an overview of systematic reviews of diagnostic test accuracy, and reflect on what other research methods may be helpful to address these objectives.

## P32 The role of care pathway analysis in evaluating clinical tests

### Sara Graziadio^1^, Joy Allen^2^, Louise Johnston^2^, Rachel O’Leary^1^, Michael Power^1^

#### ^1^NIHR Diagnostic Evidence Co-operative Newcastle, Newcastle upon Tyne Hospitals NHS Foundation Trust, Newcastle upon Tyne, UK; ^2^NIHR Diagnostic Evidence Co-operative Newcastle, Newcastle University, Newcastle upon Tyne, UK

##### **Correspondence:** Sara Graziadio (Sara.Graziadio@ncl.ac.uk)

**Background:** The term “care pathway” refers to the journey a patient takes during an episode of healthcare.

Care pathway analysis is the comparison between the current pathway and the proposed pathway if the new test is introduced. It can involve extensive discussions with clinicians, laboratory managers, budget holders, patients and all the relevant stakeholders for that care pathway. Care pathway analyses are often visual representations or computer models.

**The role of care pathway analysis in evaluating clinical tests:** Care pathways are used to map the key management decision points and processes in a clinical scenario. This is used in the evaluation of clinical tests to understand the place(s) and purpose(s) of a diagnostic test, and therefore how it could be an improvement over current practice. The potential value of the test (i.e. potential benefits to patients, the NHS, and budget holders) is easier to identify through care pathway analysis. Additionally, potential barriers to adoption can become evident during discussion with stakeholders, and care pathway analysis can support the design of studies to generate the evidence required to transform the value propositions of the test, from potential to actual.

Typically, care pathways in a specific disease area vary across different healthcare providers and different clinicians. Evaluation of this variation can be a valuable tool to guide a company’s business plan and marketing strategy.

**Workflows and information flows:** Other important pathways to take into consideration when evaluating a new test are the workflows, and information flows. These are taken into account using human factors studies, and service evaluation, for example, to identify who makes the decision to test, who carries out the test, how the results are communicated to the patient, the relevant clinical stakeholders, and the patient’s medical records, and finally, how the result is acted upon.

**Mapping a care pathway:** It is advisable to start mapping the care pathway, the workflows and the information flows, as early as possible in the journey from invention to adoption of a new test. Engaging with, and discussing the care pathway with, the correct stakeholders helps to identify strengths and weaknesses of the new test; clarifying its role and place within the current healthcare system.

## P33 Putting a framework for the IVD evidence development pathway into practice

### Joy Allen^1^, Sara Graziadio^2^, Louise Johnson^1^, Rachel O’Leary^2^, Michael Power^2^, Ray Waters^1^, John Simpson^1^

#### ^1^NIHR Diagnostic Evidence Co-operative Newcastle, Newcastle University, Newcastle upon Tyne, UK; ^2^NIHR Diagnostic Evidence Co-operative Newcastle, Newcastle upon Tyne Hospitals NHS Foundation Trust, Newcastle upon Tyne, UK

##### **Correspondence:** Joy Allen (Joy.Allen@ncl.ac.uk)

**Background**. The NIHR Diagnostic Evidence Co-operative (DEC) Newcastle works closely with industry and academia to help them generate high quality, robust scientific evidence on diagnostic accuracy, clinical utility and cost-effectiveness of IVDs they would like be adopted by the NHS.

**Problem**. There is a lack of clarity among IVD developers (commercial and academic) of the evidence required for adoption into the NHS.

**Fig. 9 (abstract P33).**
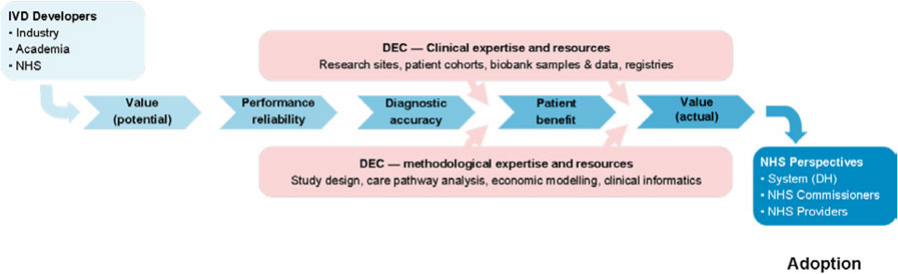
See text for description

**Innovation**. To help IVD developers identify the key value propositions that will determine adoption of their product, the DEC has developed a framework for describing an IVD evidence development pathway (see Fig. 9 below).

Process modelling of care pathways, information flows, and work flows can be used at an early stage of product development to describe the product’s key value propositions, and these define the research strategy needed to provide the supporting evidence.

**Experience with putting the framework into practice**. We will present case studies where the framework for articulating the evidence development pathway has helped industry draft evidence development strategy, which is sequenced and stage-gated for cost and capital efficiency.

We have developed guidelines for IVD developers on the practical application of the evidence development framework, and report on how it may be used to help IVD developers to understand where their product sits in the landscape of evidence generation and their next steps in demonstrating patient benefit, value for money and affordability.

## P34 Generating evidence for evaluations of in vitro diagnostic devices: experiences from the National Institute for Health Research (NIHR) Diagnostic Evidence Co-operative (DEC) Newcastle

### Louise Johnston^1^, Joy Allen^1^, Sara Graziadio^2^, Rachel O’Leary^2^, Ray Waters^1^, Michael Power^2^

#### ^1^NIHR Diagnostic Evidence Co-operative Newcastle, Newcastle University, Newcastle upon Tyne, UK; ^2^NIHR Diagnostic Evidence Co-operative Newcastle, Newcastle upon Tyne Hospitals NHS Foundation Trust, Newcastle upon Tyne, UK

##### **Correspondence:** Louise Johnston (Louise.Johnston@ncl.ac.uk)

The National Institute for Health Research (NIHR) Diagnostic Evidence Co-operative (DEC) Newcastle was established in 2013 with the remit of helping to generate evidence on the clinical utility, cost-effectiveness, and care pathway benefits of in vitro diagnostic devices (IVDs). The NIHR DEC Newcastle works collaboratively with experts from across the National Health Service NHS, academia, and industry, and has very strong links with patient research support groups in the North East of England. Our management group includes representation from the National Institute for Health and Care Excellence (NICE) to help ensure that evidence generated is relevant to the NICE assessment programmes, and suitable for market-oriented adoption. This collaborative approach has the potential to lead to improvements in healthcare services and the quality of life of NHS patients, by providing patients with access to the most appropriate diagnostic technologies more quickly, and by helping the NHS make best use of its resources.

The NIHR DEC Newcastle has successfully collaborated with a number of companies to help design studies and collect robust, high quality scientific evidence to support the adoption of their IVDs into the NHS. Presented examples include: (i) a clinical performance and budget impact analysis of Alere i Influenza A & B near patient test, (ii) an Academic Health Science Network funded project to validate a regional model of Familial Hypercholesterolemia diagnosis and cascade testing, and (iii) a clinical performance and budget impact analysis of Thermo Fisher Scientific’s MR-proADM for disposition planning of acutely ill patients admitted to medical admissions units.

## P35 Do prevalence expectations affect patterns of visual search and decision-making in CT colonography?

### Sue Mallett^1^, Thomas R. Fanshawe^2^, Peter Phillips^3^, Andrew Plumb^4^, Emma Helbren^4^, Steve Halligan^4^, Stuart A. Taylor^4^, Alastair Gale^3^

#### ^1^Institute of Applied Health Research, University of Birmingham, Birmingham, UK; ^2^Nuffield Department of Primary Health Care Sciences, University of Oxford, Oxford, UK; ^3^Applied Vision Research Centre, Loughborough University, Loughborough, UK; ^4^Centre for Medical Imaging, University College London, London, UK

##### **Correspondence:** Sue Mallett (s.mallett@bham.ac.uk)

**Objectives**: To use eye tracking to assess the effect of expected abnormality prevalence on visual search and decision-making during identification of colon polyps using CT Colonography (CTC).

**Background**: In clinical practice radiologists interpret images in scenarios with different expectations of disease prevalence, including both symptomatic patients and asymptomatic/screening patients. Previous research on the effect of experimentally varying prevalence have identified “rare target” effects, where at lower prevalence there are increases in target conditions missed. Previous eye tracking studies on interpretation of chest radiographs (single 2D image per patient) have shown no difference in accuracy but increased length of image scrutiny associated with increased prevalence expectations. Most research in radiological imaging includes enrichment for images with target condition, to allow feasible and timely design of multi-reader studies. Understanding of the potential effects of prevalence on radiologist interpretations amongst other sources of bias is important when applying results of prevalence enriched studies in clinical practice.

**Methods**: Thirteen radiologists interpreted endoluminal CTC 3D video fly-throughs of the same group of ten patient cases, three times each but in different random orders for each read. Abnormality prevalence was fixed (50%) but readers were told, before viewing each group, that prevalence was either 20%, 50% or 80% in the population from which cases were drawn. Infra-red eye tracking (Tobii X50 or X120) was used to record reader visual search during each 30 sec video clip. Readers indicated seeing a polyp by clicking a mouse. Multilevel modelling quantified the effect of expected prevalence on outcomes using pre-specified visual search metrics (1).

**Results**: Differences between reader visual search metrics at expected prevalences were not statistically significant for time to first pursuit of the polyp (median 0.5 s, each prevalence), pursuit rate when no polyp was on-screen (median 2.7 s-1, each prevalence) or number of mouse clicks (mean 0.75/video (20% prevalence), 0.93 (50%), 0.97 (80%)). There was weak evidence of increased tendency to look outside the central screen area at 80% prevalence, and reduction in positive polyp identifications at 20% prevalence.

**Conclusions**: This study did not find a large effect of prevalence information on most visual search metrics or polyp identification in CTC. Further research is required to quantify effects at lower prevalences and in relation to secondary outcome measures.

**Reference**

1. Mallett S, Phillips P, Fanshawe TR, Helbren E, Boone D, Gale A, Taylor SA, Manning D, Altman DG, Halligan S. Radiology 2014, 273(3):783–792.

## P36 Reporting of prognostic tumour marker studies after the introduction of the REMARK guideline needs improvement

### Sue Mallett^1^, Peggy Sekula^2^, Douglas G. Altman^3^, Willi Sauerbrei^2^

#### ^1^Institute of Applied Health Research, University of Birmingham, Birmingham, UK; ^2^Institute for Medical Biometry and Statistics, Faculty of Medicine and Medical Center – University of Freiburg, Freiburg im Breisgau, Germany; ^3^Centre for Statistics in Medicine, Nuffield Department of Orthopaedics, Rheumatology and Musculoskeletal Sciences, University of Oxford, Oxford, UK

##### **Correspondence:** Sue Mallett (s.mallett@bham.ac.uk)

**Background and aim**: Every year, thousands of articles are published on prognostic tumour markers often with contradictory results. In 2005, the REMARK guideline for reporting prognostic tumour marker studies was published. For convenience, a check list covering 20 items was provided. A review of tumor marker studies published in 2006-7 demonstrated that many lacked key information needed by readers to evaluate their reliability and clinical applicability [1]. The aim of the current study was to examine whether the quality of reporting has improved in the meantime.

**Methods**: As closely as possible, we used the methods of the earlier review of published articles from the ‘pre-REMARK’ era. This approach includes the utilization of the same data extraction form with questions representing subitems of the original items of the REMARK check list [1]. The literature search for prognostic tumour marker studies was done in Web of Science in 2013. Altogether, we assessed adherence to REMARK for 53 publications (2007 to 12) citing REMARK (‘citing group’) and 53 publications not citing REMARK (‘not-citing group’; matched by journal and issue). Descriptive comparisons over time and between groups were done with a particular focus on 10 items of the REMARK check list. Background and reasons for the restriction to 10 out of 20 items will be provided.

**Results**: Overall, the proportion of the 10 key items that were assessed slightly increased on average from 53% (range: 10% to 90%) in the earlier study to 58% (range: 30% to 100%) in the citing group and to 58% (range: 20% to 100%) in the not-citing group.

The improvement, however, was not seen in all 10 items. While an improvement was substantial for some (e.g. item 6: ‘Study design - follow up’; past study: 40%, citing group: 60%, not-citing group: 62%), it got worse for others (e.g. item 13: distribution of demographic characteristics; past study: 58%, citing group: 42%, not-citing group: 55%).

**Conclusions**: In principle, it should be easy to report all study details included in the REMARK checklist. However, our investigation shows that many items are still poorly reported, so there remains much room for improvement. To improve the clinical value of published prognosis research in cancer authors, editors and peer reviewers should be aware of and follow reporting recommendations.

**References**

[1] Mallett et al. Reporting of prognostic studies of tumour markers: A review of published articles in relation to REMARK guidelines. Br J Cancer 2010;102:173–80.

## P37 NIHR Statistics Group Imaging Studies Section: a network for statisticians and researchers using imaging in healthcare research

### Sue Mallett^1^, Thomas R. Fanshawe^2^, Julia R. Forman^3^, Susan J. Dutton^4^, Yemisi Takwoingi^1^, Elizabeth M. Hensor^5^, Thomas E. Nichols^6^

#### ^1^Institute of Applied Health Research, University of Birmingham, Birmingham, UK; ^2^Nuffield Department of Primary Health Care Sciences, University of Oxford, Oxford, UK; ^3^Cambridge Clinical Trials Unit, Cambridge University Hospitals, Cambridge, UK; ^4^Centre for Statistics in Medicine & Oxford Clinical Trials Research Unit, Nuffield Department of Orthopaedics, Rheumatology and Musculoskeletal Sciences, University of Oxford, Oxford, UK; ^5^NIHR Leeds Musculoskeletal Biomedical Research Unit & Leeds Institute of Rheumatic and Musculoskeletal Medicine, University of Leeds, Leeds, UK; ^6^Department of Statistics & Warwick Manufacturing Group, University of Warwick, UK

##### **Correspondence:** Sue Mallett (s.mallett@bham.ac.uk)

**Background**: Incorporating imaging modalities into clinical trials and healthcare research presents particular challenges: What should be measured, and how? What summary statistic should be used? How can we best handle large amounts of multiple testing? How can reliability and misdiagnosis be assessed, and what are their implications?

**Aims**: The NIHR Statistics group (http://www.statistics-group.nihr.ac.uk/) aims to promote statistical methodology, provide educational opportunities, share best practice and develop a community of statisticians funded by NIHR research units or grants.

**Activities**: The NIHR Statistics group Imaging Studies Section of the group provides a forum to address statistical issues in the design and analysis of imaging studies. The group aims to facilitate networking among statisticians, data analysts and other methodologists working in this area.

The Imaging Studies Section organises meetings every six months, with presentations on statistical challenges in imaging studies, small group discussions to share design ideas and expertise, and networking. A working group has been formed to organise meetings and facilitate related activities.

Our first meeting took place on the 22nd October 2014 at University of Oxford, attended by 25 members.

Subsequent meetings have been designed for and attended by 22 to 25 attendees, to provide small group networking and discussion as a key focus on topic specific areas

- 27th April 2015 at Warwick University in collaboration with University of Oxford, focus and discussions on inter-rater agreement and reproducibility of endpoint assessment within the context of clinical trials in Inflammatory Bowel Disease.
- 11th November 2015 at University of Birmingham in collaboration with University of Leeds, discussing statistical issues in designing a large-scale reliability exercise in ultrasonography of the joint synovium.
- 20th April 2016 at University of Oxford with attendee presentations and discussion of sample size issues in imaging studies.

This poster will outline the key statistical issues that need to be addressed when designing and analysing studies that use medical imaging, and will also summarise the current and future plans of the Imaging Studies Section.

## P38 Evidence synthesis to inform model-based cost-effectiveness evaluations of diagnostic tests: a methodological systematic review of health technology assessments

### Bethany Shinkins^1^, Yaling Yang^2^, Lucy Abel^2^, Lavinia Ferrante Di Ruffano^3^, Thomas Fanshawe^2^

#### ^1^Academic Unit of Health Economics, Leeds Institute of Health Sciences, University of Leeds, Leeds, UK; ^2^Nuffield Department of Primary Health Care Sciences, University of Oxford, Oxford, UK; ^3^Institute of Applied Health Research, University of Birmingham, Birmingham, UK

##### **Correspondence:** Bethany Shinkins (B.Shinkins@leeds.ac.uk)

**Background:** Model-based health economic evaluations of tests have become increasingly popular as they allow many different types of evidence to be considered and incorporated, in addition to facilitating estimates of cost-effectiveness beyond the duration of available study data. To parameterize a cost-effectiveness model fully, evidence on all the ways a test impacts on patient health must be included.

**Objectives:** To update an existing systematic review of the methods used to meta-analyse diagnostic accuracy studies and evaluate how the results have been incorporated into subsequent cost-effectiveness analyses.

**Methods:** HTA reports published since May 2009 were included if they: 1) evaluated a diagnostic test, 2) included a health economic evaluation and 3) included a systematic review of test accuracy. The following information was extracted from each report: 1) the types of evidence searched for and identified in systematic review(s); 2) the methods used to synthesise test accuracy data; 3) the extent to which test accuracy meta-analyses inform cost-effectiveness model parameters.

**Results:** Thirty five reports met the inclusion criteria (22 of which reported a meta-analysis). Meta-analysis of test accuracy was not feasible in 11 reviews, usually because of a limited number of studies and/or high between-study heterogeneity. Evidence on test-related outcomes (8/22) and patient outcomes (11/22) was also explicitly searched for in the systematic reviews and reported in 12/22 and 15/22 reports respectively.

The bivariate or HSROC model was implemented for in all but two reports. However, many had to resort to statistical models that do not account for the correlation between sensitivity and specificity for some secondary meta-analyses due to the small number of studies or convergence issues.

Four of the meta-analyses provided all the accuracy data required for the cost-effectiveness analysis. In fourteen reports, some of the accuracy parameters had to be informed by single studies, expert opinion, or assumptions. In the remaining four reports, the meta-analysis was not used at all to inform the cost-effectiveness analyses.

**Conclusions:** There has been a notable improvement in the uptake of statistically-appropriate meta-analysis methods for synthesising evidence on test accuracy. Prior to 2009, only 2/14 HTA reports implemented meta-analysis methods that account for the dependent relationship between sensitivity and specificity.

**Notes**

## P39 Overinterpretation and misreporting of prognostic factor studies in oncology

### Emmanuelle Kempf^1,2^, Raphael Porcher^3^, Jennifer de Beyer^1^, Karel Moons^4^, Douglas Altman^1^, Hans Reitsma^4^, Sally Hopewell^1^, Willi Sauerbrei^5^, Gary Collins^1^

#### ^1^Centre for Statistics in Medicine, Nuffield Department of Orthopaedics, Rheumatology and Musculoskeletal Sciences, University of Oxford, Oxford, UK; ^2^Department of Medical Oncology, Henri Mondor & Albert Chenevier Teaching Hospital, APHP, Créteil, France; ^3^Department of Epidemiology, Hôtel Dieu Teaching Hospital, APHP, Paris, France; ^4^Julius Center for Health Sciences and Primary Care, University Medical Center Utrecht, 3584 CG Utrecht, Netherlands; ^5^Center for Medical Biometry and Medical Informatics University of Freiburg, Freiburg, Germany

##### **Correspondence:** Gary Collins (gary.collins@csm.ox.ac.uk)

**Background** Spin, inflation or overinterpretation of study findings can be used by authors to positively exaggerate the interpretation of their findings more than the results justify.

**Objectives** To generate empirical evidence, classify the types, estimate the frequency of distorted presentation and overinterpretation of results in prognostic factor studies in medical oncology.

**Methods** We selected 17 leading oncology journals with an impact factor of 7 or more. PubMed was searched to identify primary clinical studies evaluating one or more prognostic factors (PFs) published in 2015. Each article was independently evaluated by 2 reviewers using a data extraction form that was extensively pilot-tested to identify aspects of overinterpretation. We classified them as misleading reporting, misleading interpretation and misleading extrapolation.

**Results** Our search identified 10 844 articles, of which 98 met eligibility criteria. The first author was a clinician in 61 cases and 27 studies clearly reported involving at least one statistician. The PF was assessed prospectively in 8 of 56 observational studies, and in 16 of 42 clinical trials. A for-profit funding was identified in 31 studies and REMARK guidelines were mentioned in 12 reports. The median number of PFs per study was 2 (Q1-Q3, 1-5). Overall survival was used as the outcome in 77 studies. The median number of statistical analyses reported per study with regards to the prognostic factor effect assessment was 42.5 (Q1-Q3, 15.5–86.5). Thirty-three reports reported using two or more multivariable models to assess the prognostic factor effect (as defined by the adjustment variables) and 21 did not adjust. Misleading reporting included selective and incomplete reporting of the prognostic factor effect (n = 26 and n = 8, respectively). In 32 studies where several PF effects were reported, 12 inconsistently used multiple statistical tests to assess the PF effects. The conclusions focused solely on significant results in 80 reports, and in those where there was at least one NS result, 80% of studies focused their conclusions solely on the significant results. Misleading interpretation included not using a multivariable model (such as log-rank test, correlation) to assess the PF effect (n = 25 and n = 59 in full-texts and abstracts, respectively). One out of 5 conclusions used linguistic spin with strong statements in both full-text and abstract. Linguistic spin of NS results was found in 28 Results sections in the full-text and in 18 abstract conclusions. The conclusions were inconsistent with the study findings in one out of five articles (both in the full-text and abstract). Discrepancies between the conclusions presented in the full-text and in the abstract were found in 18 reports. Clinical applicability of the prognostic factor was mentioned in 44 reports, among which the extrapolation took place in a different or an unclear clinical setting or population in 25 conclusions.

**Conclusions** Our study provides insight into the level of reporting and overinterpretation of findings that were frequently inconsistent with the results in oncology journals with high impact factors.

## P40 How response to a therapy is defined can determine outcome in stratified medicine research: q MASTERMIND study

### John Dennis^1^, Beverley Shields^2^, Angus Jones^2^, William Henley^1^, Ewan Pearson^3^, Andrew Hattersley^2^, on behalf of the MASTERMIND consortium

#### ^1^Health Statistics Group, University of Exeter Medical School, Exeter, UK; ^2^University of Exeter Medical School, Exeter, UK; ^3^University of Dundee, Dundee, UK

##### **Correspondence:** John Dennis (J.Dennis@exeter.ac.uk)

**Background:** When choosing between therapy options a stratified approach requires the identification of clearly defined patient subgroups with a common profile of response to a specific therapy. Glucose lowering therapy for Type 2 diabetes is an ideal candidate for stratified medicine as there are many drug classes available with different mechanisms of action and variable response. We examined whether the outcome of stratified medicine research is determined by how treatment response to a therapy is initially defined.

**Methods:** The study population were participants with type 2 diabetes randomised to therapy with either thiazolidinedione (TZD) or sulfonylurea (SU) in the RECORD trial, followed up over a 5 year period.

We defined treatment response as A) time for HbA1c to rise to a threshold HbA1c of ≥8.5% (the definition of failure applied in the trial) using Cox proportional hazards regression B) cumulative reduction in HbA1c from baseline over time, estimated as cumulative area under the HbA1c response curve using repeated measures mixed effects models.

We contrasted results using each definition when comparing response to the 2 therapies in 4 pre-specified subgroups defined by gender and obesity (BMI </≥30) - non-obese males (n = 468), obese males (n = 716) non-obese females (n = 264) and obese females (n = 773). These subgroups had been previously identified as candidates for stratification in analysis of routine clinical practice data (CPRD).

**Results:** Using definition A treatment response by therapy was similar for non-obese males (HR TZD vs SU 1.00, 95% CI 0.72–1.40) but was better on TZD than SU for obese males (HR 0.72, 95% CI 0.55–0.94), non-obese females (HR 0.52, 95% CI 0.32–0.84) and obese females (HR 0.52, 95% CI 0.38–0.68).

In contrast, using definition B response by therapy was better on SU than TZD for non-obese males (5 year HbA1c 7.1 mmol/mol lower, p = 0.002) but similar in obese males (HbA1c 2.2 mmol/mol lower on TZD, p = 0.30). In agreement with definition A, response was better on TZD compared to SU in non-obese females (11.2 mmol/mol lower, p < 0.0001) and obese females (18.8 mmol/mol lower, p < 0.0001).

**Conclusion:** Choice of response definition may strongly influence results when comparing the efficacy of different therapies for stratified medicines research. Studies seeking to identify subgroups with differential response to therapy should examine multiple definitions of response, and consider carefully the clinical relevance of each definition.

## P41 More than half of the TRIPOD items are inadequately reported in prediction modelling studies

### Pauline Heus^1^, Johanna A. A. G. Damen^1^, Romin Pajouheshnia^2^, Rob J. P. M. Scholten^1^, Johannes B. Reitsma^1^, Gary S. Collins^3^, Douglas G. Altman^3^, Karel G.M. Moons^2^, Lotty Hooft^1^

#### ^1^Cochrane Netherlands, Julius Center for Health Sciences and Primary Care, University Medical Center, Utrecht, The Netherlands; ^2^Julius Center for Health Sciences and Primary Care, University Medical Center, Utrecht, The Netherlands; ^3^Centre for Statistics in Medicine, NDORMS, Botnar Research Centre, University of Oxford, Oxford, UK

##### **Correspondence:** Pauline Heus (p.heus@umcutrecht.nl)

**Background**: Prediction models, both diagnostic and prognostic, are developed with the aim to guide clinical decision making. To validate, evaluate their impact and eventually use these models in clinical practice, clear and comprehensive reporting of prediction modelling studies is required. To improve the reporting of prediction models, a guideline for Transparent Reporting of a multivariable prediction model for Individual Prognosis Or Diagnosis (TRIPOD) was launched in January 2015. The TRIPOD statement is a checklist of 22 main items considered essential for informative reporting of studies developing or validating multivariable prediction models.

**Objectives**: To assess the quality of reporting of prediction modelling studies that were published before the launch of TRIPOD in 2015.

**Methods**: We selected the 10 journals with the highest impact factors within 37 clinical domains. A PubMed search was performed to identify prediction models published in May 2014. Publications that described the development and/or validation of a diagnostic or prognostic prediction model were considered eligible. We also included studies evaluating the incremental value of adding a predictor to a model. TRIPOD items were translated into a data extraction form, which was piloted extensively. Three reviewers extracted data. If they disagreed on when to consider an item “adhered”, it was discussed in consensus meetings with the other co-authors.

**Results**: Our search identified 4871 references, of which 347 potentially eligible references were assessed in full text. Eventually 148 references (within 28 clinical domains) met our eligibility criteria. Of these, 17% described diagnostic and 83% prognostic prediction models. Model development was described in 43% of the publications, validation of an existing prediction model in 26%, incremental value of adding a predictor to a model in 19% and a combination of development and validation of a model was described in 12%.

The analysis showed that overall a mean of 48.4% of the TRIPOD items (on publication level) was adhered (range 20.7%–72.4%). The mean adherence was 46.5%, 51.4%, 47.1% and 48.5% in publications about development, validation, incremental value and combination of development and validation, respectively. There was incomplete reporting of TRIPOD items concerning title and abstract, blinding, model building procedures, final model and performance measures. Source of data, eligibility criteria, study limitations and overall interpretation were adequately reported in the majority of publications.

**Conclusions**: There is room for improvement in the reporting of multivariable prediction models: more than half of the TRIPOD items are currently not or inadequately reported. Our study could serve as a baseline measurement for future research evaluating the impact of the introduction of the TRIPOD statement.

## P42 Using trial and observational data in the stratification of treatment response in Type 2 diabetes

### Beverley Shields^1^, John Dennis^1^, Angus Jones^1^, William Henley^1^, Ewan Pearson^2^, Andrew Hattersley^1^, on behalf of the MASTERMIND consortium

#### ^1^University of Exeter, Exeter, UK; ^2^University of Dundee, Dundee, UK

##### **Correspondence:** Beverley Shields (B.Shields@exeter.ac.uk)

**Background**: Stratification of therapy based on expensive testing is unlikely to be widely adopted in type 2 diabetes, as it is a common disease with relatively inexpensive treatment, and lower gains in mortality and morbidity compared with cancer. A stratified approach would need to be based on routinely available clinical criteria. Big data, in the form of population datasets such as CPRD, and industry trial data made available through Clinical Study Data Request, provide an opportunity to explore clinical predictors of response to treatment. Observational data has advantages of large sample size, but may be subject to bias and confounding. Trial data remove potential bias by randomization of therapy, but recruited patients do not necessarily reflect the “real world” setting. These datasets were not designed to answer questions of stratification “a priori” so replication is essential to ensure results are not due to chance. We aimed to use both observational and trial data to identify clinical predictors of response to second line therapy (thiazolidinediones (TZD) and sulphonylureas (SU)) in Type 2 diabetes.

**Methods**: Associations between clinical features and glycaemic response (1 year baseline-adjusted change in HbA1c) were assessed in patients treated with SU (n = 8748) and TZD (n = 8876) in UK primary care data (CPRD). Initial and longitudinal response were assessed from 3 monthly HbA1cs over 5 years in 2 randomised controlled trials of TZD v SU (ADOPT trial (TZD n = 1390; SU n = 1335) and RECORD (TZD n = 1354; SU n = 1319).

**Results**: In CPRD, obese (BMI > 30) female patients had 4.4 mmol/mol better 1 year glycemic response to TZD than SU (p < 0.001), whilst non-obese males had a 3.5 mmol/mol better response to SU than TZD (p < 0.001). These findings were replicated in ADOPT: obese females mean HbA1c 4.8 mmol/mol lower per year on TZD; non-obese males 2 mmol/mol lower per year on SU. Obese males and non-obese females both had better initial (1 year) response to SUs (3.3 mmol/mol and 2.9 mmol/mol better on SU, respectively), but over 5 years, obese males had greater response to TZD (1.13 mmol/mol) and non-obese females had a similar response to both (0.01 mmol/mol difference). Results were similar in RECORD.

**Discussion**: Glycaemic response to diabetes medications can be stratified using simple clinical criteria (gender and BMI). By replicating results in observational data and two trials we were more confident that our findings were not due to chance, bias and confounding that would be a concern if only carrying out analysis in one dataset. We propose initial analysis in observational data and replication in trials may provide a potential framework for identifying robust response strata in common disease.

## P43 How to analyse screening studies? Comparison of two analytical strategies to assess benefit

### Fueloep Scheibler, Anne Rummer, Sibylle Sturtz, Robert Großelfinger

#### Institute of Quality and Efficiency in Health Care, Cologne, Germany

##### **Correspondence:** Fueloep Scheibler (Lina.Rodenhaeuser@iqwig.de)

**Background:** Many screening questions face the problem that, due to low disease prevalences, studies need to have enormous sample sizes in order to detect statistically significant effects on patient-relevant outcomes.

**Methods:** We report on the results of an IQWiG benefit assessment of neonatal pulse oximetry screening (POS) for the detection of critical congenital heart disease (cCHD). Two different analytical strategies to assess the benefits of POS were compared: The ‘classical’ intention-to-screen (ITS) analysis using all participants as denominator and the ‘alternative’ analysis using only those participants who were affected by the disease.

**Results:** Only one concurrent controlled study (de Wahl-Granelli et al., 2009) could be included in the systematic review. Based on all 155.567 newborns (ITS analysis), the study failed to show a statistically significant effect of screening on severe acidosis (OR 0.490 [0.217; 1.109], p = 0.086). Using only babies with cCHD as denominator (n = 160), the study reported a significant effect on severe acidosis (OR: 0.268 [0.110; 0.654], p = 0.003).

**Discussion:** In most screening trials, it is useful to analyze results based on the ITS approach, because the screening interventions themselves affect the prevalence of disease (e.g. by detecting clinically insignificant cases). Thus, using only participants with the disease as denominator might introduce bias into the analysis. In the context of cCHD, however, all affected newborns are destined to die when left untreated – therefore bias is unlikely. Nevertheless, for comprehensive assessment of a screening intervention, harms have to be examined within the total target population.

**Conclusion:** It is obvious that the benefit of screening primarily evolves from the treatment of affected people. Therefore – if bias is unlikely – the demonstrated benefit in diseased people may justify the implementation of the program, even if the effect on the screened population fails to demonstrate significance. Therefore – if possible and reasonable – when evaluating screening interventions an analysis of results using the affected population as denominator seems to answer a relevant question.

## P44 Challenges in dealing with suspected disease in glaucoma diagnostic accuracy studies

### Katie Banister^1^, Craig Ramsay^1^, Augusto Azuara-Blanco^2^, Jonathan Cook^3^, Charles Boachie^4^, Jennifer Burr^5^, Manjula Kumarasamy^6^, Rupert Bourne^7^

#### ^1^Health Services Research Unit, University of Aberdeen, Aberdeen, UK; ^2^Centre for Experimental Medicine, Queen’s University Belfast, Belfast, UK; ^3^Nuffield Department of Orthopaedics, Rheumatology and Musculoskeletal Sciences, University of Oxford, Oxford, UK; ^4^Robertson Centre for Biostatistics, University of Glasgow, Glasgow, UK; ^5^School of Medicine, University of St Andrews, St Andrews, UK; ^6^Dept. of Ophthalmology, NHS Grampian, Aberdeen, UK; ^7^Vision & Eye Research Unit, Anglia Ruskin University, Cambridge, UK

##### **Correspondence:** Katie Banister (k.banister@abdn.ac.uk)

**Introduction**: Primary studies evaluating diagnostic tests encounter unique methodological challenges. To calculate the diagnostic accuracy of the new test, the reference standard results are traditionally dichotomised into two possible outcomes, target condition present or absent. Therefore these outcomes are critically dependent on the definition of disease for both the reference standard and the index test. However in some conditions where the diagnosis is based on clinical examination a diagnosis of disease suspect may be used. In striving to detect early disease, for example in glaucoma diagnosis where treatment cannot reverse existing sight loss, challenges are introduced to completing the traditional 2 × 2 table. Glaucoma suspect is a common diagnosis among individuals referred to hospital eye services. NICE recommends follow-up for glaucoma suspects. The decision as to how to handle suspected disease cases within the analysis is strongly influenced by the diagnostic question being asked. The aim of this study was to explore how suspected disease could be handled with an analysis and the impact of follow-up on diagnosis.

**Methods**: We originally conducted a paired study of the diagnostic accuracy of four imaging techniques for glaucoma in new referrals to UK NHS secondary care. The reference standard was a clinical diagnostic assessment by an experienced ophthalmologist masked to imaging results. Possible diagnoses were glaucoma; no glaucoma related findings; glaucoma suspect; ocular hypertension (OHT); primary angle closure (PAC); PAC suspect. Imaging tests gave a glaucoma classification (outside normal limits, borderline, within normal limits) or were classed as indeterminate or missing. Analyses explored the causes of indeterminate results, alternative diagnostic scenarios including indeterminate results and alternative thresholds for the tests and reference standard. A 2 year follow-up study of the glaucoma suspects was undertaken to examine the performance of the reference standard.

**Results**: Nine hundred thirty-two participants were included in the analysis of ‘worse eye’ diagnosis. Glaucoma was diagnosed in 17% of cases and no glaucoma in 32.1%. A further 26% were classified as ‘glaucoma suspect’. The remaining cases were OHT (12.3%), PAC (3.3%), PAC suspect (8.9%). In 4 cases the reference standard measurement was indeterminate (0.4%). Between 4 and 8% of imaging outputs were classed as indeterminate and this varied amongst imaging techniques. Indeterminate imaging results were further classified into low quality result; no automated classification generated; imaging artefact; patient unable to undertake test. After monitoring in secondary care the classification of glaucoma suspects was changed.

**Conclusions**: In diagnosing disease, a true diagnosis at a single point in time may not be possible and high proportion of ‘suspect’ cases may be identified who may later be confirmed as disease or no disease. Although analyses can treat the suspect cases as with or without disease, the true incidence of disease may only be determined after a period of monitoring and assessment and this may inform the optimum timing for the reference standard measurement for test accuracy studies. In considering study design, the likely proportion of ‘suspect’ cases and methods to follow-up ‘suspect’ cases should be considered.

**Funding**: NIHR HTA Programme 09/22/111 and International Glaucoma Association.

## P45 Ergonomic methods to ensure more complete information in the mapping of clinical pathways when evaluating diagnostic tests

### Ijeoma Uchegbu^1,2^, Simone Borsci^1,2^, Jennifer Murphy^3^, George Hanna^1,2^

#### ^1^The National Institute for Health Research (NIHR) Diagnostic Evidence Co-operative (DEC) London, London, UK; ^2^Surgery & Cancer, Imperial College Healthcare NHS Trust, London, UK; ^3^Imperial Clinical Trials Unit (ICTU), School of Public Health, Imperial College London, London, UK

##### **Correspondence:** Ijeoma Uchegbu (i.uchegbu@imperial.ac.uk)

Economic Evaluations can support the adoption of an intervention into the NHS. The majority of these evaluations are carried out on fully developed interventions. In the case of diagnostic tests, early evaluation (during test development) is necessary to ensure the test addresses a need; can feasibly address the identified need; and is cost effective. To enable a robust early evaluation, we have combined Human Factor (HF) - also known as Ergonomic - methods with qualitative methods to better inform the evaluation of such diagnostics.

HF is a discipline that applies various tools, theories and principles to inform the design of aspects of a system so that human interaction with this system may be optimized. We have found that combining these methods is necessary due to the indirect way in which diagnostic tests affect health outcomes. To understand and evaluate diagnostic and their relevant treatment strategies, clinical pathways must be mapped by stakeholders of the diagnostic information produced by the test. Identifying and prioritizing stakeholders may be done using a commonly used HF tool- Stakeholder Identification Tool (SIT) which enables a stakeholder’s interests and needs (as well as influence over NHS adoption) to be informed by responders.

Unlike other industries whose stakeholders are identified by the intervention’s manufacturer, diagnostic tests must be informed by those who understand the test output utility within NHS pathways. We have utilized SIT in the mapping of clinical pathways by enabling responders to identify the test role for each stakeholder type. This has better informed the selection of stakeholders to inform the mapping of clinical pathways which will be the basis of the test’s economic evaluation.

## P46 Inherent bias in clinical pathway mapping methods

### Ijeoma Uchegbu^1,2^, Alex Carter^3^, Jen Murphy^4^, Melody Ni^1,2^, Joachim Marti^3^, Julie Eatock^5^

#### ^1^The National Institute for Health Research (NIHR) Diagnostic Evidence Co-operative (DEC) London, London, UK; ^2^Surgery & Cancer, Imperial College Healthcare NHS Trust, London, UK; ^3^Institute of Global Health Innovation (IGHI), Imperial College London, London, UK; ^4^Imperial Clinical Trials Unit (ICTU), School of Public Health, Imperial College London, London, UK; ^5^Department of Computer Science, Brunel University London, London, UK

##### **Correspondence:** Ijeoma Uchegbu (i.uchegbu@imperial.ac.uk)

Clinical pathway mapping (CPM) has many uses in healthcare. For example, it is fundamental to the assessment of healthcare interventions. CPM is a heuristic technique, which by definition aims to simplify the real world. We present the limitations associated with four methods of CPM that predominantly relate to the content and construct validity of care pathway models:

- Staff interviews using champions
- Staff interviews using a Delphi method
- Starting with an NHS guidance document
- Using grounded theory approach with multiple experts

Economic evaluations of medical technologies, particularly those that have not been tested in clinical trials, are reliant on the development of accurate models; reliable forecasts of potential clinical and economic benefits are predicated on these. We aim to demonstrate which CPM method(s) is/are most appropriate for different types of research: diagnostic evaluation and health policy.

Economic evaluations require context-relevant conceptualizations of processes which allow for accurate modelling and forecasting. This immediately points to the need for CPM to be informed by stakeholders within the pathway(s) of the intervention that needs to be evaluated. As practice varies across the NHS, how possible is it to capture such processes (with reduced bias) using NHS stakeholders?

## P47 Establishing the diagnostic potential to focus during antibiotic review using a pathway mapping framework

### Ijeoma Uchegbu^1,2^, Julie Robotham^1,3^, Maria Dudareva^4^, Mark Gilchrist^4^, Alison Holmes^1,4^

#### ^1^The National Institute for Health Research (NIHR) Health Protection Research Unit in Healthcare Associated Infections and antimicrobial resistance at Imperial College, London, UK; ^2^The National Institute for Health Research (NIHR) Diagnostic Evidence Co-operative (DEC) London, London, UK; ^3^Public Health England, England, UK; ^4^Imperial College Healthcare NHS Trust, London, UK

##### **Correspondence:** Ijeoma Uchegbu (i.uchegbu@imperial.ac.uk)

Focus within the “Start Smart, Then Focus” national guidance aims to ensure prescribed antibiotics in secondary care settings are reviewed within 72 hours of initiation leading to appropriate prescribing decisions. Decisions include stopping, continuing, changing from IV to oral or changing to a completely different set of antimicrobials. To enable these decisions to be made, clinicians are often left with no further information other than the often empirical information used to initiate antimicrobials. Recent technological advances such as multiplex PCR have quickened the time to results in these environments leading to quicker identification of the causative organism and the antimicrobial(s) that it is susceptible to.

To understand the continued problems of antimicrobial review within secondary care settings which utilize such advanced technology, we carried out a service evaluation in admission wards across Imperial College Healthcare NHS Trust. The data collected was supported by short interviews (using a semi-structured questionnaire) with clinicians who had reviewed the antibiotic prescription of identified patients. All patients on adult admission wards who were initiated on antibiotics during their current admission were included in the service evaluation. Data was collected over one week in the admission wards of each of the three hospitals in the Trust in October/November 2015.

A purposeful sampling method was used to recruit clinicians for interview – clinicians involved in antibiotic review were identified and approached for interview. We aimed to identify the events which informed changes to initiated antibiotics during the service evaluation. Interviews aimed to collect the opinion of clinicians in two ways: i) what informed changes in the patient they were identified as having reviewed the prescription of and ii) what informed changes at different stages of antibiotic prescribing. We also sought to understand their thoughts on point of care tests (POCT) and how they felt POCT could improve antibiotic review.

The antibiotic prescriptions of a total of 106 patients were observed. The most frequent themes explained the reasoning behind decisions made and included the improvement of a patient’s clinical state; awaiting diagnostic information; obtaining antibiotic information; changes in diagnosis or indication; and being informed by guidelines or other diagnostic information. Using this thematic framework, we aim to scan the horizon for current and emerging diagnostics that can improve antibiotic review and evaluate the likelihood of adding value to the real issues surrounding antibiotic review within secondary care.

## P48 Is there a need for the incorporation of stakeholder usability in economic evaluations?

### Ijeoma Uchegbu^1,2^, Simone Borsci^1,2^

#### ^1^The National Institute for Health Research (NIHR) Diagnostic Evidence Co-operative (DEC) London, London, UK; ^2^Surgery & Cancer, Imperial College Healthcare NHS Trust, London, UK

##### **Correspondence:** Ijeoma Uchegbu (i.uchegbu@imperial.ac.uk)

Calculating the cost effectiveness of an intervention during an economic evaluation utilizes health outcome as well as cost information. Such information is usually sufficient when informing decision makers and budget holders of the added value that the intervention brings to specific clinical pathways. In the area of diagnostics, it is increasingly clear that diagnostic tests need to be evaluated earlier in their development process i.e. early economic evaluations. This is to ensure that the diagnostic’s route to development is appropriate and informs the current needs of the environment it aims to bring added value to.

In the early development of diagnostic tests, Human Factor analysis – also known as Ergonomics – informs manufacturers of the feasibility and usability of their test by stakeholders who will utilize said test within clinical pathways. Using such output, it is possible to establish the likelihood of use by stakeholders along a diagnostic and treatment pathway.

Incorporating this information into economic evaluations ensures that decision makers are more informed of the likely added value of a test by not assuming 100% uptake.

## P50 Targeting biomarker development in response to unmet clinical needs

### Phillip Monaghan^1^, Sarah Lord^2,3^, Andrew StJohn^4^, Sverre Sandberg^5,6,7^, Christa Cobbaert^8^, Lieselotte Lennartz^9^, Wilma Verhagen-Kamerbeek^10^, Christoph Ebert^11^, Patrick Bossuyt^12^, Andrea Horvath^13,14^ (for the Test Evaluation Working Group of the European Federation of Clinical Chemistry and Laboratory Medicine)

#### ^1^Department of Clinical Biochemistry, The Christie Pathology Partnership, The Christie NHS Foundation Trust, Manchester, UK; ^2^School of Medicine, University of Notre Dame, Notre Dame, Australia; ^3^National Health and Medical Research Council (NHMRC) Clinical Trials Centre, University of Sydney, Camperdown, Australia; ^4^ARC Consulting, Perth, Australia; ^5^The Norwegian Quality Improvement of Primary Care Laboratories (NOKLUS), Haraldsplass Deaconess Hospital, Bergen, Norway; ^6^Department of Public Health and Primary Health Care, University of Bergen, Bergen, Norway; ^7^Laboratory of Clinical Biochemistry, Haukeland University Hospital, Bergen, Norway; ^8^Department of Clinical Chemistry and Laboratory Medicine, Leiden University Medical Center, Leiden, The Netherlands; ^9^Abbott Diagnostics, Wiesbaden, Germany; ^10^Medical and Scientific Affairs, Roche Diagnostics International Ltd., Rotkreuz, Switzerland; ^11^Medical and Scientific Affairs, Roche Diagnostics GmbH, Penzberg, Germany; ^12^Department of Clinical Epidemiology, Biostatistics & Bioinformatics, Academic Medical Center, University of Amsterdam, Amsterdam, The Netherlands; ^13^SEALS Department of Clinical Chemistry and Endocrinology, Prince of Wales Hospital and School of Medical Sciences, University of New South Wales, Sydney, Australia; ^14^Screening and Test Evaluation Program, School of Public Health, University of Sydney, Camperdown, Australia

##### **Correspondence:** Phillip Monaghan (phillip.monaghan@nhs.net)

**Background**: The early introduction of biomarkers, before identifying existing gaps in clinical care and defining how the biomarker responds to unmet needs and is intended to be used to improve care, can lead to inappropriate utilization of tests and resources.

**Objective**: We aimed to define a strategy and a checklist for identifying unmet needs for new medical tests.

**Design**: A multidisciplinary working group of laboratorians, EBM, HTA, policy experts, and the IVD industry used a 4-step process: 1/ scoping literature review; 2/ eight face-to-face meetings to discuss the scope, strategy and checklist items; 3/ iterative process of feedback and consensus to develop the checklist; 4/ testing and refinement of checklist items by using case scenarios.

**Results**: Using a clinical pathway mapping approach to identify the clinical management decision linking biomarker testing to health outcomes, we developed a 14-item checklist organized in 4 domains: 1/ identify and 2/ verify the unmet clinical need; 3/ validate the intended use; and 4/ assess the feasibility of the biomarker. We present an outcome-focused approach that can be used by multiple stakeholders for any diagnostic test, irrespective of the purpose and role of testing.

**Conclusions**: The checklist intends to achieve more efficient biomarker translation and facilitate multidisciplinary collaboration by early critical assessment of the clinical pathway and potential impact of new biomarkers on health care outcomes. We propose that the checklist is field tested and validated by various stakeholder groups, and advocate the role of the laboratory professional to foster trans-sector collaboration in this regard.

**Reference**

1. Horvath, A. R., S. J. Lord, et al. (2014). “From biomarkers to medical tests: the changing landscape of test evaluation.” Clin Chim Acta 427: 49–57.

## P51 A bird’s-eye view of overdiagnosis: What’s out there?

### Kevin Jenniskens, Christiana Naaktgeboren, Johannes Reitsma, Karel Moons, Joris de Groot

#### Julius Center for Health Sciences and Primary Care, University Medical Center Utrecht, 3584 CG Utrecht, Netherlands

##### **Correspondence:** Kevin Jenniskens (k.jenniskens@umcutrecht.nl)

**Fig. 10 (abstract P51).**
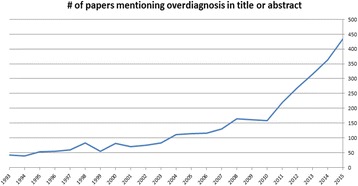
See text for description

**Introduction**: Overdiagnosis is increasingly discussed in scientific literature over the last decades (see Fig. 10). Consequences of overdiagnosis involve unnecessary expenditure of healthcare budget as well as an increased risk of side-effects or complications related to testing or subsequent treatments. In this systematic review we provide an overview of articles on the subject of overdiagnosis to gain insight into the diversity of methodological challenges faced.

**Methods**: Pubmed was searched using text words and MeSH terms related to overdiagnosis, overdetection and insignificant disease. Titles and abstracts of these papers were screened by two independent reviewers assessing which clinical domain they entailed, type of index test, type of paper (e.g. primary study, review), suggested solutions to tackle overdiagnosis and whether overdiagnosis was a dominant theme.

**Results**: Three thousand eight hundred two papers were identified. The following results are based on the 2158 most recently published papers. After exclusion of non-English papers and papers without full-text available, the titles and abstracts of 1831 papers were screened. Articles in which overdiagnosis was a dominant theme were selected, yielding 956 papers for further analysis.

There were 19,2% methodological papers related to overdiagnosis. Overdiagnosis is subject of discussion in various contexts spread across different medical disciplines. The clinical domains in which it is mostly discussed are oncology, mental disorders and infectious disease, with 60,0%, 8,2% and 5,2% respectively. The test most commonly evaluated is imaging. Overdiagnosis is most often addressed in the context of diagnostic accuracy, however also in terms of disease communication, disease definition and as a broad general topic.

**Discussion / conclusion**: A growing number of papers discuss overdiagnosis, using many different definitions. It is addressed predominantly in breast, prostate and thyroid cancer screening, however papers on the subject can be found over virtually all clinical domains. This overview can serve as a starting point for further methodological advancements in the field of overdiagnosis.

## P52 Composite evaluations of tests: multiple primary studies linked through a model. Lessons learnt from two examples

### Chris Hyde, Jaime Peters

#### Exeter Test Group, University of Exeter Medical School, Exeter, UK

##### **Correspondence:** Chris Hyde (c.j.hyde@exeter.ac.uk)

**Background**: Policy making bodies like NICE use a “linked evidence” approach where the amount of diagnostic misclassification evidenced by accuracy studies is linked using a decision-analytic or other appropriate modelling approach to the consequences of this misclassification provided by evidence on effectiveness of downstream treatments.The linkage approach could be extended to primary evaluation. In this different aspects of the impact of tests not feasibly measured in a single RCT are evaluated in separate clinical studies or using observational data, and then linked via a model. We suggest the term composite test evaluation to describe a programme of studies designed with the intent of feeding into a model which integrates the results to provide an estimate of overall effectiveness or cost-effectiveness. A preliminary version of the model may help identify aspects of impact where there is greatest uncertainty as targets for the primary studies.

**Objectives**: To reflect on two completed projects where we have employed composite test evaluation.

**Methods**: The first project concerned the effectiveness and cost-effectiveness of strategies to maximise identification of single gene diabetes (MODY), an example of stratified medicine, and the second the effectiveness and cost-effectiveness of school entry hearing screening. In a case study approach the investigators involved in developing the plans for these studies reflected on the strengths and weaknesses of composite evaluation, particularly challenges encountered in both projects.

**Main results**: Both projects provided evidence on effectiveness and cost-effectiveness in a time that was less than might be taken for an RCT, if indeed an RCT was feasible in either case. We identified many challenges, but would particularly highlight:

- Complexity of the approach
- Clearly establishing the areas of greatest uncertainty at the design stage
- Tendency to over-elaborate the model
- Justifying use of new data from clinical studies over any existing data
- Difficulty of publishing all clinical studies, resulting in loss of transparency
- Challenge of identifying this approach as being distinct from standard economic modelling

**Authors’ conclusions**: Composite evaluation of tests is a useful additional evaluative approach but is not a panacea and requires resource and time to do well.

## P53 Using expert elicitation to estimate test time duration in health technology assessment

### Bogdan Grigore, Jaime Peters, Chris Hyde

#### Exeter Test Group, University of Exeter Medical School, Exeter, UK

##### **Correspondence:** Jaime Peters (j.peters@exeter.ac.uk)

**Background**: Probabilistic decision modelling has become an important part of economic evaluations of health technologies, however informing the parameters of such models is sometimes challenging, because of insufficient data. One way to deal with a lack of data is to derive distributions from expert judgements, an approach referred to as expert elicitation.

In this study, we set out to explore the feasibility of using expert elicitation to characterise the time resource in implementing two screening tests for hearing impairment at school entry: (1) pure tone sweep audiometry (PTS) and (2) the Siemens HearCheck device (HC).

**Methodology**: Health professionals with experience in hearing impairment screening were invited to participate in the study. Experts agreeing to participate were emailed an Excel-based elicitation tool containing a training element as well as recording the opinions of the experts about the two hearing tests. Experts were also invited to comment on the format of the questions and ease of use of the tool.

**Results**: Seven experts provided their procedure time estimates for the two hearing tests. Five responses were obtained within the first four weeks of the study, while all seven responses were available after six weeks. There was good agreement among the experts regarding the duration of the two procedures. Overall, experts estimated the time taken using HC to be shorter (mean 4 minutes) than time taken using PTS (mean 7.8 minutes). None of the experts reported any difficulties in completing the questionnaire.

**Conclusions**: Expert elicitation is feasible within the context of an economic evaluation and can be conducted in a reasonable time. For the quantity of interest in this study, conducting the elicitation in the absence of a facilitator raised no issues.

## P56 Revisiting the place of diagnostic case-control studies prompted by issues raised by a comparative accuracy study of two methods of hearing screening

### Chris Hyde^1^, Obi Ukoumunne^2^, Jaime Peters^1^, Zhivko Zhelev^1^

#### ^1^Exeter Test Group, University of Exeter Medical School, Exeter, UK; ^2^Statistics Group, NIHR CLAHRC South West Peninsula (PenCLAHRC), University of Exeter Medical School, Exeter, UK

##### **Correspondence:** Chris Hyde (c.j.hyde@exeter.ac.uk)

**Background**

In diagnostic case-control, or two-gate accuracy study designs participants are drawn separately from two distinct populations. “Cases” with confirmed disease are used to estimate sensitivity, and “controls” without identifiable disease, are used to estimate specificity, predictive values not being directly estimable. With the exception of the very initial evaluation of test accuracy, diagnostic case-control studies are not recommended as a main stream primary test evaluation design as awareness has grown about the threat to validity posed by spectrum bias. Because of very low disease prevalence, we were compelled to adopt such a design, in a recent research programme on the evaluation of school entry hearing screening.

**Objectives**

To consider the advantages and disadvantages of a diagnostic case-control study we conducted, reconsidering the alternative study designs available to us.

**Methods**

We reflected on the results of the study particularly assessing the degree to which openness to bias negated our findings. The perspectives of two members of the team who were involved in developing the original protocol were balanced against the views of two researchers who had not been involved.

**Main results**

The study results were a sensitivity of 94.2% (95% CI 89.0, 97.0) for test A, and 89.0% (95% CI 82.9 to 93.1) for test B (p < 0.02). For specificity they were 82.2% (95% CI 77.7, 86.0) for test A and 86.5% (95% CI 82.5 to 90.0) for test B (p < 0.02). It is unclear whether alternative study designs to the case-control study design chosen would have been feasible. Further we identified that the bias associated with case-control studies had less impact on our main objective to compare tests, than if we had simply been interested in the values of accuracy of a single test. Nevertheless caution still needs to be exercised quantifying the difference in accuracy between the two tests. We were also able to clearly demonstrate important differences in the accuracy between cases identified by different methods directly demonstrating the important influence of population and setting on accuracy.

**Authors’ conclusions**

We think we have identified some reasons why diagnostic case-control studies should be retained as an option for mainstream study design for accuracy. The rehabilitation of this study design should include rediscovering skills in conducting diagnostic case-control studies so that if they are conducted, this is done in a way which minimises the bias to which they are clearly susceptible.

## P57 Selective cutoff reporting in studies of diagnostic test accuracy: a comparison of conventional and individual patient data meta-analyses of the patient health questionnaire-9 depression screening tool

### Brooke Levis^1,2^, Andrea Benedetti^2,3,4^, Alexander W. Levis^1,2^, John P. A. Ioannidis^5^, Ian Shrier^1,2^, Pim Cuijpers^6^, Simon Gilbody^7^, Lorie A. Kloda^8^, Dean McMillan^7^, Scott B. Patten^9^, Russell J. Steele^1,10^, Roy C. Ziegelstein^11^, Charles H. Bombardier^12^, Flavia de Lima Osório^13^, Jesse R. Fann^14^, Dwenda Gjerdingen^15^, Femke Lamers^16^, Manote Lotrakul^17^, Sonia R. Loureiro^13^, Bernd Löwe^18^, Juwita Shaaban^19^, Lesley Stafford^20^, Henk C. P. M. van Weert^21^, Mary A. Whooley^22^, Linda S. Williams^23^, Karin A. Wittkampf^24^, Albert S. Yeung^25^, Brett D. Thombs^1,2,3,26^

#### ^1^Lady Davis Institute for Medical Research, Jewish General Hospital, Montréal, Québec, Canada; ^2^Department of Epidemiology, Biostatistics and Occupational Health, McGill University, Montréal, Québec, Canada; ^3^Department of Medicine, McGill University, Montréal, Québec, Canada; ^4^Respiratory Epidemiology and Clinical Research Unit, McGill University Health Centre, Montréal, Québec, Canada; ^5^Department of Medicine, Department of Health Research and Policy, Department of Statistics, Stanford University, Stanford, California, USA; ^6^Department of Clinical, Neuro and Developmental Psychology, Vrije Universiteit (VU) University, Amsterdam, The Netherlands; ^7^Hull York Medical School and the Department of Health Sciences, University of York, Heslington, York, UK; ^8^Libraries, Concordia University, Montréal, Québec, Canada; ^9^Departments of Community Health Sciences and Psychiatry, University of Calgary, Calgary, Alberta, Canada; ^10^Department of Mathematics and Statistics, McGill University, Montréal, Québec, Canada; ^11^Department of Medicine, Johns Hopkins University School of Medicine, Baltimore, Maryland, USA; ^12^Department of Rehabilitation Medicine, University of Washington, Seattle, Washington, USA; ^13^Department of Neuroscience and Behavior, Faculty of Medicine of Ribeirão Preto, University of São Paulo, Ribeirão Preto, Brazil; ^14^Department of Psychiatry and Behavioral Sciences, University of Washington, Seattle, Washington, USA; ^15^Department of Family Medicine and Community Health, University of Minnesota, Minneapolis, Minnesota, USA; ^16^Department of Psychiatry, EMGO Institute, VU University Medical Center, Amsterdam, The Netherlands; ^17^Department of Psychiatry, Faculty of Medicine, Ramathibodi Hospital, Mahidol University, Bangkok, Thailand; ^18^Department of Psychosomatic Medicine and Psychotherapy, University Medical Center Hamburg-Eppendorf and Schön Klinik Hamburg Eilbek, Hamburg, Germany; ^19^Department of Family Medicine, School of Medical Sciences, Universiti Sains Malaysia, Kelantan, Malaysia; ^20^Centre for Women’s Mental Health, Royal Women’s Hospital, Parkville, Victoria, Australia; ^21^Department of General Practice, Academic Medical Center, University of Amsterdam, Amsterdam, The Netherlands ; ^22^Department of Veterans Affairs Medical Center, San Francisco, California, USA; ^23^Richard L Roudebush Veteran Affairs Medical Center, Health Services Research and Development, Indiana University School of Medicine, Indianapolis, Indiana, USA; ^24^Department of Psychiatry and Department of General Practice, Academic Medical Center, University of Amsterdam, Amsterdam, The Netherlands; ^25^Depression Clinical and Research Program, Massachusetts General Hospital, Boston, Massachusetts, USA; ^26^Departments of Psychiatry, Educational and Counselling Psychology, and Psychology, and School of Nursing, McGill University, Montréal, Québec, Canada

##### **Correspondence:** Brooke Levis (brooke.levis@mail.mcgill.ca)

**Background**: Selective outcome reporting in clinical trials is well understood, but has not been assessed systematically in studies of diagnostic test accuracy, where authors often report results for a small range of ordinal cutoffs around data-driven “optimal” cutoffs maximizing sensitivity and specificity.

**Objectives**: To compare traditional meta-analysis of published results to individual patient data (IPD) meta-analysis of results from all cutoffs, to: (1) assess the degree to which selective cutoff reporting exaggerates accuracy estimates, and (2) identify patterns of selective cutoff reporting.

**Methods**: Bivariate random-effects models were used to compare results of traditional and IPD meta-analysis, using studies included in a published meta-analysis of the Patient Health Questionnaire-9 (PHQ-9) depression-screening tool (Manea et al., CMAJ, 2012).

**Results**: 13 of 16 primary datasets were obtained. For the “standard” cutoff of 10, most studies (11 of 13) published accuracy results. For all other cutoffs, only 3–6 of the 13 studies published accuracy results. For all cutoffs, specificity estimates in traditional and IPD meta-analyses were within 2%. Sensitivity estimates were similar for cutoff 10, but differed by 5–15% for all other cutoffs. In samples where the PHQ-9 was poorly sensitive, authors reported results for cutoffs around the low optimal cutoff. In samples where the PHQ-9 was highly sensitive, authors reported results for cutoffs around the high optimal cutoff. Consequently, in the traditional meta-analysis (but not in the IPD meta-analysis), sensitivity increased as cutoff severity increased for part of the range of possible cutoffs. Comparing cutoff 10 across all studies, sensitivity was heterogeneous (tau-squared = 1.95). Comparing optimal cutoffs, however, sensitivity was more homogeneous (tau-squared = 0.68), but optimal cutoff values ranged from 5–15.

**Conclusion**: Selectively reporting well-performing cutoffs in small samples leads to biased estimation of accuracy in traditional meta-analyses. To reduce bias in meta-analyses, primary studies should report accuracy results for all relevant cutoffs.

## P58 Assessing the evidence used in decision models for the economic evaluation of pharmacogenetic tests

### Jaime Peters^1^, Chris Cooper^2^, James Buchanan^3^

#### ^1^Exeter Test Group, University of Exeter Medical School, Exeter, UK; ^2^PenTAG, University of Exeter Medical School, Exeter, UK; ^3^Health Economics Research Centre, University of Oxford, Oxford, UK

##### **Correspondence:** Jaime Peters (j.peters@exeter.ac.uk)

**Background**: The use and evaluation of pharmacogenetic tests is rapidly increasing, and decision models are often employed to conduct economic evaluations of these tests. These models must reflect clinical pathways for both testing and treatment, hence a great deal of evidence is often required. In parameterising these models, attention is commonly focused on treatment effectiveness and test accuracy, with systematic reviews often informing the identification of these model parameters. However, these parameters may not be the main drivers of decision models: evidence on test-related factors such as uptake, test repeats or failures and consequences of false test results may be just as, or more, important.

**Aim**: To understand what evidence is being included in decision models constructed to inform economic evaluations of pharmacogenetic tests, to describe how test-related evidence is identified and reported, and to evaluate the quality of this evidence.

**Methods**: We have undertaken a systematic search of the literature to identify published articles reporting the use of decision models to conduct economic evaluations of pharmacogenetics tests. Decision models were not restricted by type of economic evaluation, therefore cost-effectiveness, cost-utility, cost-benefit, cost-minimisation and cost-consequence analyses were all included. Information on the decision problem, the structure and perspective of the analysis, and the evidence used in the model will be extracted from each article, along with details of the use of sensitivity analyses to explore variations in test-related parameters. The quality of the decision models will also be appraised, and the use of good practice or reporting guidelines will be noted.

**Results**: Analysis is on-going, but pilot work suggests that reporting on aspects of pharmacogenetic testing in the models is poor. Moreover, details on how test-related evidence are identified, or whether there has been any form of quality appraisal are lacking. We will report on the full analysis of these articles.

**Conclusions**: This is the first review of decision models for pharmacogenetic tests to focus on the test-related evidence used in the decision models, specifically the source and quality of evidence used. Early analyses indicate that few studies provide sufficient detail on how evidence was identified for use in the model, nor is the quality of this evidence evaluated. Although sensitivity analyses are undertaken to assess the impact of test-related evidence, clarity on the importance and quality of test-related evidence is needed in published decision models.

## Poster59 Development a biomarker panel for risk stratification of patients with Barrett’s oesophagus

### Tom Nieto^1^, Claire Smith^2^, Olga Tucker^3^, Janine Dretzke^4^, Andrew Beggs^1^

#### ^1^Department of Surgery, University of Birmingham, Birmingham, UK; ^2^Birmingham Clinical Trials Unit, Institute of Applied Health Research, University of Birmingham, Birmingham, UK; ^3^Department of Surgery, Heart of England Foundation Trust, Birmingham, UK; ^4^Institute of Applied Health Research, University of Birmingham, Birmingham, UK

##### **Correspondence:** Tom Nieto (t.nieto@bham.ac.uk)

**Introduction** Chronic reflux of acid into the gullet from the stomach causes irritation to the lining of the gullet. In the presence of this irritation, the lining undergoes a change called Barrett’s Oesophagus (BO). This is a pre-cancerous condition which requires close monitoring to detect cancer of the oesophagus (gullet) in its early stages. If detected early, the cancer is easier to treat and survival is greatly improved. While less than 1 in 100 people with BO will go on to develop cancer, the incidence of oesophageal adenocarcinoma (OADC) is increasing. There is currently no robust method to identify those with BO at a high risk of developing OADC, other than regular screening using endoscopy and biopsy to ensure there is no progression. This places a large burden on screening endoscopy services and subjects patients to regular invasive endoscopic screening which may not be of benefit as a large proportion of patients will never progress to OADC.

Previous studies have shown that methylation and other epigenetic biomarkers may play a role in the identification of high risk patients with BO.

**Aim** Our aim is to develop a panel of epigenetic biomarkers to stratify patients with BO in terms of their risk of developing cellular atypia and subsequent OADC. The choice of biomarkers will be informed by a systematic review of existing evidence on the utility of epigenetic markers to predict progression.

**Method** Standard systematic review methods aimed at minimising bias will be used. Prospective and retrospective studies will be eligible if they report the association of one or more epigenetic markers with progression from BO to OADC in adults. Data obtained from the systematic review will be used where possible to generate a panel of promising epigenetic biomarkers. A laboratory validation of these biomarkers will then be conducted. Archival tissue samples from BO patients who have and have not progressed to OADC which will be compared using whole genome methylation arrays and next generation sequencing techniques to assess potential prognostic value of the biomarker panel. If validated successfully a prospective clinical trial using fresh tissue samples obtained from endoscopic biopsies will be designed.

**Results** Searches for the systematic review have been performed, and records are currently being screened for eligibility. Synthesis of results is expected to be completed by the end of June 2016, and the choice of panel finalised with laboratory validation beginning in July 2016. It is anticipated that the findings from both the systematic review and the subsequent validation study will ultimately be useful in guiding risk stratification in BO patients. This could lead to a change in current clinical practice by potentially reducing the number of invasive screening endoscopies for patients who are at lower risk of progression to OADC whilst increasing the frequency of screening for those at high risk, facilitating diagnosis of OADC at an earlier stage which can in some cases be treated with endoscopic mucosal resection rather than oesophagectomy, a procedure associated with much greater morbidity. Treating OADC at an earlier disease stage greatly improves 5 year survival rates.

## P60 Assessing the quality of systematic reviews as part of the development of diagnostic guidelines. The diagnosis of ovarian cancer: a case study

### Nirmala Rai^1^, Clare Davenport^1^, Sue Bayliss^1^, Simon Stevens^2^, Kym Snell^3^, Sue Mallet^1^, Jon Deeks^1^, Sudha Sundar^2^

#### ^1^Institute of Applied Health Research, University of Birmingham, Birmingham, UK; ^2^University of Birmingham, Birmingham, UK; ^3^Keele University, Keele, UK

##### **Correspondence:** Nirmala Rai (Raitalan@bham.ac.uk)

**Background**: Development of diagnostic guidelines may rely on the conclusions of existing diagnostic test accuracy systematic reviews (DTA reviews) for pragmatic reasons. In addition justification for undertaking a new DTA review should be based on an assessment of the quality (methodology of the review process and clinical applicability) of the existing evidence base. However, currently there is no specific tool for assessing the quality of DTA reviews.

The process of undertaking a Cochrane DTA review (Symptoms, ultrasound imaging and biochemical markers alone or in combination for the diagnosis of ovarian cancer in women with symptoms suspicious of ovarian cancer) provided the opportunity to assess the quality of a considerable existing DTA review evidence base using a quality assessment tool devised by the authors.

**Objectives**: 1) Assess the usability of a new quality assessment tool for DTA reviews. 2) Present suggested refinements to the tool following this initial pilot, for discussion.

**Methods**: Electronic databases, including Medline, Embase and Cochrane, were searched from 1991 to December 2014 for systematic reviews assessing the diagnostic test performance of symptoms, FDA approved biomarkers, ultrasound and test combinations in non-pregnant women ≥18 years suspected of ovarian cancer. Data extraction was performed in duplicate and included clinical information that may impact on test accuracy estimates (population and index test characteristics) and review quality. A quality assessment tool was derived by the authors drawing on the AMSTAR and STARD reporting standards, QUADAS-2 and current guidelines for the conduct of Cochrane DTA reviews. The tool currently comprises 3 domains, within which quality judgements are made for a number of components on: **1)Methods**: components include question formulation, search strategy, data extraction, quality assessment, statistical methods.**2)Results**: components include study flow, participant characteristics, index test characteristics, quality of included studies, test accuracy estimates.**3)Discussion**: components include review method limitations, limitations of included studies, clinical applicability.

**Results and vonclusions**: A total of 21 reviews have been included for appraisal after titles and full text screening.

Included reviews span the period from 1966 to October 2013 spearheaded from the USA, Europe and China. The reviews provide diversity in terms of index tests (symptoms, several biomarkers, different ultrasound technologies and test combinations) and included primary studies (include case control studies and retrospective and prospective studies).

The authors’ experience of using the new DTA quality assessment tool will be presented including time demands, agreement between assessors and the process of moving from documentation of review methods and making an overall assessment of quality in each of the 3 domains. Suggested refinements to the tool will be presented for discussion.

## P61 Utilizing circulating tumour cell (CTC) counts to optimize systemic therapy of metastatic prostate cancer: a phase III randomised trial

### Emma Hall, Nuria Porta, David Lorente Estelles, Johann de Bono, on behalf of the CTC-STOP protocol development group

#### The Institute of Cancer Research, London, UK

##### **Correspondence:** CTC-STOP protocol development group (CTC-STOP-icrctsu@icr.ac.uk)

**Background**: Assessment of treatment response in routine clinical practice in castrate resistant prostate cancer (CRPC) is a major challenge. In patients with bone only disease, response evaluation criteria in solid tumours (RECIST) are not usually useful and whilst consensus criteria based on PSA, clinical and radiological biomarkers are available they are used inconsistently. Most clinicians rely on clinical symptoms to drive treatment switch decisions suggesting the need for more precise biomarkers. Shedding of tumour cells into the circulation is a necessary step for the formation of metastases. Multiple assays and devices are available to detect, isolate, enumerate and characterise circulating tumour cells (CTC) and having demonstrated analytical validity and clinical validity in clinical trials, the CELLSEARCH®(Janssen Diagnostics, LLC) system has regulatory clearance as an aid in monitoring patients, with metastatic breast, colorectal and castrate resistant prostate (CRPC) cancers. In metastatic CRPC, CTC count is prognostic for survival and CTC count decreases have met the statistical requirements for surrogacy of overall survival.

**Objective**: To determine if the use of serial CTC counts can direct early discontinuation of 1st line chemotherapy in patients with metastatic CRPC without adversely impacting overall survival, when compared with standard approaches to guide treatment switch decisions.

**Methods**: CTC-STOP is a multicentre phase III trial in which metastatic CRPC patients with CTC count ≥5 cells/7.5 mL are randomised 1:1 to either standard of care (control) or CTC guided treatment (intervention). Serial blood samples for central CTC enumeration are taken during treatment. In the intervention group, if a patient has two successive CTC determinations showing progression by CTCs (defined as either (1) failure to achieve both a 30% decline from baseline and a conversion from “unfavourable” (≥5 cells/7.5 mL) to “favourable” (<5 cells/7.5 mL) or, after a CTC response, either (2) conversion from favourable to unfavourable CTC count or (3) a 30% increase in CTCs from nadir and an unfavourable CTC count), the treating clinician will receive a recommendation from the trial Chief Investigator to discontinue 1st line chemotherapy and commence 2nd line chemotherapy at the following cycle of treatment. All patients will complete at least 3 cycles of standard 1st line chemotherapy before any CTC-guided treatment recommendation is made. In the control group, clinicians will not be made aware of CTC count results. The trial has a non-inferiority design and is powered to exclude a 20% increase in mortality (i.e. hazard ratio not worse than 1.20) in patients whose treatment management has been based on CTC values. Target sample size is 1178 patients. A feasibility analysis after accrual of 200 patients will evaluate recruitment rates and adherence to CTC-guided treatment recommendations. The trial will open to recruitment in Q2 2016.

**Impact**: Earlier decision-making based on circulating biomarkers could minimise morbidity and cost without adversely impacting outcome. This may result in decreased toxicity, reduced health care economic costs and may enable a higher proportion of patients to receive further lines of active treatment.

