# Erratum to: Methods for evaluating medical tests and biomarkers

**DOI:** 10.1186/s41512-017-0011-4

**Published:** 2017-03-30

**Authors:** Gowri Gopalakrishna, Miranda Langendam, Rob Scholten, Patrick Bossuyt, Mariska Leeflang, Anna Noel-Storr, James Thomas, Iain Marshall, Byron Wallace, Penny Whiting, Clare Davenport, Mariska Leeflang, Gowri GopalaKrishna, Isabel de Salis, Sue Mallett, Robert Wolff, Penny Whiting, Richard Riley, Marie Westwood, Jos Kleinen, Gary Collins, Hans Reitsma, Karel Moons, Antonia Zapf, Annika Hoyer, Katharina Kramer, Oliver Kuss, J. Ensor, J. J. Deeks, E. C. Martin, R. D. Riley, Gerta Rücker, Susanne Steinhauser, Martin Schumacher, Richard Riley, Joie Ensor, Kym Snell, Brian Willis, Thomas Debray, Karel Moons, Jon Deeks, Gary Collins, Lavinia Ferrante di Ruffano, Brian Willis, Clare Davenport, Sue Mallett, Sian Taylor-Phillips, Chris Hyde, Jon Deeks, Sue Mallett, Stuart A. Taylor, Gauraang Batnagar, STREAMLINE COLON Investigators, STREAMLINE LUNG Investigators, METRIC Investigators, Sian Taylor-Phillips, Lavinia Ferrante Di Ruffano, Farah Seedat, Aileen Clarke, Jon Deeks, Sarah Byron, Frances Nixon, Rebecca Albrow, Thomas Walker, Carla Deakin, Chris Hyde, Zhivko Zhelev, Harriet Hunt, Lavinia Ferrante di Ruffano, Yaling Yang, Lucy Abel, James Buchanan, Thomas Fanshawe, Bethany Shinkins, Laure Wynants, Jan Verbakel, Sabine Van Huffel, Dirk Timmerman, Ben Van Calster, Mariska Leeflang, Aeliko Zwinderman, Patrick Bossuyt, Jason Oke, Jack O’Sullivan, Rafael Perera, Brian Nicholson, Hannah L. Bromley, Tracy E. Roberts, Adele Francis, Denniis Petrie, G. Bruce Mann, Kinga Malottki, Holly Smith, Jon Deeks, Lucinda Billingham, Alice Sitch, Sue Mallett, Jon Deeks, Oke Gerke, Mie Holm-Vilstrup, Eivind Antonsen Segtnan, Ulrich Halekoh, Poul Flemming Høilund-Carlsen, Bernard G. Francq, Jon Deeks, Alice Sitch, Jac Dinnes, Julie Parkes, Walter Gregory, Jenny Hewison, Doug Altman, William Rosenberg, Peter Selby, Julien Asselineau, Paul Perez, Aïssatou Paye, Emilie Bessede, Cécile Proust-Lima, Christiana Naaktgeboren, Joris de Groot, Anne Rutjes, Patrick Bossuyt, Johannes Reitsma, Karel Moons, Gary Collins, Emmanuel Ogundimu, Jonathan Cook, Yannick Le Manach, Doug Altman, Laure Wynants, Yvonne Vergouwe, Sabine Van Huffel, Dirk Timmerman, Ben Van Calster, Romin Pajouheshnia, Rolf Groenwold, Karen Moons, Johannes Reitsma, Linda Peelen, Ben Van Calster, Daan Nieboer, Yvonne Vergouwe, Bavo De Cock, Micael J. Pencina, Ewout W. Steyerberg, Jennifer Cooper, Sian Taylor-Phillips, Nick Parsons, Chris Stinton, Steve Smith, Andy Dickens, Rachel Jordan, Alexandra Enocson, David Fitzmaurice, Alice Sitch, Peymane Adab, Bernard G. Francq, Charles Boachie, Gaj Vidmar, Karoline Freeman, Martin Connock, Sian Taylor-Phillips, Rachel Court, Aileen Clarke, Joris de Groot, Christiana Naaktgeboren, Hans Reitsma, Carl Moons, Jessica Harris, Andrew Mumford, Zoe Plummer, Kurtis Lee, Barnaby Reeves, Chris Rogers, Veerle Verheyden, Gianni D. Angelini, Gavin J. Murphy, Jeremy Huddy, Melody Ni, Katherine Good, Graham Cooke, Patrick Bossuyt, George Hanna, Jie Ma, Doug Altman, Gary Collins, K. G. M. (Carl) Moons, Joris A. H. de Groot, Sue Mallett, Doug G. Altman, Johannes B. Reitsma, Gary S. Collins, Karel G. M. Moons, Douglas G. Altman, Johannes B. Reitsma, Gary S. Collins, Adina Najwa Kamarudin, Ruwanthi Kolamunnage-Dona, Trevor Cox, Melody Ni, Jeremy Huddy, Simone Borsci, George Hanna, Teresa Pérez, M.Carmen Pardo, Angel Candela-Toha, Alfonso Muriel, Javier Zamora, Sabina Sanghera, Syed Mohiuddin, Richard Martin, Jenny Donovan, Joanna Coast, Mikyung Kelly Seo, John Cairns, Elizabeth Mitchell, Alison Smith, Judy Wright, Peter Hall, Michael Messenger, Nicola Calder, Nyantara Wickramasekera, Karen Vinall-Collier, Andrew Lewington, Romin Pajouheshnia, Johanna Damen, Rolf Groenwold, Karel Moons, Linda Peelen, Michael Messenger, David Cairns, Alison Smith, Michelle Hutchinson, Judy Wright, Peter Hall, Nicola Calder, Cathie Sturgeon, Liz Mitchel, Rebecca Kift, Sofia Christakoudi, Manohursingh Rungall, Paula Mobillo, Rosa Montero, Tjir-Li Tsui, Sui Phin Kon, Beatriz Tucker, Steven Sacks, Chris Farmer, Terry Strom, Paramit Chowdhury, Irene Rebollo-Mesa, Maria Hernandez-Fuentes, Johanna A. A. G. Damen, Thomas P. A. Debray, Pauline Heus, Lotty Hooft, Karel G. M. Moons, Romin Pajouheshnia, Johannes B. Reitsma, Rob J. P. M. Scholten, Johanna A. A. G. Damen, Lotty Hooft, Ewoud Schuit, Thomas P. A. Debray, Gary S. Collins, Ioanna Tzoulaki, Camille M. Lassale, George C. M. Siontis, Virginia Chiocchia, Corran Roberts, Michael Maia Schlüssel, Stephen Gerry, James A. Black, Pauline Heus, Yvonne T. van der Schouw, Linda M. Peelen, Karel G. M. Moons, Johanna A. A. G. Damen, Thomas P. A. Debray, Pauline Heus, Lotty Hooft, Karel G. M. Moons, Romin Pajouheshnia, Johannes B. Reitsma, Rob J. P. M. Scholten, Jie Ma, Doug Altman, Gary Collins, Graeme Spence, David McCartney, Ann van den Bruel, Daniel Lasserson, Gail Hayward, Werner Vach, Antoinette de Jong, Coreline Burggraaff, Otto Hoekstra, Josée Zijlstra, Henrica de Vet, Harriet Hunt, Chris Hyde, Sara Graziadio, Joy Allen, Louise Johnston, Rachel O’Leary, Michael Power, Joy Allen, Sara Graziadio, Louise Johnson, Rachel O’Leary, Michael Power, Ray Waters, John Simpson, Louise Johnston, Joy Allen, Sara Graziadio, Rachel O’Leary, Ray Waters, Michael Power, Sue Mallett, Thomas R. Fanshawe, Peter Phillips, Andrew Plumb, Emma Helbren, Steve Halligan, Stuart A. Taylor, Alastair Gale, Sue Mallett, Peggy Sekula, Douglas G. Altman, Willi Sauerbrei, Sue Mallett, Thomas R. Fanshawe, Julia R. Forman, Susan J. Dutton, Yemisi Takwoingi, Elizabeth M. Hensor, Thomas E. Nichols, Bethany Shinkins, Yaling Yang, Lucy Abel, Lavinia Ferrante Di Ruffano, Thomas Fanshawe, Emmanuelle Kempf, Raphael Porcher, Jennifer de Beyer, Karel Moons, Douglas Altman, Hans Reitsma, Sally Hopewell, Willi Sauerbrei, Gary Collins, John Dennis, Beverley Shields, Angus Jones, William Henley, Ewan Pearson, Andrew Hattersley, on behalf of the MASTERMIND consortium, Pauline Heus, Johanna A. A. G. Damen, Romin Pajouheshnia, Rob J. P. M. Scholten, Johannes B. Reitsma, Gary S. Collins, Douglas G. Altman, Karel G. M. Moons, Lotty Hooft, Beverley Shields, John Dennis, Angus Jones, William Henley, Ewan Pearson, Andrew Hattersley, on behalf of the MASTERMIND consortium, Fueloep Scheibler, Anne Rummer, Sibylle Sturtz, Robert Großelfinger, Katie Banister, Craig Ramsay, Augusto Azuara-Blanco, Jonathan Cook, Charles Boachie, Jennifer Burr, Manjula Kumarasamy, Rupert Bourne, Ijeoma Uchegbu, Simone Borsci, Jennifer Murphy, George Hanna, Ijeoma Uchegbu, Alex Carter, Jen Murphy, Melody Ni, Joachim Marti, Julie Eatock, Ijeoma Uchegbu, Julie Robotham, Maria Dudareva, Mark Gilchrist, Alison Holmes, Ijeoma Uchegbu, Simone Borsci, Phillip Monaghan, Sarah Lord, Andrew StJohn, Sverre Sandberg, Christa Cobbaert, Lieselotte Lennartz, Wilma Verhagen-Kamerbeek, Christoph Ebert, Patrick Bossuyt, Andrea Horvath, for the Test Evaluation Working Group of the European Federation of Clinical Chemistry and Laboratory Medicine, Kevin Jenniskens, Christiana Naaktgeboren, Johannes Reitsma, Karel Moons, Joris de Groot, Chris Hyde, Jaime Peters, Bogdan Grigore, Jaime Peters, Chris Hyde, Chris Hyde, Obi Ukoumunne, Jaime Peters, Zhivko Zhelev, Brooke Levis, Andrea Benedetti, Alexander W. Levis, John P. A. Ioannidis, Ian Shrier, Pim Cuijpers, Simon Gilbody, Lorie A. Kloda, Dean McMillan, Scott B Patten, Russell J. Steele, Roy C Ziegelstein, Charles H. Bombardier, Flavia de Lima Osório, Jesse R. Fann, Dwenda Gjerdingen, Femke Lamers, Manote Lotrakul, Sonia R Loureiro, Bernd Löwe, Juwita Shaaban, Lesley Stafford, Henk C. P. M. van Weert, Mary A. Whooley, Linda S. Williams, Karin A. Wittkampf, Albert S. Yeung, Brett D. Thombs, Jaime Peters, Chris Cooper, James Buchanan, Tom Nieto, Claire Smith, Olga Tucker, Janine Dretzke, Andrew Beggs, Nirmala Rai, Clare Davenport, Sue Bayliss, Simon Stevens, Kym Snell, Sue Mallet, Jon Deeks, Sudha Sundar, Emma Hall, Nuria Porta, David Lorente Estelles, Johann de Bono, on behalf of the CTC-STOP protocol development group

**Affiliations:** 1 Department of Clinical Epidemiology, Biostatistics & Bioinformatics, Academic Medical Center, University of Amsterdam, Amsterdam, The Netherlands; 2 Cochrane Netherlands, Julius Center for Health Sciences and Primary Care, University Medical Center Utrecht, Utrecht, Netherlands; 3 Cochrane Dementia and Cognitive Improvement Group, University of Oxford, Oxford, UK; 4 EPPI-Centre, Department of Social Science, University College London, London, UK; 5 Division of Health and Social Care Research, King’s College London, London, UK; 6 College of Computer and Information Science, Northeastern University, Boston, USA; 7 University Hospitals Bristol NHS Foundation Trust, School of Social and Community Medicine, Bristol, UK; 8 Institute of Applied Health Research, University of Birmingham, Birmingham, UK; 9 Department of Clinical Epidemiology, Biostatistics & Bioinformatics, Academic Medical Center, University of Amsterdam, Amsterdam, The Netherlands; 10 School of Social and Community Medicine, University of Bristol, Bristol, UK; 11 Institute of Applied Health Research, University of Birmingham, Birmingham, UK; 12 Kleijnen Systematic Reviews Ltd, York, UK; 13 University Hospitals Bristol NHS Foundation Trust, School of Social and Community Medicine, Bristol, UK; 14 Research Institute for Primary Care and Health Sciences, Keele University, Keele, UK; 15 Centre for Statistics in Medicine, Nuffield Department of Orthopaedics, Rheumatology and Musculoskeletal Sciences, University of Oxford, Oxford, UK; 16 Julius Center for Health Sciences and Primary Care, University Medical Center Utrecht, Utrecht, The Netherlands; 17 Cochrane Netherlands, University Medical Center Utrecht, Utrecht, The Netherlands; 18 Department of Medical Statistics, University Medical Center Göttingen, Göttingen, Germany; 19 Institute for Biometry and Epidemiology, German Diabetes Center, Leibniz Institute for Diabetes Research at Heinrich Heine University, Düsseldorf, Germany; 20 Research Institute for Primary Care and Health Sciences, Keele University, Keele, UK; 21 Institute of Applied Health Research, University of Birmingham, Birmingham, UK; 22 Manchester Pharmacy School, University of Manchester, Manchester, UK; 23 Institute for Medical Biometry and Statistics, Faculty of Medicine and Medical Center – University of Freiburg, Stefan-Meier-Str. 26, 79104 Freiburg, Germany; 24 Institute of Medical Statistics, Informatics and Epidemiology, University of Cologne, Kerpener Str. 62, 50937 Cologne, Germany; 25 Research Institute for Primary Care and Health Sciences, Keele University, Keele, UK; 26 Keele University, Keele, UK; 27 Institute of Applied Health Research, University of Birmingham, Birmingham, UK; 28 University of Utrecht, Utrecht, Netherlands; 29 Julius Center for Health Sciences and Primary Care, University Medical Center Utrecht, 3584 CG Utrecht, Netherlands; 30 Centre for Statistics in Medicine, Nuffield Department of Orthopaedics, Rheumatology and Musculoskeletal Sciences, University of Oxford, Oxford, UK; 31 Institute of Applied Health Research, University of Birmingham, Birmingham, UK; 32 Division of Health Sciences, Warwick Medical School, The University of Warwick, Coventry, UK; 33 Exeter Test Group, University of Exeter Medical School, Exeter, UK; 34 Institute of Applied Health Research, University of Birmingham, Birmingham, UK; 35 Centre for Medical Imaging, University College London, London, UK; 36 Division of Health Sciences, Warwick Medical School, The University of Warwick, Coventry, UK; 37 Institute of Applied Health Research, University of Birmingham, Birmingham, UK; 38 University of Warwick, Coventry, UK; 39 National Institute for Health and Care Excellence, Diagnostic Assessment Programme, Manchester, UK; 40 Exeter Test Group, University of Exeter Medical School, Exeter, UK; 41 Institute of Applied Health Research, University of Birmingham, Birmingham, UK; 42 Nuffield Department of Primary Health Care Sciences, University of Oxford, Oxford, UK; 43 Academic Unit of Health Economics, Leeds Institute of Health Sciences, University of Leeds, Leeds, UK; 44 KU Leuven Department of Electrical Engineering (ESAT), STADIUS Center for Dynamical Systems, Signal Processing and Data Analytics, Leuven, Belgium; 45 KU Leuven iMinds Department Medical Information Technologies, Leuven, Belgium; 46 University of Oxford, Nuffield Department of Primarcy Care Health Sciences, Oxford, UK; 47 KU Leuven Department of Development and Regeneration, Leuven, Belgium; 48 Department of Public Health, Erasmus MC, Rotterdam, Netherlands; 49 Department of Clinical Epidemiology, Biostatistics & Bioinformatics, Academic Medical Center, University of Amsterdam, Amsterdam, The Netherlands; 50 Nuffield Department of Primary Care Health Sciences, University of Oxford, Oxford, UK; 51 Department of Health Economics, University of Birmingham, Birmingham, UK; 52 Department of Breast Surgery, Nuffield House, University Hospital Birmingham, Birmingham, UK; 53 Melbourne School of Population and Global Health, University of Melbourne, Parkville, Australia; 54 Department of Breast Surgery, Royal Women’s Hospital, Melbourne, Australia; 55 Cancer Research UK Clinical Trials Unit (CRCTU), Institute of Cancer and Genomic Sciences, University of Birmingham, Birmingham, UK; 56 Institute of Applied Health Research, University of Birmingham, Birmingham, UK; 57 Institute of Applied Health Research, University of Birmingham, Birmingham, UK; 58 Department of Nuclear Medicine, Odense University Hospital, Odense, Denmark; 59 Epidemiology, Biostatistics and Biodemography, University of Southern Denmark, Odense, Denmark; 60 Department of Nuclear Medicine, Odense University Hospital & Department of Clinical Research, University of Southern Denmark, Odense, Denmark; 61 Robertson Centre for Biostatistics, University of Glasgow, Glasgow, UK; 62 Institute of Applied Health Research, University of Birmingham, Birmingham, UK; 63 University of Southampton, Southampton, UK; 64 University of Leeds, Leeds, UK; 65 Centre for Statistics in Medicine, Nuffield Department of Orthopaedics, Rheumatology and Musculoskeletal Sciences, University of Oxford, Oxford, UK; 66 University College London, London, UK; 67 Bordeaux University Hospital, Public Health Department, Clinical Epidemiology Unit and CIC 1401 EC, Bordeaux, France; 68 French National Reference Center for Campylobacter and Helicobacter, Bordeaux, France; 69 INSERM U1219, Bordeaux Population Health Research Center, Bordeaux, France; Univ. Bordeaux, ISPED, Bordeaux, France; 70 Julius Center for Health Sciences and Primary Care, University Medical Center Utrecht, 3584 CG Utrecht, Netherlands; 71 CTU Bern, Department of Clinical Research, University of Bern, Bern, Switzerland; 72 Institute of Social and Preventive Medicine, University of Bern, Bern, Switzerland; 73 Department of Clinical Epidemiology and Biostatistics, Academic Medical Center, University of Amsterdam, Amsterdam, Netherlands; 74 Centre for Statistics in Medicine, Nuffield Department of Orthopaedics, Rheumatology and Musculoskeletal Sciences, University of Oxford, Oxford, UK; 75 Departments of Anesthesia & Clinical Epidemiology and Biostatistics, Michael DeGroote School of Medicine, Faculty of Health Sciences, McMaster University and the Perioperative Research Group, Population Health Research Institute, Hamilton, Canada; 76 KU Leuven Department of Electrical Engineering (ESAT), STADIUS Center for Dynamical Systems, Signal Processing and Data Analytics, Leuven, Belgium; 77 KU Leuven iMinds Department Medical Information Technologies, Leuven, Belgium; 78 Center for Medical Decision Sciences, Department of Public Health, Erasmus MC, Rotterdam, The Netherlands; 79 KU Leuven Department of Development and Regeneration, Leuven, Belgium; 80 Department of Public Health, Erasmus MC, Rotterdam, Netherlands; 81 Julius Center for Health Sciences and Primary Care, University Medical Center Utrecht, Utrecht, The Netherlands; 82 Cochrane Netherlands, University Medical Center Utrecht, Utrecht, The Netherlands; 83 KU Leuven, Department of Development and Regeneration, Leuven, Belgium; 84 Department of Public Health, Erasmus MC, Rotterdam, Netherlands; 85 Duke Clinical Research Institute, Duke University, Durham (NC), USA; Department of Biostatistics and Bioinformatics, Duke University, Durham, NC USA; 86 Division of Health Sciences, Warwick Medical School, University of Warwick, Coventry, UK; 87 Midlands and North West Bowel Cancer Screening Hub, Hospital of St Cross, University Hospitals Coventry and Warwickshire NHS Trust, Rugby, UK; 88 Institute of Applied Health Research, University of Birmingham, Birmingham, UK; 89 Robertson Centre for Biostatistics, University of Glasgow, Glasgow, UK; 90 University Rehabilitation Institute, Ljubljana, Slovenia; 91 Faculty of Medicine, University of Ljubljana, Ljubljana, Slovenia; 92 University of Primorska, Koper, Slovenia; 93 Division of Health Sciences, Warwick Medical School, The University of Warwick, Coventry, UK; 94 Julius Center for Health Sciences and Primary Care, University Medical Center Utrecht, 3584 CG Utrecht, Netherlands; 95 Clinical Trials and Evaluation Unit, University of Bristol, Bristol, UK; 96 School of Cellular and Molecular Medicine, University of Bristol, Bristol, UK; 97 University Hospitals Bristol NHS Foundation Trust, Bristol, UK; 98 School of Clinical Sciences, University of Bristol, Bristol, UK; 99 Department of Clinical Sciences, University of Leicester, Leicester, UK; 100 NIHR-Diagnostic Evidence Cooperative, Imperial College London, London, UK; 101 Department of Anaesthetics, Imperial College Healthcare NHS Trust, London, UK; 102 Division of Infectious Diseases, Imperial College, London, UK; 103 Department of Clinical Epidemiology, Biostatistics & Bioinformatics, Academic Medical Center, University of Amsterdam, Amsterdam, The Netherlands; 104 Centre for Statistics in Medicine, Nuffield Department of Orthopaedics, Rheumatology and Musculoskeletal Sciences, University of Oxford, Oxford, UK; 105 Julius Center for Health Sciences and Primary Care, University Medical Center Utrecht, 3584 CG Utrecht, Netherlands; 106 Institute of Applied Health Research, University of Birmingham, Birmingham, UK; 107 Centre for Statistics in Medicine, Nuffield Department of Orthopaedics, Rheumatology and Musculoskeletal Sciences, University of Oxford, Oxford, UK; 108 Cochrane Netherlands, University Medical Center Utrecht, Utrecht, The Netherlands; 109 Julius Center for Health Sciences and Primary Care, University Medical Center Utrecht, 3584 CG Utrecht, Netherlands; 110 Centre for Statistics in Medicine, Nuffield Department of Orthopaedics, Rheumatology and Musculoskeletal Sciences, University of Oxford, Oxford, UK; 111 Cochrane Netherlands, University Medical Center Utrecht, Utrecht, The Netherlands; 112 Department of Biostatistics, University of Liverpool, Liverpool, UK; 113 NIHR-Diagnostic Evidence Cooperative, Imperial College London, London, UK; 114 Department of Statistics and OR III, Complutense University of Madrid, Madrid, Spain; 115 Department of Statistics and OR I, Complutense University of Madrid, Madrid, Spain; 116 Department of Anesthesia and Reanimation, Ramón y Cajal Hospital (IRYCIS), Madrid, Spain; 117 Department of Clinical Biostatistics, Ramón y Cajal Hospital (IRYCIS), Madrid, Spain; 118 Department of Clinical Biostatistics, Ramón y Cajal Hospital (IRYCIS), Madrid, Spain; 119 Barts and The London School of Medicine and Dentistry, Queen Mary University, London, UK; 120 Health Economics at Bristol (HEB), School of Social and Community Medicine, University of Bristol, Bristol, UK; 121 School of Social and Community Medicine, University of Bristol, Bristol, UK; 122 London School of Hygiene and Tropical Medicine, London, UK; 123 University of Bergen, Bergen, Norway; 124 Leeds Institute of Health Sciences, University of Leeds, Leeds, UK; 125 NIHR Diagnostic Evidence Co-Operative Leeds , Leeds Institute of Health Sciences, University of Leeds, Leeds, UK; 126 The Institute of Genetics and Molecular Medicine, Edinburgh Cancer Research Centre, University of Edinburgh, Edinburgh, UK; 127 Leeds Teaching Hospitals NHS Trust, St James’s University Hospital, Leeds, UK; 128 School of Dentistry, University of Leeds, Leeds, UK; 129 Julius Center for Health Sciences and Primary Care, University Medical Center Utrecht, Utrecht, The Netherlands; 130 Cochrane Netherlands, University Medical Center Utrecht, Utrecht, The Netherlands; 131 NIHR Diagnostic Evidence Co-Operative Leeds, Leeds Teaching Hospitals NHS Trust, St James’s University Hospital, Leeds, UK; 132 Leeds Institute of Clinical Trials Research, University of Leeds, Leeds, UK; 133 Leeds Institute of Health Sciences, University of Leeds, Leeds, UK; 134 Leeds Institute of Cancer and Pathology, University of Leeds, Leeds, UK; 135 The Institute of Genetics and Molecular Medicine, Edinburgh Cancer Research Centre, University of Edinburgh, Edinburgh, UK; 136 UK NEQAS [Edinburgh]. Department of Laboratory Medicine, Royal Infirmary, Edinburgh, UK; 137 Department of Blood Sciences, Old Medical School, Leeds General Infirmary, Great George Street, Leeds, UK; 138 MRC Centre for Transplantation, DTIMB, King’s College London, London, UK; 139 NIHR Comprehensive Biomedical Research Centre at Guy’s and St Thomas’ NHS Foundation Trust in partnership with King’s College London and King’s College Hospital, London, UK; 140 Guy’s and St Thomas’ NHS Foundation Trust, London, UK; 141 King’s College Hospital NHS Foundation Trust, London, UK; 142 East Kent Hospitals University NHS Foundation Trust, Canterbury, UK; 143 Department of Medicine, Transplant Institute, Beth Israel Deaconess Medical Center, Harvard Medical School, Boston, MA USA; 144 Department of Biostatistics, Institute of Psychiatry, Psychology and Neuroscience, King’s College London, London, UK; 145 Julius Center for Health Sciences and Primary Care, and Cochrane Netherlands, University Medical Center Utrecht, Utrecht, The Netherlands; 146 Cochrane Netherlands, Julius Center for Health Sciences and Primary Care, University Medical Center Utrecht, Utrecht, The Netherlands; 147 Julius Center for Health Sciences and Primary Care, University Medical Center Utrecht, 3584 CG Utrecht, Netherlands; 148 Julius Center for Health Sciences and Primary Care, and Cochrane Netherlands, University Medical Center Utrecht, Utrecht, The Netherlands; 149 Cochrane Netherlands, Julius Center for Health Sciences and Primary Care, University Medical Center Utrecht, Utrecht, The Netherlands; 150 Centre for Statistics in Medicine, Nuffield Department of Orthopaedics, Rheumatology and Musculoskeletal Sciences, University of Oxford, Oxford, UK; 151 Department of Epidemiology and Biostatistics, School of Public Health, Imperial College London, London, UK; 152 Department of Cardiology, Bern University Hospital, 3010 Bern, Switzerland; 153 MRC Epidemiology Unit, University of Cambridge School of Clinical Medicine, Cambridge, UK; 154 Julius Center for Health Sciences and Primary Care, University Medical Center Utrecht, Utrecht, Netherlands; 155 Julius Center for Health Sciences and Primary Care, and Cochrane Netherlands, University Medical Center Utrecht, Utrecht, The Netherlands; 156 Cochrane Netherlands, Julius Center for Health Sciences and Primary Care, University Medical Center Utrecht, Utrecht, The Netherlands; 157 Julius Center for Health Sciences and Primary Care, University Medical Center Utrecht, 3584 CG Utrecht, Netherlands; 158 Centre for Statistics in Medicine, Nuffield Department of Orthopaedics, Rheumatology and Musculoskeletal Sciences, University of Oxford, Oxford, UK; 159 Nuffield Department of Primary Care Health Sciences, University of Oxford, Oxford, UK; 160 Nuffield Department of Medicine, University of Oxford, Oxford, UK; 161 Institute for Medical Biometry and Statistics, Medical Faculty and Medical Center, University of Freiburg, Freiburg im Breisgau, Germany; 162 Department of Radiology and Nuclear Medicine, University Medical Center, Utrecht, The Netherlands; 163 Department of Hematology, VU University Medical Center, Amsterdam, The Netherlands; 164 Department of epidemiology and biostatistics, VU University Medical Center, Amsterdam, The Netherlands; 165 Exeter Test Group, University of Exeter Medical School, Exeter, UK; 166 NIHR Diagnostic Evidence Co-operative Newcastle, Newcastle upon Tyne Hospitals NHS Foundation Trust, Newcastle upon Tyne, UK; 167 NIHR Diagnostic Evidence Co-operative Newcastle, Newcastle University, Newcastle upon Tyne, UK; 168 NIHR Diagnostic Evidence Co-operative Newcastle, Newcastle University, Newcastle upon Tyne, UK; 169 NIHR Diagnostic Evidence Co-operative Newcastle, Newcastle upon Tyne Hospitals NHS Foundation Trust, Newcastle upon Tyne, UK; 170 NIHR Diagnostic Evidence Co-operative Newcastle, Newcastle University, Newcastle upon Tyne, UK; 171 NIHR Diagnostic Evidence Co-operative Newcastle, Newcastle upon Tyne Hospitals NHS Foundation Trust, Newcastle upon Tyne, UK; 172 Institute of Applied Health Research, University of Birmingham, Birmingham, UK; 173 Nuffield Department of Primary Health Care Sciences, University of Oxford, Oxford, UK; 174 Applied Vision Research Centre, Loughborough University, Loughborough, UK; 175 Centre for Medical Imaging, University College London, London, UK; 176 Institute of Applied Health Research, University of Birmingham, Birmingham, UK; 177 Institute for Medical Biometry and Statistics, Faculty of Medicine and Medical Center – University of Freiburg, Freiburg im Breisgau, Germany; 178 Centre for Statistics in Medicine, Nuffield Department of Orthopaedics, Rheumatology and Musculoskeletal Sciences, University of Oxford, Oxford, UK; 179 Institute of Applied Health Research, University of Birmingham, Birmingham, UK; 180 Nuffield Department of Primary Health Care Sciences, University of Oxford, Oxford, UK; 181 Cambridge Clinical Trials Unit, Cambridge University Hospitals, Cambridge, UK; 182 Centre for Statistics in Medicine & Oxford Clinical Trials Research Unit, Nuffield Department of Orthopaedics, Rheumatology and Musculoskeletal Sciences, University of Oxford, Oxford, UK; 183 NIHR Leeds Musculoskeletal Biomedical Research Unit & Leeds Institute of Rheumatic and Musculoskeletal Medicine, University of Leeds, Leeds, UK; 184 Department of Statistics & Warwick Manufacturing Group, University of Warwick, Warwick, UK; 185 Academic Unit of Health Economics, Leeds Institute of Health Sciences, University of Leeds, Leeds, UK; 186 Nuffield Department of Primary Health Care Sciences, University of Oxford, Oxford, UK; 187 Institute of Applied Health Research, University of Birmingham, Birmingham, UK; 188 Centre for Statistics in Medicine, Nuffield Department of Orthopaedics, Rheumatology and Musculoskeletal Sciences, University of Oxford, Oxford, UK; 189 Department of Medical Oncology, Henri Mondor & Albert Chenevier Teaching Hospital, APHP, Créteil, France; 190 Department of Epidemiology, Hôtel Dieu Teaching Hospital, APHP, Paris, France; 191 Julius Center for Health Sciences and Primary Care, University Medical Center Utrecht, 3584 CG Utrecht, Netherlands; 192 Center for Medical Biometry and Medical Informatics, University of Freiburg, Freiburg, Germany; 193 Health Statistics Group, University of Exeter Medical School, Exeter, UK; 194 University of Exeter Medical School, Exeter, UK; 195 University of Dundee, Dundee, UK; 196 Cochrane Netherlands, Julius Center for Health Sciences and Primary Care, University Medical Center, Utrecht, The Netherlands; 197 Julius Center for Health Sciences and Primary Care, University Medical Center, Utrecht, The Netherlands; 198 Centre for Statistics in Medicine, NDORMS, Botnar Research Centre, University of Oxford, Oxford, UK; 199 University of Exeter, Exeter, UK; 200 University of Dundee, Dundee, UK; 201 Institute of Quality and Efficiency in Health Care, Cologne, Germany; 202 Health Services Research Unit, University of Aberdeen, Aberdeen, UK; 203 Centre for Experimental Medicine, Queen’s University Belfast, Belfast, UK; 204 Nuffield Department of Orthopaedics, Rheumatology and Musculoskeletal Sciences, University of Oxford, Oxford, UK; 205 Robertson Centre for Biostatistics, University of Glasgow, Glasgow, UK; 206 School of Medicine, University of St Andrews, St Andrews, UK; 207 Department of Ophthalmology, NHS Grampian, Aberdeen, UK; 208 Vision & Eye Research Unit, Anglia Ruskin University, Cambridge, UK; 209 The National Institute for Health Research (NIHR) Diagnostic Evidence Co-operative (DEC) London, London, UK; 210 Surgery & Cancer, Imperial College Healthcare NHS Trust, London, UK; 211 Imperial Clinical Trials Unit (ICTU), School of Public Health, Imperial College London, London, UK; 212 The National Institute for Health Research (NIHR) Diagnostic Evidence Co-operative (DEC), London, UK; 213 Surgery & Cancer, Imperial College Healthcare NHS Trust, London, UK; 214 Institute of Global Health Innovation (IGHI), Imperial College London, London, UK; 215 Imperial Clinical Trials Unit (ICTU), School of Public Health, Imperial College London, London, UK; 216 Department of Computer Science, Brunel University London, London, UK; 217 The National Institute for Health Research (NIHR) Health Protection Research Unit in Healthcare Associated Infections and antimicrobial resistance at Imperial College, London, UK; 218 The National Institute for Health Research (NIHR) Diagnostic Evidence Co-operative (DEC) London, London, UK; 219 Public Health England, England, UK; 220 Imperial College Healthcare NHS Trust, London, UK; 221 The National Institute for Health Research (NIHR) Diagnostic Evidence Co-operative (DEC) London, London, UK; 222 Surgery & Cancer, Imperial College Healthcare NHS Trust, London, UK; 223 Department of Clinical Biochemistry, The Christie Pathology Partnership, The Christie NHS Foundation Trust, Manchester, UK; 224 School of Medicine, University of Notre Dame, Notre Dame, Australia; 225 National Health and Medical Research Council (NHMRC) Clinical Trials Centre, University of Sydney, Camperdown, Australia; 226 ARC Consulting, Perth, Australia; 227 The Norwegian Quality Improvement of Primary Care Laboratories (NOKLUS), Haraldsplass Deaconess Hospital, Bergen, Norway; 228 Department of Public Health and Primary Health Care, University of Bergen, Bergen, Norway; 229 Laboratory of Clinical Biochemistry, Haukeland University Hospital, Bergen, Norway; 230 Department of Clinical Chemistry and Laboratory Medicine, Leiden University Medical Center, Leiden, The Netherlands; 231 Abbott Diagnostics, Wiesbaden, Germany; 232 Medical and Scientific Affairs, Roche Diagnostics International Ltd, Rotkreuz, Switzerland; 233 Medical and Scientific Affairs, Roche Diagnostics GmbH, Penzberg, Germany; 234 Department of Clinical Epidemiology, Biostatistics & Bioinformatics, Academic Medical Center, University of Amsterdam, Amsterdam, The Netherlands; 235 SEALS Department of Clinical Chemistry and Endocrinology, Prince of Wales Hospital and School of Medical Sciences, University of New South Wales, Sydney, Australia; 236 Screening and Test Evaluation Program, School of Public Health, University of Sydney, Camperdown, Australia; 237 Julius Center for Health Sciences and Primary Care, University Medical Center Utrecht, 3584 CG Utrecht, Netherlands; 238 Exeter Test Group, University of Exeter Medical School, Exeter, UK; 239 Exeter Test Group, University of Exeter Medical School, Exeter, UK; 240 Exeter Test Group, University of Exeter Medical School, Exeter, UK; 241 Statistics Group, NIHR CLAHRC South West Peninsula (PenCLAHRC), University of Exeter Medical School, Exeter, UK; 242 Lady Davis Institute for Medical Research, Jewish General Hospital, Montréal, Québec Canada; 243 Department of Epidemiology, Biostatistics and Occupational Health, McGill University, Montréal, Québec Canada; 244 Department of Medicine, McGill University, Montréal, Québec Canada; 245 Respiratory Epidemiology and Clinical Research Unit, McGill University Health Centre, Montréal, Québec Canada; 246 Department of Medicine, Department of Health Research and Policy, Department of Statistics, Stanford University, Stanford, California USA; 247 Department of Clinical, Neuro and Developmental Psychology, Vrije Universiteit (VU) University, Amsterdam, The Netherlands; 248 Hull York Medical School and the Department of Health Sciences, University of York, Heslington, York UK; 249 Libraries, Concordia University, Montréal, Québec Canada; 250 Departments of Community Health Sciences and Psychiatry, University of Calgary, Calgary, Alberta Canada; 251 Department of Mathematics and Statistics, McGill University, Montréal, Québec Canada; 252 Department of Medicine, Johns Hopkins University School of Medicine, Baltimore, Maryland USA; 253 Department of Rehabilitation Medicine, University of Washington, Seattle, Washington USA; 254 Department of Neuroscience and Behavior, Faculty of Medicine of Ribeirão Preto, University of São Paulo, Ribeirão Preto, Brazil; 255 Department of Psychiatry and Behavioral Sciences, University of Washington, Seattle, Washington USA; 256 Department of Family Medicine and Community Health, University of Minnesota, Minneapolis, Minnesota USA; 257 Department of Psychiatry, EMGO Institute, VU University Medical Center, Amsterdam, The Netherlands; 258 Department of Psychiatry, Faculty of Medicine, Ramathibodi Hospital, Mahidol University, Bangkok, Thailand; 259 Department of Psychosomatic Medicine and Psychotherapy, University Medical Center Hamburg-Eppendorf and Schön Klinik Hamburg Eilbek, Hamburg, Germany; 260 Department of Family Medicine, School of Medical Sciences, Universiti Sains Malaysia, Kelantan, Malaysia; 261 Centre for Women’s Mental Health, Royal Women’s Hospital, Parkville, Victoria Australia; 262 Department of General Practice, Academic Medical Center, University of Amsterdam, Amsterdam, The Netherlands; 263 Department of Veterans Affairs Medical Center, San Francisco, California USA; 264 Richard L Roudebush Veteran Affairs Medical Center, Health Services Research and Development, Indiana University School of Medicine, Indianapolis, Indiana USA; 265 Department of Psychiatry and Department of General Practice, Academic Medical Center, University of Amsterdam, Amsterdam, The Netherlands; 266 Depression Clinical and Research Program, Massachusetts General Hospital, Boston, Massachusetts USA; 267 Departments of Psychiatry, Educational and Counselling Psychology, and Psychology, and School of Nursing, McGill University, Montréal, Québec Canada; 268 Exeter Test Group, University of Exeter Medical School, Exeter, UK; 269 PenTAG, University of Exeter Medical School, Exeter, UK; 270 Health Economics Research Centre, University of Oxford, Oxford, UK; 271 Department of Surgery, University of Birmingham, Birmingham, UK; 272 Birmingham Clinical Trials Unit, Institute of Applied Health Research, University of Birmingham, Birmingham, UK; 273 Department of Surgery, Heart of England Foundation Trust, Birmingham, UK; 274 Institute of Applied Health Research, University of Birmingham, Birmingham, UK; 275 Institute of Applied Health Research, University of Birmingham, Birmingham, UK; 276 University of Birmingham, Birmingham, UK; 277 Keele University, Keele, UK; 278 The Institute of Cancer Research, London, UK

## Erratum

The original MEMTAB Abstracts in Diagnostic and Prognostic Research contains the incorrect year on individual abstracts in the PDF [1].

“Diagnostic and Prognostic Research 2016” under the correspondence line should therefore have been written as “Diagnostic and Prognostic Research 2017” as the journal did not launch until 2017.
